# Estimated number of eligible Part B beneficiaries for the medicare diabetes prevention program at the county level and by urban–rural classification

**DOI:** 10.1371/journal.pone.0241757

**Published:** 2020-11-10

**Authors:** Boon Peng Ng, Yiling J. Cheng, Stephanie Rutledge, Michael J. Cannon, Ping Zhang, Bryce D. Smith

**Affiliations:** 1 College of Nursing and Disability, Aging and Technology Cluster, University of Central Florida, Orlando, Florida, United States of America; 2 Division of Diabetes Translation, Centers for Disease Control and Prevention, Atlanta, Georgia, United States of America; University of Utah, UNITED STATES

## Abstract

**Introduction:**

Diabetes imposes large health and financial burdens on Medicare beneficiaries. Type 2 diabetes can be prevented or delayed through lifestyle modification programs. In 2018, Medicare began to offer the Medicare Diabetes Prevention Program (MDPP), a lifestyle intervention, to eligible beneficiaries nationwide. The number of MDPP-eligible beneficiaries is not known, but this information is essential in efforts to expand the program and increase enrollment. This study aimed to estimate the number and spatial variation of MDPP-eligible Part B beneficiaries at the county level and by urban–rural classification.

**Methods:**

Data from 2011–2016 National Health and Nutrition Examination Surveys and a survey-weighted logistic regression model were used to estimate proportions of prediabetes in the United States by sex, age, and race/ethnicity based on the MDPP eligibility criteria. The results from the predictive model were applied to 2015 Medicare Part B beneficiaries to estimate the number of MDPP-eligible beneficiaries. The National Center for Health Statistics’ Urban–Rural Classification Scheme for Counties from 2013 were used to define urban and rural categories.

**Results:**

An estimated 5.2 million (95% CI = 3.5–7.0 million) Part B beneficiaries were eligible for the MDPP. By state, estimates ranged from 13,000 (95% CI = 8,500–18,000) in Alaska to 469,000 (95% CI = 296,000–641,000) in California. There were 2,149 counties with ≤1,000 eligible beneficiaries and 11 with >25,000. Consistent with demographic patterns, urban counties had more eligible beneficiaries than rural counties.

**Conclusions:**

These estimates could be used to plan locations for new MDPPs and reach eligible Part B beneficiaries for enrollment.

## Introduction

According to the *National Diabetes Statistics Report*, *2017*, approximately 25% of U.S. adults aged 65 years or older have diabetes, and 48% (23.1 million) have prediabetes [1]. Medicare spent about $42 billion on diabetes in 2016 [2]. Type 2 diabetes, which accounts for 90%–95% of all diabetes cases, can be prevented or delayed through some lifestyle modification programs [3–5].

Medicare began to offer Medicare Diabetes Prevention Program (MDPP) services to eligible beneficiaries nationwide in 2018 [2]. To be eligible for the Medicare Diabetes Prevention Program (MDPP), Medicare beneficiaries must have the following [2]:

- Enrollment in Part B (original Medicare, fee-for-service plan) or Part C (Medicare Advantage plan);
- No previous diagnosis of diabetes;
- No end-stage renal disease (ESRD);
- Meet 1 of 3 blood test requirements:
  - Hemoglobin A1c test with a value of 5.7%–6.4%;
  - Fasting plasma glucose test with a value of 110–125 mg/dl; or
  - Oral glucose tolerance test with a value of 140–199 mg/dl;
- Have a body mass index (BMI) ≥25 (≥23 for Asians);
- Have not received MDPP services before

The MDPP is part of the National Diabetes Prevention Program led by the Centers for Disease Control and Prevention (CDC). It uses a CDC-approved curriculum consisting of 12 months of sessions on dietary change, increased physical activity, and behavioral strategies to achieve modest weight loss [2, 3].

To increase enrollment in the MDPP, decision makers need estimates of the number of eligible beneficiaries at local levels. Geographic-based data, such as county-level estimates and maps, can be useful for visualizing these estimates [6, 7]. The authors found only one study that estimated the prevalence of prediabetes in California for adults aged 55 years or older at the county level. Prevalence ranged from 41% to 71%, with an average of 60% [8]. Therefore, the objective of this study was to estimate and map the number of MDPP-eligible Part B beneficiaries for all U.S. counties by urban–rural classification.

## Materials and methods

### Data

This study used data from the 2015 Medicare Administrative Research Files [9] and the 2011–2016 National Health and Nutrition Examination Surveys (NHANES) [10]. The 2015 Medicare Administrative Research Files contain enrollment and claims data of all (100%) Part B beneficiaries at the individual level [9, 11]. NHANES is an ongoing nationally representative survey of noninstitutionalized civilians that provided demographic and laboratory data [10]. (Both were the most current data at the time of the study).

This retrospective observational study used NHANES to estimate proportions of prediabetes among adults aged 65 years or older that met BMI requirements based on MDPP’s eligibility definition. The estimated proportions of prediabetes from the predictive model were then applied to the number of Medicare beneficiaries who met the other MDPP eligibility criteria (except for prediabetes status) to estimate the number of Medicare beneficiaries eligible for the MDPP.

### Study population

The study population was 18.4 million Medicare Part B beneficiaries aged 65 years or older. The study population included was based on MDPP eligibility criteria [2] with selected indicators in the Medicare Master Beneficiary Summary File [9]. More specifically, Medicare beneficiaries enrolled at least one month in Part B who had no previous diagnosis of diabetes and no ESRD (MDPP eligibility criteria discussed above). The study excluded beneficiaries with full Part C coverage and missing demographic/geographic information. Part C encounter data is not available to identify those with diabetes and/or ESRD at the time of the study, therefore they were excluded from the study.

### Measures

The MDPP’s prediabetes definition was used to identify prediabetes status using NHANES 2011–2016’s laboratory and demographic data. Those without diabetes and a body mass index ≥25 (≥23 for Asians) were classified as having prediabetes if they had any of the following: fasting plasma glucose levels of 110–125 mg/dL, A1c of 5.7%–6.4%, or a 2-hour, post-oral glucose tolerance test glucose level of 140–199 mg/dl [2].

### Statistical analysis

NHANES 2011–2016’s demographic and laboratory data and a survey-weighted logistic model were used to estimate proportions of prediabetes by sex (male, female), age (65–69, 70–74, ≥75 years), and race/ethnicity (white non-Hispanic, black non-Hispanic, Other). NHANES used a clustered multi-stage complex sample design, the relative weights (interview weight or 2-hour oral glucose tolerance test weight) and sampling design variables were used to account for the survey design [10].

**Fig 1:**
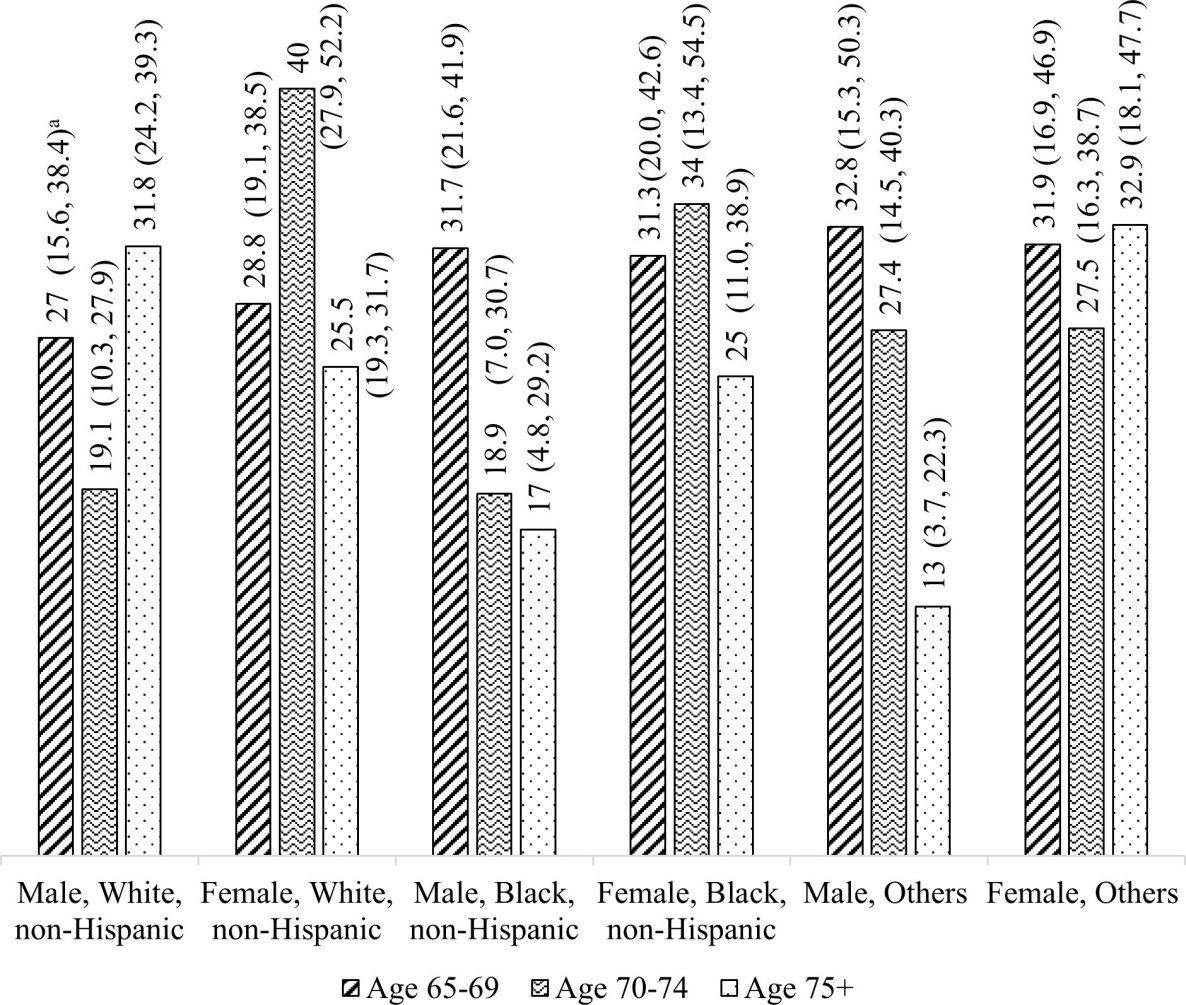
Estimated proportions of prediabetes among older adults by sex, age, and race/ethnicity, 2011–2016 NHANES. Note: We observed that, for some racial or ethnic groups, the prevalence of prediabetes went down with age, which is consistent with Khan and colleagues’ findings [13] and is likely due to people transitioning from prediabetes to diabetes as they get older. ^a^ Numbers in parentheses represent 95% Confidence Interval.

Guided by a past approach [12], the estimated proportion of MDPP’s prediabetes and associated 95% confidence interval (CI) (Fig 1) were then multiplied by the study population of 18.4 million Medicare Part B beneficiaries to estimate the number of MDPP-eligible Part B beneficiaries in each sex/age/race-ethnicity group. For example, we multiplied the 19.1% prediabetes prevalence rate of our estimate for male, 70–74 years, and white non-Hispanic by the corresponding number of Medicare Part B beneficiaries (male, 70–74 years, and white non-Hispanic who have met the other MDPP eligibility criteria, except for prediabetes status) to estimate the number of male, 70–74 years, and white non-Hispanic beneficiaries who are eligible for MDPP.

Estimates were then stratified by state and county level using the state indicator and the 5-digit Federal Information Processing Standards (FIPS) codes [14] from Medicare enrollment data. Additionally, the FIPS codes were used to create maps for the urban–rural classification. Urban–rural categories were based on prior studies [15, 16] (noncore and micropolitan were defined as rural counties; small metro, medium metro, large fringe metro, and large central metro were defined as urban counties) using the National Center for Health Statistics’ Urban–Rural Classification Scheme for Counties from 2013 [17].

We also estimated the prevalence (or MDPP eligible Part B beneficiaries per 1,000) and density (MDPP eligible Part B beneficiaries per square mile of land area) of MDPP eligible Part B beneficiaries. Additionally, we calculated the proportions of Part B beneficiaries (enrolled at least one month in Part B) per our study population of Medicare beneficiaries by county. Results are presented in the S1 File. Data analyses and maps were created using SAS Enterprise 7.1, Stata/SE version 15.1, and ArcGIS Desktop 10.5.1. in 2018–2019.

## Results

Fig 1 shows proportions of prediabetes by sex, age, and race/ethnicity that were used to estimate MDPP-eligible Part B beneficiaries, ranging from 13% for those who were male, ≥75 years, and Other to 40% for those who were female, 70–74 years, and white non-Hispanic.

An estimated 5.2 million (95% CI = 3.5–7.0 million) Part B beneficiaries were eligible for the MDPP. At the state level, the highest numbers were found in California (469,000, 95% CI = 296,000–641,000), Texas (355,000, 95% CI = 230,000–480,000) and Florida (349,000, 95% CI = 232,000–466,000). The lowest numbers were found in the District of Columbia (DC) (8,600, 95% CI = 5,100–12,000), Alaska (13,000, 95% CI = 8,500–18,000), and Rhode Island (16,000, 95% CI = 10,700–21,000). For the U.S. territories of Puerto Rico and the Virgin Islands, the numbers were 7,600 (95% CI = 3,800–11,300) and 2,500 (95% CI = 1,300–3,600), respectively (S1 Table in S1 File).

**Fig 2:**
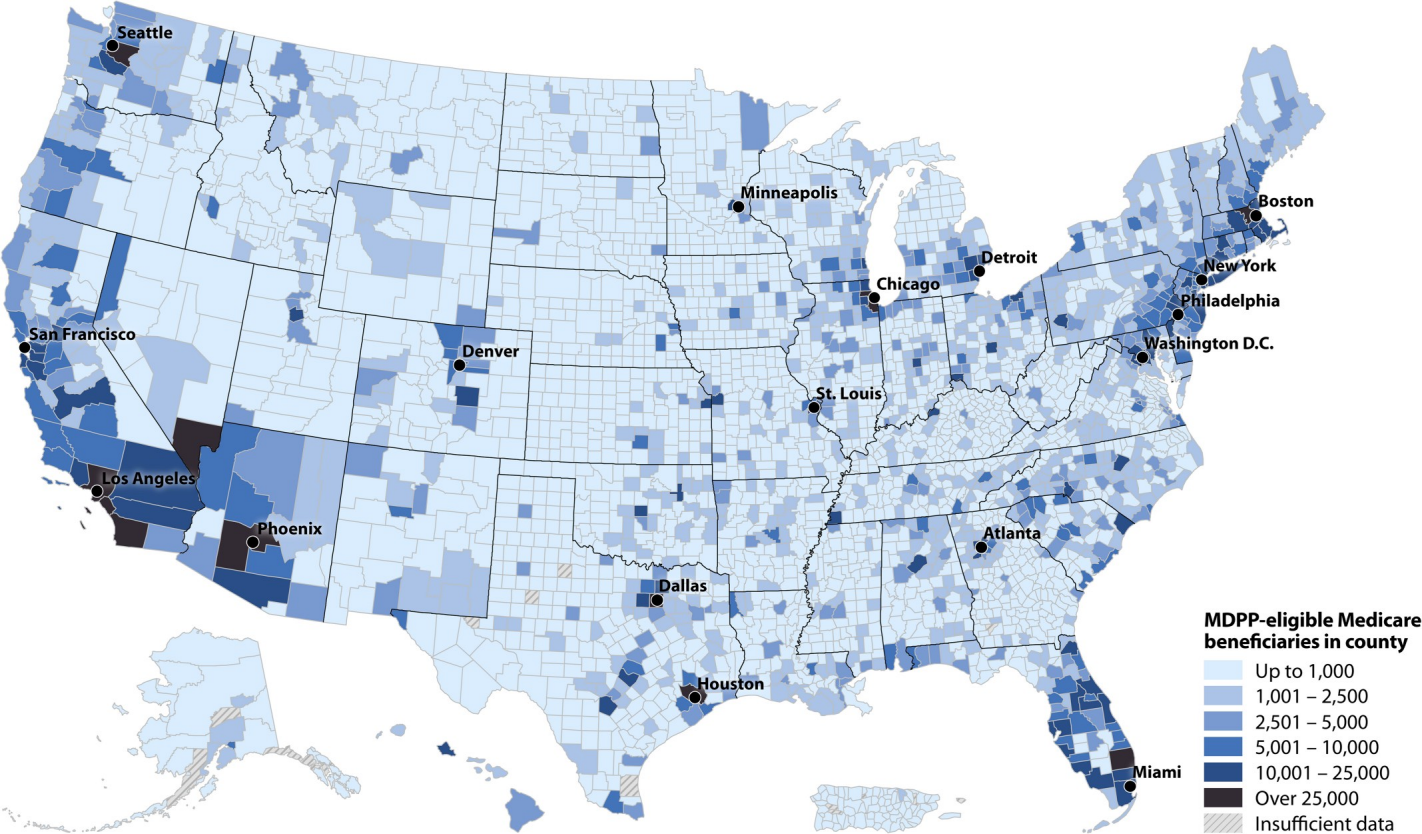
Estimated number of MDPP-eligible beneficiaries by county level, 2015.

**Fig 3:**
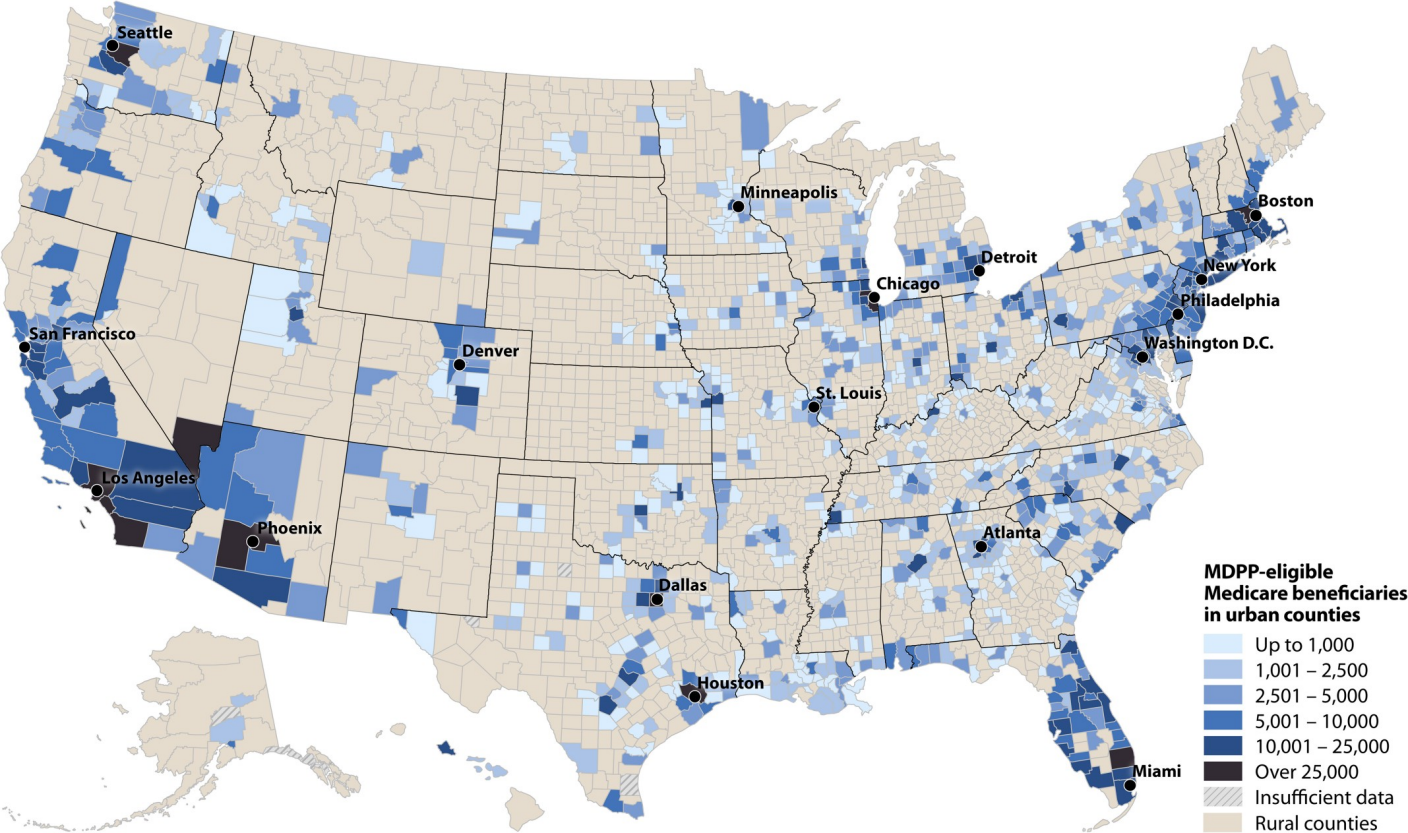
Estimated number of MDPP-eligible beneficiaries in urban counties, 2015.

**Fig 4:**
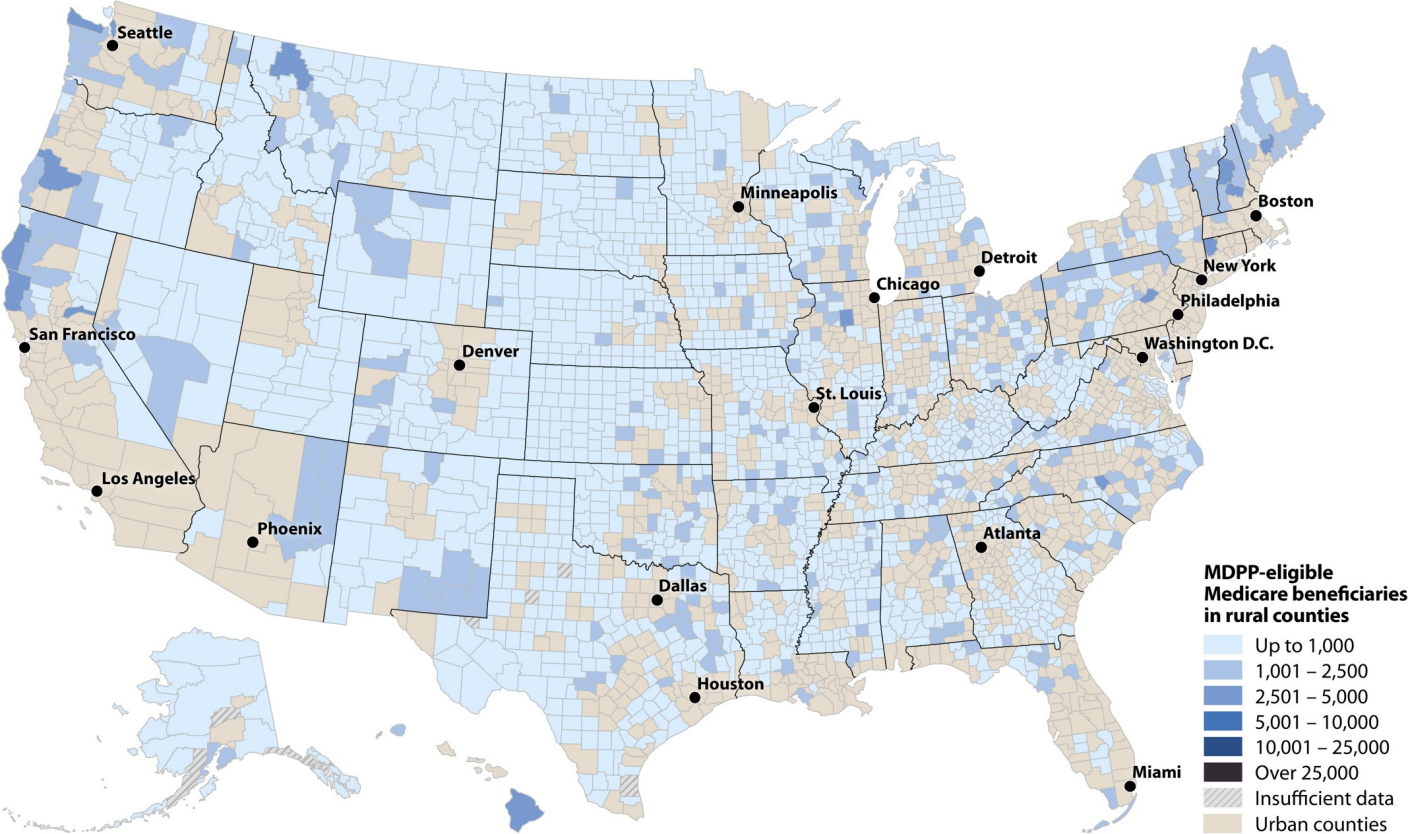
Estimated number of MDPP-eligible beneficiaries in rural counties, 2015.

At the county level, the 10 counties with the most eligible Part B beneficiaries were Los Angeles, California (CA); Cook, Illinois; Maricopa, Arizona; Harris, Texas (TX); San Diego, CA; Orange, CA; King, Washington; Dallas, TX; Middlesex, Massachusetts; and Palm Beach, Florida, for an estimated total of 461,000. There were 2,149 counties (68%) with ≤1,000 eligible beneficiaries and 11 (<1%) with >25,000 (Fig 2). Consistent with demographic patterns, urban and more densely populated counties had more eligible beneficiaries than rural counties (Figs 3 and 4 and S2 Table in S1 File).

The cartographic boundary files/shapefile used to create the map images were retrieved from the U.S. Census Bureau (https://www.census.gov/geographies/mapping-files/time-series/geo/carto-boundary-file.2015.html)

## Discussion

These estimates show that the majority of eligible Part B beneficiaries are concentrated in more densely populated urban counties. This distribution aligns with the 2015 Medicare Current Beneficiary Survey Public Use File [18], in which ~80% of Medicare beneficiaries reported living in a metro area (calculated by authors, S1 Calculation in S1 File). This finding suggests limited travel distances to programs for most eligible beneficiaries living in a county with a program, although geographical area varies greatly by county. In addition, ~73% of beneficiaries reported driving themselves to the doctor’s office (S1 Calculation in S1 File).

Compared to previous estimates of prediabetes in California, county-level estimates in this study were much lower: 28.7% for beneficiaries 65 years or older versus 57% of adults aged 55 years or older in Los Angeles County [8]. Differences can be attributed in part to the MDPP eligibility criteria, age distribution, and different data sources used. However, the current estimate is consistent with the overall prediabetes estimate for adults aged 65 years or older in the *National Diabetes Statistics Report*, *2017* [1].

This study also showed that > 60% of U.S. counties had ≤ 1,000 eligible Part B beneficiaries, possibly limiting the practicality of multiple program locations in these counties. Decision makers could use these estimates to support using available resources to focus on people living in rural areas and perhaps provide transportation services. Additionally, opportunities to use innovative technology such as online DPP to deliver in rural communities can be considered. Moin and colleagues examined results from a trial of an online DPP found that an online DPP intervention achieved a similar weight loss goal compared to in-person DPP [19]. However, more research is needed to better understand the effectiveness and cost-effectiveness of virtual/on-line programs [20].

Estimates of prevalence (ranging from 27.2 in the Virgin Islands and 27.9 in DC to 28.7 in Arizona, Delaware, and New Hampshire and 28.9 in Puerto Rico) and density (ranging from 0.02 eligible beneficiaries per square mile of land area in Alaska to 142.13 in DC) are provided in the S1 and S2 Tables in S1 File to allow for relative interpretations of data. However, decision makers are more likely interested in the number of MDPP-eligible Part B beneficiaries than prevalence to determine resource allocation for expanding the MDPP program and increasing enrollment for MDPP.

### Limitations

There are several limitations to this study. First, the numbers of individuals with prediabetes were not directly calculated from the Medicare data. Although ICD-9-CM codes can be used to identify prediabetes, previous studies show when both lab results and ICD-9-CM codes were used to identify prediabetes, using ICD-9-CM codes alone could severely underestimate the number of individuals with prediabetes compared to the lab results [21]. Therefore, using ICD-9-CM codes would only capture a small proportion of individuals with prediabetes. In addition, with lab results, a specific threshold of blood glucose for prediabetes can be identified, which is not the case with ICD-9-CM codes. Beneficiaries also need to meet the blood test requirement and the BMI criteria; however, BMI cannot be identified using ICD-9-CM codes. Therefore, we used NHANES to identify proportions of people with prediabetes eligible for MDPP by demographic characteristics and applied estimated proportions to the number of Medicare beneficiaries who met the other MDPP eligibility criteria (except for prediabetes status). A second limitation is that NHANES is a national representative data and not specifically designed for state or county level estimates. We adjusted our estimates for demographic characteristics to account for the variations of state or local demographics. Because of these and other predictors that can affect the estimates of prediabetes, it is hard to predict whether our estimates would over or underestimate the number of individuals eligible for MDPP across counties. We did not include other known predictors of prediabetes in our model, such as waist size, family history, lifestyle behavior (e.g., physical activities) [22], because these variables were not available in Medicare data. Thirdly, we calculated the eligible population from Part B beneficiaries only, which accounts for about 70 percent of total Medicare beneficiaries in 2015 [23] (due to the lack of availability of Part C encounter data). Finally, we used available Medicare 2015 data to estimate the MDPP eligible population, the number of eligible beneficiaries has likely increased since 2015 as the U.S. population continues to age [24].

## Conclusions

Millions of Medicare Part B beneficiaries are eligible for the MDPP. This study used geographic-based information to show that the number of MDPP-eligible Part B beneficiaries varied across counties, but that the majority live in urban areas. These estimates can be used to target eligible beneficiaries for program enrollment and identify opportunities to expand MDPP services in various communities.

## Supporting information

S1 File (PDF)
