# Supplementary material for: Estimated number of eligible Part B beneficiaries for the medicare diabetes prevention program at the county level and by urban–rural classification
Source: PLoS One. 2020 Nov 10;15(11):e0241757. doi: 10.1371/journal.pone.0241757 (PMC7654820; doi:10.1371/journal.pone.0241757)
Supplement: S1 File — (PDF) [file pone.0241757.s001.pdf]

S1 Calculation

Medicare Current Beneficiary Survey (MCBS) Descriptive Analysis: This study analyzed the 2015 MCBS Public Use File (n=12,311), a nationally representative, publicly accessible database from the Centers for Medicare & Medicaid Services. The authors conducted a descriptive analysis that accounted for the complex sample design with survey weights. The study found that 80% of Medicare beneficiaries indicated that they lived in a metro area, and 73% were still driving themselves to the doctor's office.

S1 Table. Estimated Number, Prevalence, and Density (MDPP-Eligible per Square Mile of Land Area) of MDPP-Eligible Part B Beneficiaries, by State

State	Estimated MDPP eligible	Prevalence	Land area (square miles)	Density (eligible beneficiaries per square mile of land area)
50 states +DC +PR+VI	5,271,473	28.5%	3,535,463.53	1.49
AK	13,361	28.4%	570,640.95	0.02
AL	94,797	28.5%	50,645.33	1.87
AR	69,287	28.6%	52,035.48	1.33
AZ	116,967	28.7%	113,594.08	1.03
CA	468,911	28.6%	155,779.22	3.01
CO	82,982	28.5%	103,641.89	0.80
CT	66,196	28.6%	4,842.36	13.67
DC	8,677	27.9%	61.05	142.13
DE	23,176	28.7%	1,948.54	11.89
FL	349,349	28.6%	53,624.76	6.51
GA	145,585	28.5%	57,513.49	2.53
HI	18,327	28.3%	6,422.63	2.85
IA	80,449	28.5%	55,857.13	1.44
ID	30,174	28.6%	82,643.12	0.37
IL	240,962	28.5%	55,518.93	4.34
IN	125,338	28.5%	35,826.11	3.50
KS	66,086	28.5%	81,758.72	0.81
KY	80,214	28.5%	39,486.34	2.03
LA	70,631	28.4%	43,203.90	1.63
MA	136,681	28.6%	7,800.06	17.52
MD	112,872	28.5%	9,707.24	11.63
ME	33,960	28.6%	30,842.92	1.10
MI	159,521	28.5%	56,538.90	2.82
MN	58,704	28.4%	79,626.74	0.74
MO	115,685	28.5%	68,741.52	1.68
MS	63,435	28.4%	46,923.27	1.35
MT	28,505	28.5%	145,545.80	0.20
NC	174,860	28.5%	48,617.91	3.60
ND	16,390	28.4%	69,000.80	0.24
NE	46,227	28.5%	76,824.17	0.60
NH	37,449	28.7%	8,952.65	4.18
NJ	155,111	28.6%	7,354.22	21.09
NM	36,493	28.5%	121,298.15	0.30
NV	44,124	28.6%	109,781.18	0.40
NY	240,431	28.5%	47,126.40	5.10
OH	172,428	28.5%	40,860.69	4.22
OK	81,203	28.6%	68,594.92	1.18
OR	66,042	28.6%	95,988.01	0.69
PA	208,600	28.5%	44,742.70	4.66
PR	7,601	28.9%	3,423.78	2.22
RI	16,030	28.6%	1,033.81	15.51
SC	108,225	28.5%	30,060.70	3.60
SD	21,538	28.5%	75,811.00	0.28
TN	115,013	28.5%	41,234.90	2.79
TX	355,280	28.6%	261,231.71	1.36
UT	35,516	28.6%	82,169.62	0.43
VA	164,566	28.5%	39,490.09	4.17
VI	2,530	27.2%	134.32	18.84
VT	19,523	28.6%	9,216.66	2.12
WA	130,898	28.6%	66,455.52	1.97
WI	100,047	28.5%	54,157.80	1.85
WV	38,286	28.5%	24,038.21	1.59
WY	16,231	28.5%	97,093.14	0.17

Counties with less than 100 MDPP-Eligible Part B Beneficiaries were excluded. * Proportions of beneficiaries enrolled in Part B (at least one month in Part B) per our study population of Medicare beneficiaries by county. Additionally, readers that are interested in MDPP eligible Part B beneficiaries per 1,000 can multiple the prevalence rates presented in S2 Table by 1,000.

S2 Table. Estimated Number, Prevalence, Density (MDPP-Eligible per Square Mile of Land Area) of MDPP-Eligible Part B Beneficiaries, by County

GEOID	State	County Name	Status	Estimated MDPP eligible	Prevalence	Land area (square miles)	Density (eligible beneficiaries per square mile of land area)	*Part B beneficiaries
02020	AK	Anchorage	urban	5,178	28.5%	1,704.68	3.038	82.6%
02050	AK	Bethel	rural	216	28.0%	40,570.00	0.005	81.7%
02060	AK	Bristol Bay	rural	114	27.9%	503.84	0.225	87.0%
02090	AK	Fairbanks North Star	urban	1,512	28.4%	7338.21	0.206	86.5%
02110	AK	Juneau	rural	795	28.5%	2,701.93	0.294	88.2%
02122	AK	Kenai Peninsula	rural	1,613	28.3%	16075.33	0.100	88.9%
02130	AK	Ketchikan Gateway	rural	378	28.1%	4858.41	0.078	88.0%
02150	AK	Kodiak Island	rural	153	28.3%	6549.58	0.023	81.4%
02170	AK	Matanuska-Susitna	urban	1,653	28.5%	24607.9	0.067	86.1%
02180	AK	Nome	rural	122	27.8%	22,961.76	0.005	74.8%
02220	AK	Sitka	rural	221	28.3%	2,870.34	0.077	86.1%
02240	AK	Southeast Fairbanks	rural	178	28.0%	24768.81	0.007	85.9%
02261	AK	Valdez-Cordova	rural	207	28.0%	34239.88	0.006	86.2%
02290	AK	Yukon-Koyukuk	rural	151	28.0%	145504.79	0.001	80.0%
01001	AL	Autauga	urban	880	28.8%	594.44	1.480	60.6%
01003	AL	Baldwin	urban	4,935	28.7%	1,589.78	3.104	59.4%
01005	AL	Barbour	rural	543	28.4%	884.88	0.613	73.1%
01007	AL	Bibb	urban	344	28.4%	622.58	0.552	53.8%
01009	AL	Blount	urban	777	28.5%	644.78	1.205	50.1%
01011	AL	Bullock	rural	113	27.6%	622.80	0.181	68.0%
01013	AL	Butler	rural	442	28.1%	776.83	0.568	80.2%
01015	AL	Calhoun	urban	2,848	28.5%	605.87	4.700	75.2%
01017	AL	Chambers	rural	827	28.5%	596.53	1.386	73.4%
01019	AL	Cherokee	rural	577	28.9%	553.70	1.041	68.6%
01021	AL	Chilton	urban	603	28.6%	692.85	0.870	48.7%
01023	AL	Choctaw	rural	372	28.1%	913.50	0.407	86.6%
01025	AL	Clarke	rural	613	28.2%	1,238.46	0.495	70.3%
01027	AL	Clay	rural	390	28.5%	603.96	0.646	78.2%
01029	AL	Cleburne	rural	353	28.4%	560.10	0.631	76.4%
01031	AL	Coffee	rural	1,178	28.6%	678.97	1.735	83.1%
01033	AL	Colbert	urban	1,412	28.7%	592.62	2.382	81.3%
01035	AL	Conecuh	rural	301	28.0%	850.16	0.355	79.3%
01037	AL	Coosa	rural	268	28.1%	650.93	0.412	78.0%
01039	AL	Covington	rural	1,094	28.5%	1,030.46	1.061	83.2%
01041	AL	Crenshaw	rural	334	28.3%	608.84	0.549	72.2%
01043	AL	Cullman	rural	1,963	28.6%	734.84	2.671	69.0%
01045	AL	Dale	rural	1,246	28.5%	561.15	2.221	80.4%
01047	AL	Dallas	rural	778	28.1%	978.69	0.795	67.1%
01049	AL	DeKalb	rural	1,499	28.5%	777.09	1.929	72.6%
01051	AL	Elmore	urban	1,628	28.6%	618.48	2.633	65.5%
01053	AL	Escambia	rural	812	28.4%	945.08	0.859	76.6%
01055	AL	Etowah	urban	2,324	28.6%	534.99	4.344	69.7%
01057	AL	Fayette	rural	427	28.6%	627.66	0.681	81.1%
01059	AL	Franklin	rural	834	28.7%	633.82	1.316	84.2%
01061	AL	Geneva	urban	770	28.4%	574.41	1.340	81.4%
01063	AL	Greene	rural	169	27.8%	647.11	0.262	82.6%
01065	AL	Hale	urban	407	27.8%	643.94	0.631	84.9%
01067	AL	Henry	urban	449	28.4%	561.75	0.799	69.4%
01069	AL	Houston	urban	2,359	28.6%	579.82	4.069	76.0%
01071	AL	Jackson	rural	1,391	28.7%	1,077.87	1.290	75.4%
01073	AL	Jefferson	urban	11,310	28.5%	1,111.28	10.178	48.0%
01075	AL	Lamar	rural	470	28.4%	604.85	0.777	86.2%

01077	AL	Lauderdale	urban	2,633	28.5%	667.70	3.944	80.5%
01079	AL	Lawrence	urban	590	28.5%	690.68	0.854	81.4%
01081	AL	Lee	urban	2,144	28.4%	607.54	3.529	75.2%
01083	AL	Limestone	urban	1,523	28.6%	559.94	2.719	73.8%
01085	AL	Lowndes	urban	152	27.6%	715.91	0.213	54.4%
01087	AL	Macon	rural	387	28.0%	608.89	0.636	61.3%
01089	AL	Madison	urban	7,220	28.5%	801.59	9.007	72.5%
01091	AL	Marengo	rural	406	28.3%	976.88	0.416	80.0%
01093	AL	Marion	rural	807	28.6%	742.29	1.088	83.9%
01095	AL	Marshall	rural	2,450	28.8%	565.84	4.330	77.1%
01097	AL	Mobile	urban	5,965	28.5%	1,229.44	4.852	47.2%
01099	AL	Monroe	rural	468	28.2%	1,025.67	0.456	76.9%
01101	AL	Montgomery	urban	3,903	28.4%	784.25	4.976	64.0%
01103	AL	Morgan	urban	2,962	28.6%	579.34	5.113	78.6%
01105	AL	Perry	rural	191	27.8%	719.66	0.266	83.0%
01107	AL	Pickens	urban	487	28.2%	881.41	0.553	84.7%
01109	AL	Pike	rural	643	28.4%	672.09	0.957	69.7%
01111	AL	Randolph	rural	555	28.6%	580.55	0.956	77.0%
01113	AL	Russell	urban	1,024	28.5%	641.14	1.597	67.7%
01117	AL	Shelby	urban	1,894	28.7%	784.93	2.414	48.4%
01115	AL	St. Clair	urban	1,149	28.6%	631.9	1.818	46.6%
01119	AL	Sumter	rural	268	27.9%	903.89	0.297	82.5%
01121	AL	Talladega	rural	1,564	28.5%	736.78	2.122	65.9%
01123	AL	Tallapoosa	rural	1,116	28.3%	716.52	1.557	79.7%
01125	AL	Tuscaloosa	urban	3,422	28.6%	1,321.75	2.589	78.9%
01127	AL	Walker	urban	1,456	28.7%	791.19	1.841	56.2%
01129	AL	Washington	rural	426	28.4%	1,080.21	0.395	79.7%
01131	AL	Wilcox	rural	245	27.6%	888.50	0.275	79.1%
01133	AL	Winston	rural	708	28.7%	612.98	1.155	79.2%
05001	AR	Arkansas	rural	569	28.4%	988.77	0.575	82.6%
05003	AR	Ashley	rural	649	28.6%	925.35	0.702	89.3%
05005	AR	Baxter	rural	1,774	28.9%	554.28	3.200	67.7%
05007	AR	Benton	urban	3,959	28.6%	847.36	4.673	59.1%
05009	AR	Boone	rural	1,179	28.6%	590.23	1.997	70.9%
05011	AR	Bradley	rural	314	28.5%	649.23	0.484	87.9%
05013	AR	Calhoun	rural	121	29.0%	628.58	0.193	82.2%
05015	AR	Carroll	rural	735	28.5%	630.09	1.167	62.2%
05017	AR	Chicot	rural	322	28.0%	644.30	0.500	83.3%
05019	AR	Clark	rural	615	28.5%	866.07	0.710	77.5%
05021	AR	Clay	rural	520	28.6%	639.46	0.813	81.4%
05023	AR	Cleburne	rural	1,052	28.8%	553.69	1.899	84.4%
05025	AR	Cleveland	urban	230	28.4%	597.78	0.385	84.7%
05027	AR	Columbia	rural	639	28.4%	766.05	0.835	84.3%
05029	AR	Conway	rural	621	28.6%	552.25	1.125	75.4%
05031	AR	Craighead	urban	2,196	28.8%	707.21	3.106	78.5%
05033	AR	Crawford	urban	1,037	28.6%	593.09	1.748	54.0%
05035	AR	Crittenden	urban	809	28.5%	609.76	1.326	73.6%
05037	AR	Cross	rural	446	28.6%	616.38	0.723	78.3%
05039	AR	Dallas	rural	192	28.2%	667.39	0.288	76.7%
05041	AR	Desha	rural	320	28.4%	768.15	0.416	84.4%
05043	AR	Drew	rural	429	28.4%	828.36	0.518	84.7%
05045	AR	Faulkner	urban	2,308	28.7%	647.88	3.562	81.5%
05047	AR	Franklin	rural	407	28.7%	608.86	0.668	64.8%
05049	AR	Fulton	rural	396	28.8%	618.19	0.640	72.3%
05051	AR	Garland	urban	4,270	28.8%	677.78	6.300	73.7%
05053	AR	Grant	urban	431	28.9%	631.81	0.682	79.9%
05055	AR	Greene	rural	967	28.7%	577.70	1.674	74.7%
05057	AR	Hempstead	rural	448	28.3%	727.52	0.616	75.1%
05059	AR	Hot Spring	rural	816	28.5%	615.2	1.326	77.8%
05061	AR	Howard	rural	390	28.8%	588.55	0.662	78.2%
05063	AR	Independence	rural	1,081	28.7%	763.95	1.416	86.6%
05065	AR	Izard	rural	474	28.8%	580.58	0.816	76.1%
05067	AR	Jackson	rural	432	28.3%	633.94	0.682	84.9%
05069	AR	Jefferson	urban	1,515	28.2%	870.75	1.740	69.8%
05071	AR	Johnson	rural	527	28.8%	659.80	0.799	67.7%
05073	AR	Lafayette	rural	191	27.9%	528.27	0.362	83.6%
05075	AR	Lawrence	rural	549	28.6%	587.61	0.935	79.1%
05077	AR	Lee	rural	182	27.8%	602.62	0.301	70.2%
05079	AR	Lincoln	urban	240	28.4%	561.52	0.427	78.1%
05081	AR	Little River	urban	347	28.4%	532.25	0.651	77.5%
05083	AR	Logan	rural	592	28.5%	708.12	0.836	69.5%
05085	AR	Lonoke	urban	1,383	28.8%	770.73	1.794	77.0%
05087	AR	Madison	urban	427	28.7%	834.26	0.511	57.9%
05089	AR	Marion	rural	514	28.7%	597.01	0.861	65.3%
05091	AR	Miller	urban	894	28.5%	625.58	1.429	72.9%
05093	AR	Mississippi	rural	770	28.6%	900.57	0.855	74.3%
05095	AR	Monroe	rural	206	28.3%	607.12	0.339	77.6%
05097	AR	Montgomery	rural	309	28.5%	779.88	0.396	77.1%
05099	AR	Nevada	rural	245	28.3%	617.84	0.397	75.3%
05101	AR	Newton	rural	242	28.6%	820.90	0.295	68.6%
05103	AR	Ouachita	rural	658	28.2%	732.78	0.898	70.8%
05105	AR	Perry	urban	317	28.4%	551.40	0.575	75.7%

05107	AR	Phillips	rural	426	28.0%	695.66	0.612	72.8%
05109	AR	Pike	rural	341	28.5%	600.62	0.568	80.6%
05111	AR	Poinsett	urban	610	28.7%	758.39	0.804	76.9%
05113	AR	Polk	rural	723	28.8%	857.68	0.843	80.8%
05115	AR	Pope	rural	1,359	28.6%	812.55	1.672	71.8%
05117	AR	Prairie	rural	267	28.6%	647.96	0.412	85.0%
05119	AR	Pulaski	urban	9,170	28.6%	759.76	12.070	72.1%
05121	AR	Randolph	rural	467	28.7%	652.19	0.717	71.7%
05125	AR	Saline	urban	1,338	28.8%	723.60	1.849	78.0%
05127	AR	Scott	rural	279	28.5%	892.32	0.313	66.0%
05129	AR	Searcy	rural	302	28.8%	666.09	0.453	72.9%
05131	AR	Sebastian	urban	2,341	28.6%	531.91	4.401	61.9%
05133	AR	Sevier	rural	334	28.6%	565.13	0.590	80.1%
05135	AR	Sharp	rural	715	28.8%	604.44	1.184	75.9%
05123	AR	St. Francis	rural	550	28.3%	634.77	0.866	72.5%
05137	AR	Stone	rural	515	28.8%	606.40	0.850	85.1%
05139	AR	Union	rural	1,176	28.2%	1,039.21	1.132	83.9%
05141	AR	Van Buren	rural	593	28.7%	708.14	0.837	74.1%
05143	AR	Washington	urban	3,706	28.7%	941.97	3.934	65.9%
05145	AR	White	rural	2,089	28.8%	1,035.08	2.018	79.1%
05147	AR	Woodruff	rural	231	28.3%	586.79	0.394	84.8%
05149	AR	Yell	rural	501	28.7%	929.98	0.538	74.5%
04001	AZ	Apache	rural	1,225	28.2%	11,197.52	0.109	76.8%
04003	AZ	Cochise	urban	3,304	28.6%	6,165.69	0.536	61.1%
04005	AZ	Coconino	urban	3,241	28.6%	18,618.89	0.174	77.3%
04007	AZ	Gila	rural	2,172	28.6%	4,757.93	0.457	78.7%
04009	AZ	Graham	rural	560	28.4%	4,622.60	0.121	60.8%
04011	AZ	Greenlee	rural	163	28.5%	1,843.13	0.088	77.7%
04012	AZ	La Paz	rural	671	28.6%	4499.63	0.149	74.5%
04013	AZ	Maricopa	urban	58,677	28.7%	9,200.14	6.378	48.9%
04015	AZ	Mohave	urban	6,764	28.6%	13,311.08	0.508	66.7%
04017	AZ	Navajo	rural	2,483	28.6%	9,950.42	0.249	70.8%
04019	AZ	Pima	urban	18,831	28.7%	9,187.04	2.050	46.8%
04021	AZ	Pinal	urban	5,609	28.7%	5,365.61	1.045	51.1%
04023	AZ	Santa Cruz	rural	786	28.6%	1236.92	0.636	42.4%
04025	AZ	Yavapai	urban	8,662	28.7%	8,123.50	1.066	65.7%
04027	AZ	Yuma	urban	3,818	28.8%	5,513.99	0.692	73.7%
06001	CA	Alameda	urban	16,633	28.4%	739.02	22.507	38.0%
06005	CA	Amador	rural	1,430	28.6%	594.58	2.405	69.5%
06007	CA	Butte	urban	7,184	28.7%	1,636.46	4.390	91.2%
06009	CA	Calaveras	rural	1,870	28.6%	1,020.01	1.833	77.7%
06011	CA	Colusa	rural	445	28.3%	1,150.73	0.386	81.8%
06013	CA	Contra Costa	urban	13,715	28.6%	715.94	19.156	36.4%
06015	CA	Del Norte	rural	764	28.5%	1006.37	0.759	83.7%
06017	CA	El Dorado	urban	4,353	28.6%	1707.88	2.549	54.9%
06019	CA	Fresno	urban	11,428	28.5%	5,957.99	1.918	53.2%
06021	CA	Glenn	rural	708	28.3%	1,313.95	0.539	91.0%
06023	CA	Humboldt	rural	4,072	28.6%	3,567.99	1.141	88.7%
06025	CA	Imperial	urban	3,030	28.3%	4,176.60	0.725	78.5%
06027	CA	Inyo	rural	525	28.5%	10,180.88	0.052	89.8%
06029	CA	Kern	urban	7,880	28.6%	8,131.92	0.969	45.3%
06031	CA	Kings	urban	1,714	28.6%	1,389.42	1.234	73.1%
06033	CA	Lake	rural	2,325	28.5%	1,256.46	1.850	85.8%
06035	CA	Lassen	rural	631	28.6%	4,541.18	0.139	86.5%
06037	CA	Los Angeles	urban	90,047	28.7%	4057.88	22.191	35.7%
06039	CA	Madera	urban	2,032	28.4%	2,137.07	0.951	50.0%
06041	CA	Marin	urban	6,092	28.7%	520.31	11.708	49.3%
06043	CA	Mariposa	rural	691	28.5%	1,448.82	0.477	86.1%
06045	CA	Mendocino	rural	3,259	28.6%	3,506.34	0.929	84.6%
06047	CA	Merced	urban	3,597	28.4%	1,934.97	1.859	78.7%
06049	CA	Modoc	rural	329	28.6%	3,917.77	0.084	84.4%
06051	CA	Mono	rural	508	28.5%	3,048.98	0.166	88.3%
06053	CA	Monterey	urban	8,457	28.5%	3,280.60	2.578	85.7%
06055	CA	Napa	urban	2,796	28.6%	748.36	3.736	49.0%
06057	CA	Nevada	rural	4,064	28.6%	957.77	4.243	78.2%
06059	CA	Orange	urban	34,847	28.6%	790.57	44.078	37.7%
06061	CA	Placer	urban	6,802	28.7%	1,407.01	4.834	41.9%
06063	CA	Plumas	rural	947	28.6%	2,553.04	0.371	85.4%
06065	CA	Riverside	urban	20,664	28.7%	7,206.48	2.867	31.6%
06067	CA	Sacramento	urban	16,067	28.5%	964.64	16.655	37.2%
06069	CA	San Benito	urban	1,045	28.7%	1388.71	0.752	80.8%
06071	CA	San Bernardino	urban	13,394	28.6%	20056.94	0.668	27.0%
06073	CA	San Diego	urban	37,900	28.6%	4206.63	9.010	39.0%
06075	CA	San Francisco	urban	11,820	28.4%	46.87	252.192	43.0%
06077	CA	San Joaquin	urban	8,142	28.4%	1391.32	5.852	47.1%
06079	CA	San Luis Obispo	urban	8,454	28.6%	3298.57	2.563	79.1%
06081	CA	San Mateo	urban	10,693	28.7%	448.41	23.846	42.3%
06083	CA	Santa Barbara	urban	9,452	28.5%	2735.09	3.456	72.7%
06085	CA	Santa Clara	urban	21,664	28.5%	1290.1	16.793	42.9%
06087	CA	Santa Cruz	urban	6,842	28.5%	445.17	15.370	79.6%
06089	CA	Shasta	urban	6,224	28.6%	3,775.40	1.649	88.2%
06091	CA	Sierra	rural	142	28.5%	953.21	0.149	88.6%

06093	CA	Siskiyou	rural	1,982	28.6%	6,277.89	0.316	84.5%
06095	CA	Solano	urban	5,356	28.4%	821.76	6.517	40.1%
06097	CA	Sonoma	urban	9,156	28.6%	1,575.85	5.810	44.9%
06099	CA	Stanislaus	urban	5,513	28.6%	1,494.83	3.688	39.9%
06101	CA	Sutter	urban	2,120	28.6%	602.41	3.519	88.0%
06103	CA	Tehama	rural	1,807	28.6%	2,949.71	0.612	91.4%
06105	CA	Trinity	rural	1,055	28.5%	3,179.25	0.332	87.5%
06107	CA	Tulare	urban	6,363	28.5%	4,824.21	1.319	75.0%
06109	CA	Tuolumne	rural	2,464	28.6%	2,220.88	1.110	88.4%
06111	CA	Ventura	urban	13,535	28.6%	1,843.13	7.344	53.5%
06113	CA	Yolo	urban	2,535	28.6%	1,014.69	2.498	45.0%
06115	CA	Yuba	urban	1,316	28.6%	631.84	2.082	80.5%
08001	CO	Adams	urban	3,428	28.5%	1,167.65	2.936	31.9%
08003	CO	Alamosa	rural	317	28.2%	722.64	0.439	66.7%
08005	CO	Arapahoe	urban	8,553	28.6%	798.10	10.717	42.2%
08007	CO	Archuleta	rural	538	28.3%	1,350.18	0.398	79.2%
08009	CO	Baca	rural	170	28.6%	2,554.97	0.067	94.3%
08011	CO	Bent	rural	122	28.6%	1,512.86	0.081	79.9%
08013	CO	Boulder	urban	5,280	28.5%	726.29	7.270	48.6%
08014	CO	Broomfield	urban	946	28.8%	33.03	28.648	38.0%
08015	CO	Chaffee	rural	778	28.7%	1,013.40	0.768	75.6%
08019	CO	Clear Creek	urban	121	28.2%	395.23	0.307	55.3%
08021	CO	Conejos	rural	156	27.6%	1,287.39	0.121	59.6%
08023	CO	Costilla	rural	123	28.9%	1,226.95	0.100	62.0%
08027	CO	Custer	rural	222	28.3%	738.63	0.301	73.1%
08029	CO	Delta	rural	1,204	28.5%	1,142.05	1.054	69.5%
08031	CO	Denver	urban	6,837	28.4%	153.00	44.686	39.3%
08035	CO	Douglas	urban	2,440	28.6%	840.25	2.904	48.5%
08037	CO	Eagle	rural	884	28.5%	1,684.53	0.525	83.1%
08041	CO	El Paso	urban	10,455	28.5%	2126.8	4.916	59.1%
08039	CO	Elbert	urban	502	28.3%	1,850.85	0.271	58.1%
08043	CO	Fremont	rural	1,267	28.5%	1,533.07	0.826	59.9%
08045	CO	Garfield	rural	1,323	28.6%	2,947.56	0.449	80.2%
08049	CO	Grand	rural	374	28.6%	1,846.33	0.203	76.9%
08051	CO	Gunnison	rural	375	28.6%	3,239.10	0.116	88.1%
08055	CO	Huerfano	rural	288	28.4%	1,591.00	0.181	75.8%
08059	CO	Jefferson	urban	6,757	28.5%	764.21	8.842	32.5%
08063	CO	Kit Carson	rural	250	28.7%	2160.82	0.116	90.7%
08067	CO	La Plata	rural	1,569	28.6%	1692.08	0.927	81.1%
08065	CO	Lake	rural	136	28.8%	376.91	0.360	81.2%
08069	CO	Larimer	urban	6,804	28.6%	2,596.00	2.621	61.9%
08071	CO	Las Animas	rural	470	28.3%	4772.67	0.098	73.5%
08073	CO	Lincoln	rural	157	28.5%	2,577.63	0.061	89.5%
08075	CO	Logan	rural	565	28.5%	1,838.55	0.307	81.6%
08077	CO	Mesa	urban	3,288	28.6%	3,328.97	0.988	55.4%
08081	CO	Moffat	rural	311	28.7%	4,743.29	0.066	81.7%
08083	CO	Montezuma	rural	911	28.5%	2,029.53	0.449	79.7%
08085	CO	Montrose	rural	1,423	28.7%	2,240.70	0.635	71.4%
08087	CO	Morgan	rural	696	28.5%	1,280.43	0.543	82.4%
08089	CO	Otero	rural	549	28.3%	1,261.96	0.435	73.9%
08091	CO	Ouray	rural	192	28.7%	541.59	0.354	80.1%
08093	CO	Park	urban	357	28.3%	2,193.85	0.163	61.2%
08095	CO	Phillips	rural	168	28.4%	687.93	0.244	89.4%
08097	CO	Pitkin	rural	488	28.6%	970.70	0.503	88.1%
08099	CO	Prowers	rural	350	28.7%	1,638.39	0.214	89.2%
08101	CO	Pueblo	urban	3,138	28.6%	2,386.10	1.315	53.4%
08103	CO	Rio Blanco	rural	147	28.3%	3220.93	0.046	76.3%
08105	CO	Rio Grande	rural	354	28.7%	911.96	0.388	72.7%
08107	CO	Routt	rural	600	28.5%	2,362.03	0.254	84.6%
08109	CO	Saguache	rural	187	28.5%	3,168.52	0.059	72.2%
08113	CO	San Miguel	rural	156	28.2%	1286.61	0.122	79.7%
08115	CO	Sedgwick	rural	107	28.5%	548.04	0.196	88.5%
08117	CO	Summit	rural	574	28.4%	608.36	0.943	81.2%
08119	CO	Teller	urban	605	28.4%	557.06	1.086	55.8%
08121	CO	Washington	rural	161	28.6%	2,518.03	0.064	89.0%
08123	CO	Weld	urban	3,973	28.5%	3,987.24	0.996	58.7%
08125	CO	Yuma	rural	327	28.4%	2,364.40	0.138	91.5%
09001	CT	Fairfield	urban	16,894	28.6%	624.89	27.035	61.8%
09003	CT	Hartford	urban	15,877	28.5%	735.10	21.599	55.4%
09005	CT	Litchfield	rural	4,814	28.6%	920.56	5.229	65.1%
09007	CT	Middlesex	urban	3,724	28.6%	369.30	10.084	59.4%
09009	CT	New Haven	urban	14,128	28.5%	604.51	23.371	53.0%
09011	CT	New London	urban	5,983	28.6%	664.88	8.999	66.5%
09013	CT	Tolland	urban	2,519	28.7%	410.21	6.141	57.1%
09015	CT	Windham	urban	2,257	28.6%	512.91	4.401	62.4%
11001	DC	District of Columbia	urban	8,677	27.9%	61.05	142.125	63.2%
10001	DE	Kent	urban	3,715	28.5%	586.18	6.337	83.9%
10003	DE	New Castle	urban	10,650	28.6%	426.29	24.984	78.0%
10005	DE	Sussex	urban	8,810	28.8%	936.08	9.412	87.5%
12001	FL	Alachua	urban	4,807	28.5%	875.02	5.494	66.5%
12003	FL	Baker	urban	344	28.7%	585.23	0.587	60.0%
12005	FL	Bay	urban	4,113	28.6%	758.46	5.423	71.6%

12007	FL	Bradford	rural	448	28.5%	293.96	1.524	67.2%
12009	FL	Brevard	urban	13,014	28.6%	1,015.66	12.814	52.3%
12011	FL	Broward	urban	17,971	28.5%	1,209.79	14.855	32.1%
12013	FL	Calhoun	rural	244	28.6%	567.33	0.431	63.4%
12015	FL	Charlotte	urban	6,041	28.7%	680.28	8.880	60.6%
12017	FL	Citrus	urban	5,024	28.8%	581.70	8.637	60.3%
12019	FL	Clay	urban	3,457	28.6%	604.36	5.719	66.8%
12021	FL	Collier	urban	12,187	28.8%	1,998.32	6.099	71.7%
12023	FL	Columbia	rural	1,490	28.5%	797.57	1.868	66.8%
12027	FL	DeSoto	rural	669	28.6%	637.06	1.050	66.7%
12029	FL	Dixie	rural	382	28.4%	705.05	0.541	65.5%
12031	FL	Duval	urban	13,468	28.5%	762.19	17.670	57.6%
12033	FL	Escambia	urban	6,252	28.4%	656.46	9.525	62.3%
12035	FL	Flagler	urban	2,842	28.6%	485.46	5.854	50.2%
12037	FL	Franklin	rural	290	28.3%	534.72	0.543	64.4%
12039	FL	Gadsden	urban	487	28.0%	516.33	0.942	33.2%
12041	FL	Gilchrist	urban	384	28.6%	349.68	1.099	66.8%
12043	FL	Glades	rural	137	28.3%	806.01	0.170	61.9%
12045	FL	Gulf	urban	427	28.6%	564.01	0.757	73.7%
12047	FL	Hamilton	rural	282	28.0%	513.79	0.549	68.6%
12049	FL	Hardee	rural	374	28.5%	637.78	0.587	64.9%
12051	FL	Hendry	rural	536	28.1%	1,152.75	0.465	62.7%
12053	FL	Hernando	urban	3,597	28.6%	472.54	7.611	37.3%
12055	FL	Highlands	urban	3,266	28.8%	1,016.61	3.213	62.0%
12057	FL	Hillsborough	urban	14,864	28.5%	1,020.21	14.570	40.6%
12059	FL	Holmes	rural	474	28.7%	478.78	0.991	75.1%
12061	FL	Indian River	urban	5,974	28.7%	502.87	11.880	70.4%
12063	FL	Jackson	rural	1,141	28.5%	917.76	1.244	72.2%
12065	FL	Jefferson	urban	231	28.1%	598.10	0.386	40.3%
12067	FL	Lafayette	rural	112	28.7%	543.41	0.206	74.3%
12069	FL	Lake	urban	15,067	28.9%	938.38	16.056	60.4%
12071	FL	Lee	urban	18,585	28.7%	784.51	23.690	59.6%
12073	FL	Leon	urban	2,686	28.3%	666.85	4.028	36.6%
12075	FL	Levy	rural	1,020	28.6%	1,118.21	0.912	63.8%
12079	FL	Madison	rural	395	28.4%	695.95	0.568	64.5%
12081	FL	Manatee	urban	8,151	28.6%	742.93	10.972	56.1%
12083	FL	Marion	urban	8,975	28.7%	1,584.55	5.664	51.0%
12085	FL	Martin	urban	5,687	28.8%	543.46	10.464	67.3%
12086	FL	Miami-Dade	urban	19,525	28.7%	1897.72	10.288	23.8%
12087	FL	Monroe	rural	2,416	28.3%	983.28	2.457	78.9%
12089	FL	Nassau	urban	2,099	28.7%	648.64	3.235	65.3%
12091	FL	Okaloosa	urban	4,824	28.7%	930.25	5.186	78.4%
12093	FL	Okeechobee	rural	761	28.7%	768.91	0.990	54.3%
12095	FL	Orange	urban	12,023	28.5%	903.43	13.308	43.6%
12097	FL	Osceola	urban	3,172	28.6%	1,327.45	2.389	35.7%
12099	FL	Palm Beach	urban	26,652	28.7%	1969.76	13.530	49.3%
12101	FL	Pasco	urban	7,386	28.7%	746.89	9.889	36.4%
12103	FL	Pinellas	urban	17,727	28.5%	273.80	64.746	41.9%
12105	FL	Polk	urban	9,232	28.6%	1,797.84	5.135	43.3%
12107	FL	Putnam	rural	1,607	28.7%	727.62	2.209	61.5%
12113	FL	Santa Rosa	urban	3,193	28.7%	1011.6	3.156	62.8%
12115	FL	Sarasota	urban	18,349	28.8%	555.87	33.009	66.8%
12117	FL	Seminole	urban	5,519	28.6%	309.22	17.848	48.1%
12109	FL	St. Johns	urban	6,172	28.7%	600.66	10.275	67.0%
12111	FL	St. Lucie	urban	5,910	28.6%	571.93	10.333	52.0%
12119	FL	Sumter	urban	2,148	28.6%	546.93	3.927	56.8%
12121	FL	Suwannee	rural	1,089	28.6%	688.55	1.582	69.6%
12123	FL	Taylor	rural	463	28.5%	1,043.31	0.444	70.1%
12125	FL	Union	rural	185	28.5%	243.56	0.761	67.3%
12127	FL	Volusia	urban	10,711	28.6%	1,101.03	9.728	43.3%
12129	FL	Wakulla	urban	370	28.4%	606.42	0.610	33.7%
12131	FL	Walton	urban	1,310	28.6%	1,037.62	1.262	71.3%
12133	FL	Washington	rural	524	28.7%	582.80	0.899	75.5%
13001	GA	Appling	rural	334	28.5%	507.08	0.658	66.3%
13003	GA	Atkinson	rural	130	28.3%	339.38	0.384	63.9%
13005	GA	Bacon	rural	165	28.4%	258.58	0.637	64.4%
13009	GA	Baldwin	rural	571	28.6%	257.84	2.215	47.7%
13011	GA	Banks	rural	231	28.9%	232.09	0.995	62.3%
13013	GA	Barrow	urban	1,038	28.6%	160.31	6.474	56.2%
13015	GA	Bartow	urban	1,564	28.6%	459.54	3.402	61.5%
13017	GA	Ben Hill	rural	342	28.4%	250.12	1.366	64.2%
13019	GA	Berrien	rural	346	28.6%	451.90	0.767	64.5%
13021	GA	Bibb	urban	2,752	28.3%	249.76	11.020	58.6%
13023	GA	Bleckley	rural	103	28.3%	215.87	0.475	77.0%
13025	GA	Brantley	urban	259	28.4%	442.36	0.585	65.6%
13027	GA	Brooks	urban	215	28.2%	493.05	0.436	63.3%
13029	GA	Bryan	urban	491	28.6%	435.97	1.127	60.1%
13031	GA	Bulloch	rural	877	28.6%	672.81	1.304	65.8%
13033	GA	Burke	urban	303	28.5%	826.97	0.367	55.1%
13035	GA	Butts	urban	400	28.3%	184.39	2.167	53.3%
13037	GA	Calhoun	rural	146	28.3%	280.37	0.522	62.4%
13039	GA	Camden	rural	798	28.6%	613.03	1.301	69.9%

13043	GA	Candler	rural	167	28.0%	243.04	0.687	59.1%
13045	GA	Carroll	urban	1,932	28.6%	499.08	3.872	58.4%
13047	GA	Catoosa	urban	934	28.6%	162.16	5.757	61.1%
13049	GA	Charlton	rural	170	28.6%	773.58	0.220	69.5%
13051	GA	Chatham	urban	4,571	28.4%	426.44	10.719	58.8%
13055	GA	Chattooga	rural	517	28.6%	313.34	1.649	61.1%
13057	GA	Cherokee	urban	2,100	28.8%	421.67	4.980	56.0%
13059	GA	Clarke	urban	1,802	28.5%	119.20	15.119	65.0%
13063	GA	Clayton	urban	2,859	28.5%	141.57	20.196	46.1%
13065	GA	Clinch	rural	114	28.0%	800.22	0.142	64.3%
13067	GA	Cobb	urban	11,991	28.6%	339.55	35.313	54.7%
13069	GA	Coffee	rural	594	28.3%	575.10	1.034	64.0%
13071	GA	Colquitt	rural	788	28.6%	544.15	1.449	66.0%
13073	GA	Columbia	urban	1,825	28.5%	290.09	6.292	63.1%
13075	GA	Cook	rural	312	28.5%	227.16	1.375	66.5%
13077	GA	Coweta	urban	1,958	28.6%	440.89	4.440	54.9%
13081	GA	Crisp	rural	372	28.2%	272.58	1.363	59.9%
13083	GA	Dade	urban	381	28.8%	173.98	2.191	61.6%
13085	GA	Dawson	urban	536	28.7%	210.83	2.544	61.6%
13089	GA	DeKalb	urban	7,249	28.4%	267.58	27.091	47.3%
13087	GA	Decatur	rural	518	28.3%	597.14	0.867	67.1%
13091	GA	Dodge	rural	460	28.6%	495.89	0.928	64.9%
13093	GA	Dooly	rural	193	28.2%	391.94	0.492	61.8%
13095	GA	Dougherty	urban	1,727	28.3%	328.69	5.254	64.9%
13097	GA	Douglas	urban	1,480	28.8%	200.07	7.396	52.3%
13099	GA	Early	rural	222	28.3%	512.59	0.433	63.2%
13103	GA	Effingham	urban	701	28.6%	477.70	1.467	62.8%
13105	GA	Elbert	rural	481	28.4%	351.06	1.369	64.3%
13107	GA	Emanuel	rural	398	28.4%	680.60	0.585	58.9%
13109	GA	Evans	rural	190	28.3%	182.85	1.039	60.6%
13111	GA	Fannin	rural	833	28.7%	386.72	2.154	70.7%
13113	GA	Fayette	urban	2,346	28.5%	194.34	12.074	59.6%
13115	GA	Floyd	urban	1,944	28.5%	509.91	3.812	67.1%
13117	GA	Forsyth	urban	2,189	28.9%	224.02	9.772	55.7%
13119	GA	Franklin	rural	573	28.5%	261.50	2.193	66.6%
13121	GA	Fulton	urban	10,608	28.3%	526.63	20.143	52.1%
13123	GA	Gilmer	rural	830	28.6%	426.54	1.945	67.6%
13127	GA	Glynn	urban	2,093	28.6%	419.75	4.986	68.8%
13129	GA	Gordon	rural	1,011	28.6%	355.81	2.842	68.6%
13131	GA	Grady	rural	418	28.3%	454.53	0.920	62.3%
13133	GA	Greene	rural	621	28.4%	387.44	1.603	68.9%
13135	GA	Gwinnett	urban	7,738	28.6%	430.38	17.979	50.1%
13137	GA	Habersham	rural	996	28.6%	276.74	3.600	62.0%
13139	GA	Hall	urban	3,286	28.6%	392.78	8.366	61.1%
13141	GA	Hancock	rural	129	27.8%	471.84	0.273	48.9%
13143	GA	Haralson	urban	527	28.5%	282.17	1.868	59.7%
13145	GA	Harris	urban	483	28.4%	463.87	1.041	60.3%
13147	GA	Hart	rural	533	28.3%	232.39	2.295	69.6%
13149	GA	Heard	urban	140	28.7%	296.03	0.474	56.7%
13151	GA	Henry	urban	2,326	28.6%	322.13	7.222	50.7%
13153	GA	Houston	urban	2,212	28.6%	375.54	5.891	73.4%
13155	GA	Irwin	rural	141	28.5%	354.34	0.398	66.5%
13157	GA	Jackson	rural	1,580	28.8%	339.66	4.652	61.2%
13159	GA	Jasper	urban	195	28.4%	368.16	0.531	55.6%
13161	GA	Jeff Davis	rural	253	28.7%	330.74	0.765	69.6%
13163	GA	Jefferson	rural	290	28.1%	526.48	0.551	60.2%
13165	GA	Jenkins	rural	135	28.1%	347.28	0.389	56.5%
13167	GA	Johnson	rural	138	28.4%	303.01	0.456	60.5%
13169	GA	Jones	urban	253	28.2%	393.93	0.643	53.4%
13171	GA	Lamar	urban	301	28.5%	183.50	1.639	54.7%
13173	GA	Lanier	urban	104	28.4%	185.26	0.560	66.7%
13175	GA	Laurens	rural	872	28.4%	807.30	1.080	61.0%
13177	GA	Lee	urban	388	28.7%	355.78	1.091	65.7%
13179	GA	Liberty	urban	560	28.8%	489.80	1.144	68.0%
13181	GA	Lincoln	urban	174	28.0%	210.38	0.829	62.5%
13185	GA	Lowndes	urban	1,670	28.6%	496.07	3.366	67.9%
13187	GA	Lumpkin	rural	564	28.7%	282.93	1.993	65.6%
13193	GA	Macon	rural	163	28.1%	400.64	0.407	54.1%
13195	GA	Madison	urban	535	28.6%	282.31	1.896	60.7%
13189	GA	McDuffie	urban	335	28.2%	257.46	1.303	51.4%
13191	GA	McIntosh	urban	262	28.8%	424.30	0.617	61.1%
13199	GA	Meriwether	urban	321	28.0%	501.22	0.641	51.4%
13201	GA	Miller	rural	139	28.5%	282.42	0.493	68.7%
13205	GA	Mitchell	rural	373	28.4%	512.08	0.729	61.2%
13207	GA	Monroe	urban	381	28.4%	395.66	0.962	62.5%
13209	GA	Montgomery	rural	147	28.4%	239.52	0.614	61.1%
13211	GA	Morgan	urban	385	28.7%	347.35	1.108	58.3%
13213	GA	Murray	urban	655	28.7%	344.47	1.903	73.1%
13215	GA	Muscogee	urban	2,988	28.3%	216.39	13.809	62.6%
13217	GA	Newton	urban	1,352	28.5%	272.16	4.969	51.6%
13219	GA	Oconee	urban	654	28.6%	184.29	3.550	62.9%
13221	GA	Oglethorpe	urban	178	28.3%	439.01	0.406	64.9%

13223	GA	Paulding	urban	1,163	28.4%	312.22	3.724	54.6%
13225	GA	Peach	urban	569	28.6%	150.27	3.788	63.0%
13227	GA	Pickens	urban	956	28.7%	232.06	4.121	63.9%
13229	GA	Pierce	rural	400	28.5%	316.49	1.265	68.1%
13231	GA	Pike	urban	300	28.4%	216.09	1.388	55.6%
13233	GA	Polk	rural	828	28.4%	310.33	2.668	63.5%
13235	GA	Pulaski	urban	227	28.4%	249.03	0.910	71.3%
13237	GA	Putnam	rural	531	28.4%	344.64	1.539	64.4%
13241	GA	Rabun	rural	589	28.7%	369.99	1.591	72.1%
13243	GA	Randolph	rural	140	28.1%	428.24	0.326	59.7%
13245	GA	Richmond	urban	3,237	28.3%	324.33	9.980	57.0%
13247	GA	Rockdale	urban	1,054	28.7%	129.79	8.124	48.9%
13251	GA	Screven	rural	248	28.1%	645.10	0.385	59.3%
13253	GA	Seminole	rural	204	28.5%	235.23	0.868	64.2%
13255	GA	Spalding	urban	1,159	28.5%	196.47	5.901	56.5%
13257	GA	Stephens	rural	719	28.6%	179.13	4.013	65.6%
13261	GA	Sumter	rural	526	28.4%	482.70	1.089	58.7%
13263	GA	Talbot	rural	111	28.4%	391.39	0.284	54.2%
13267	GA	Tattnall	rural	326	28.3%	479.40	0.681	59.3%
13269	GA	Taylor	rural	147	28.4%	376.69	0.391	57.1%
13271	GA	Telfair	rural	223	28.6%	437.30	0.510	63.4%
13273	GA	Terrell	urban	184	28.3%	335.44	0.549	57.9%
13275	GA	Thomas	rural	1,075	28.3%	544.60	1.974	64.0%
13277	GA	Tift	rural	739	28.4%	258.91	2.855	63.7%
13279	GA	Toombs	rural	561	28.4%	364.00	1.542	67.0%
13281	GA	Towns	rural	539	28.8%	166.56	3.236	67.1%
13285	GA	Troup	rural	1,117	28.2%	413.99	2.697	60.5%
13287	GA	Turner	rural	168	28.7%	285.39	0.589	57.7%
13289	GA	Twiggs	urban	122	28.2%	358.40	0.339	55.1%
13291	GA	Union	rural	899	28.7%	321.93	2.794	68.2%
13293	GA	Upson	rural	491	28.2%	323.44	1.518	55.5%
13295	GA	Walker	urban	1,453	28.7%	446.38	3.254	59.7%
13297	GA	Walton	urban	1,663	28.5%	325.68	5.105	54.7%
13299	GA	Ware	rural	763	28.3%	892.46	0.855	67.3%
13301	GA	Warren	rural	104	28.5%	284.30	0.364	57.3%
13303	GA	Washington	rural	300	28.0%	678.45	0.443	54.3%
13305	GA	Wayne	rural	516	28.8%	641.78	0.805	63.8%
13311	GA	White	rural	675	28.9%	240.69	2.803	62.1%
13313	GA	Whitfield	urban	1,827	28.6%	290.46	6.289	72.4%
13315	GA	Wilcox	rural	159	28.4%	377.70	0.421	60.6%
13317	GA	Wilkes	rural	247	28.1%	469.49	0.526	62.6%
13319	GA	Wilkinson	rural	166	28.2%	447.31	0.372	55.0%
13321	GA	Worth	urban	364	28.5%	570.70	0.638	67.5%
15001	HI	Hawaii	rural	3,392	28.4%	4,028.42	0.842	46.3%
15003	HI	Honolulu	urban	11,678	28.2%	600.74	19.439	34.8%
15007	HI	Kauai	rural	1,298	28.3%	619.96	2.093	47.9%
15009	HI	Maui	urban	1,960	28.4%	1,161.52	1.687	36.1%
19001	IA	Adair	rural	236	29.0%	569.27	0.414	85.0%
19003	IA	Adams	rural	140	28.5%	423.44	0.331	89.6%
19005	IA	Allamakee	rural	538	28.4%	639.08	0.842	83.2%
19007	IA	Appanoose	rural	430	28.5%	497.29	0.864	79.3%
19009	IA	Audubon	rural	251	28.5%	442.96	0.566	90.5%
19011	IA	Benton	urban	638	28.6%	716.26	0.891	72.5%
19013	IA	Black Hawk	urban	3,217	28.4%	565.77	5.687	77.4%
19015	IA	Boone	rural	711	28.5%	571.57	1.244	79.3%
19017	IA	Bremer	urban	873	28.7%	435.48	2.004	85.3%
19019	IA	Buchanan	rural	597	28.6%	571.02	1.046	79.4%
19021	IA	Buena Vista	rural	540	28.5%	574.91	0.939	87.3%
19023	IA	Butler	rural	573	28.4%	580.13	0.988	84.1%
19025	IA	Calhoun	rural	401	28.6%	569.97	0.704	90.3%
19027	IA	Carroll	rural	760	28.5%	569.44	1.334	85.7%
19029	IA	Cass	rural	522	28.7%	564.27	0.924	84.5%
19031	IA	Cedar	rural	478	28.7%	579.44	0.824	72.2%
19033	IA	Cerro Gordo	rural	1,630	28.5%	568.31	2.868	89.9%
19035	IA	Cherokee	rural	434	28.5%	576.91	0.752	83.0%
19037	IA	Chickasaw	rural	422	28.6%	504.38	0.837	91.3%
19039	IA	Clarke	rural	272	28.7%	431.17	0.631	82.4%
19041	IA	Clay	rural	651	28.4%	567.24	1.147	94.0%
19043	IA	Clayton	rural	622	28.4%	778.54	0.799	75.9%
19045	IA	Clinton	rural	1,319	28.4%	694.91	1.898	76.5%
19047	IA	Crawford	rural	506	28.5%	714.19	0.708	85.3%
19049	IA	Dallas	urban	1,024	28.6%	588.45	1.741	72.6%
19051	IA	Davis	rural	257	28.7%	502.19	0.511	85.5%
19053	IA	Decatur	rural	264	28.7%	531.88	0.496	83.0%
19055	IA	Delaware	rural	436	28.6%	577.76	0.755	74.9%
19057	IA	Des Moines	rural	1,433	28.5%	416.12	3.445	86.4%
19059	IA	Dickinson	rural	805	28.7%	380.61	2.115	90.4%
19061	IA	Dubuque	urban	1,725	28.5%	608.30	2.836	47.7%
19063	IA	Emmet	rural	361	28.5%	395.88	0.913	91.0%
19065	IA	Fayette	rural	678	28.4%	730.81	0.928	83.6%
19067	IA	Floyd	rural	575	28.5%	500.63	1.148	88.6%
19069	IA	Franklin	rural	336	28.2%	581.97	0.578	92.2%

19071	IA	Fremont	rural	237	28.4%	511.15	0.463	84.3%
19073	IA	Greene	rural	324	28.4%	569.57	0.568	80.5%
19075	IA	Grundy	urban	399	28.5%	501.86	0.795	83.6%
19077	IA	Guthrie	urban	401	28.7%	590.62	0.680	76.6%
19079	IA	Hamilton	rural	491	28.4%	576.75	0.851	83.4%
19081	IA	Hancock	rural	394	28.4%	571.00	0.690	91.6%
19083	IA	Hardin	rural	682	28.6%	569.31	1.199	86.8%
19085	IA	Harrison	urban	478	28.5%	696.85	0.687	83.9%
19087	IA	Henry	rural	657	28.6%	434.33	1.513	84.5%
19089	IA	Howard	rural	381	28.4%	473.25	0.805	91.9%
19091	IA	Humboldt	rural	341	28.4%	434.35	0.785	88.4%
19093	IA	Ida	rural	267	28.5%	431.51	0.620	83.9%
19095	IA	Iowa	rural	443	28.3%	586.46	0.756	74.1%
19097	IA	Jackson	rural	550	28.6%	636.04	0.865	67.8%
19099	IA	Jasper	rural	1,037	28.6%	730.42	1.420	80.9%
19101	IA	Jefferson	rural	579	28.5%	435.51	1.330	80.6%
19103	IA	Johnson	urban	2,286	28.5%	614.04	3.722	70.7%
19105	IA	Jones	urban	533	28.4%	575.62	0.926	71.0%
19107	IA	Keokuk	rural	362	28.6%	579.18	0.626	75.3%
19109	IA	Kossuth	rural	609	28.5%	972.72	0.626	92.4%
19111	IA	Lee	rural	1,137	28.6%	517.52	2.197	84.1%
19113	IA	Linn	urban	4,368	28.6%	716.88	6.092	60.9%
19115	IA	Louisa	rural	293	28.5%	401.77	0.729	77.2%
19117	IA	Lucas	rural	278	28.4%	430.59	0.645	77.7%
19119	IA	Lyon	rural	376	28.2%	587.65	0.639	87.2%
19121	IA	Madison	urban	401	28.5%	561.01	0.715	74.4%
19123	IA	Mahaska	rural	620	28.4%	570.86	1.087	80.3%
19125	IA	Marion	rural	1,010	28.5%	554.53	1.821	83.8%
19127	IA	Marshall	rural	1,202	28.6%	572.50	2.100	79.7%
19129	IA	Mills	urban	369	28.2%	437.44	0.845	77.5%
19131	IA	Mitchell	rural	485	28.4%	469.13	1.034	94.2%
19133	IA	Monona	rural	311	28.4%	694.07	0.448	76.2%
19135	IA	Monroe	rural	249	28.4%	433.72	0.574	83.1%
19137	IA	Montgomery	rural	366	28.3%	424.10	0.863	86.6%
19139	IA	Muscatine	rural	958	28.5%	437.47	2.191	67.8%
19141	IA	O'Brien	rural	570	28.3%	573.04	0.995	90.4%
19143	IA	Osceola	rural	216	28.5%	398.68	0.541	87.6%
19145	IA	Page	rural	514	28.3%	534.94	0.962	86.8%
19147	IA	Palo Alto	rural	375	28.4%	563.84	0.664	92.0%
19149	IA	Plymouth	urban	658	28.4%	862.89	0.762	77.4%
19151	IA	Pocahontas	rural	315	28.7%	577.24	0.547	89.3%
19153	IA	Polk	urban	9,146	28.6%	573.79	15.940	69.6%
19155	IA	Pottawattamie	urban	2,055	28.5%	950.28	2.163	66.8%
19157	IA	Poweshiek	rural	627	28.7%	584.93	1.072	80.7%
19159	IA	Ringgold	rural	193	28.5%	535.50	0.360	87.3%
19161	IA	Sac	rural	424	28.3%	575.01	0.738	90.3%
19163	IA	Scott	urban	3,754	28.5%	458.09	8.194	67.2%
19165	IA	Shelby	rural	426	28.5%	590.78	0.722	86.3%
19167	IA	Sioux	rural	971	28.6%	768.33	1.263	87.7%
19169	IA	Story	urban	1,849	28.6%	572.82	3.227	76.4%
19171	IA	Tama	rural	553	28.5%	721.01	0.767	79.6%
19173	IA	Taylor	rural	255	28.5%	531.90	0.480	93.7%
19175	IA	Union	rural	488	28.6%	423.65	1.152	84.7%
19177	IA	Van Buren	rural	289	28.5%	484.79	0.596	81.6%
19179	IA	Wapello	rural	1,045	28.6%	431.83	2.420	83.3%
19181	IA	Warren	urban	1,130	28.5%	569.83	1.983	72.8%
19183	IA	Washington	urban	704	28.5%	568.84	1.237	78.3%
19185	IA	Wayne	rural	215	28.8%	525.44	0.409	79.5%
19187	IA	Webster	rural	1,195	28.5%	715.62	1.669	84.3%
19189	IA	Winnebago	rural	428	28.6%	400.49	1.070	88.4%
19191	IA	Winneshiek	rural	677	28.4%	689.87	0.982	77.6%
19193	IA	Woodbury	urban	2,087	28.5%	872.83	2.391	65.9%
19195	IA	Worth	rural	256	28.5%	400.12	0.639	85.8%
19197	IA	Wright	rural	506	28.4%	580.42	0.871	90.9%
16001	ID	Ada	urban	5,658	28.5%	1,052.58	5.375	41.5%
16003	ID	Adams	rural	166	28.4%	1,363.06	0.122	74.1%
16005	ID	Bannock	urban	1,332	28.5%	1,111.99	1.198	56.3%
16007	ID	Bear Lake	rural	216	28.4%	974.78	0.222	89.4%
16009	ID	Benewah	rural	313	28.6%	776.62	0.403	68.0%
16011	ID	Bingham	rural	711	28.5%	2,093.98	0.340	64.8%
16013	ID	Blaine	rural	650	28.8%	2,643.59	0.246	69.0%
16015	ID	Boise	urban	118	27.8%	1,899.24	0.062	42.8%
16017	ID	Bonner	rural	1,236	28.5%	1,734.57	0.712	60.9%
16019	ID	Bonneville	urban	1,853	28.7%	1,866.08	0.993	66.6%
16021	ID	Boundary	rural	331	28.4%	1,268.56	0.261	59.4%
16027	ID	Canyon	urban	2,287	28.5%	587.37	3.893	37.5%
16029	ID	Caribou	rural	189	28.8%	1,764.15	0.107	76.6%
16031	ID	Cassia	rural	446	28.5%	2,565.08	0.174	68.6%
16035	ID	Clearwater	rural	371	28.3%	2,457.27	0.151	88.6%
16037	ID	Custer	rural	188	28.4%	4,920.94	0.038	87.9%
16039	ID	Elmore	rural	444	28.6%	3,074.74	0.144	72.2%
16041	ID	Franklin	urban	314	28.5%	663.64	0.473	81.6%

16043	ID	Fremont	rural	290	28.5%	1,863.53	0.156	73.6%
16045	ID	Gem	urban	348	28.5%	560.90	0.620	43.5%
16047	ID	Gooding	rural	346	28.9%	728.97	0.475	66.8%
16049	ID	Idaho	rural	666	28.5%	8,477.35	0.079	90.5%
16051	ID	Jefferson	urban	405	28.4%	1,093.50	0.371	65.9%
16053	ID	Jerome	rural	361	28.5%	597.18	0.604	57.6%
16055	ID	Kootenai	urban	3,665	28.7%	1,244.12	2.946	58.5%
16057	ID	Latah	rural	796	28.6%	1,076.00	0.740	73.2%
16059	ID	Lemhi	rural	414	28.4%	4,563.39	0.091	91.5%
16061	ID	Lewis	rural	275	28.5%	478.80	0.574	86.7%
16065	ID	Madison	rural	308	28.8%	469.21	0.655	65.4%
16067	ID	Minidoka	rural	440	28.4%	757.59	0.580	65.5%
16069	ID	Nez Perce	urban	1,100	28.7%	848.09	1.296	67.1%
16071	ID	Oneida	rural	120	28.7%	1,200.06	0.100	72.7%
16073	ID	Owyhee	urban	160	28.3%	7,665.51	0.021	46.2%
16075	ID	Payette	rural	443	28.6%	406.87	1.089	51.3%
16077	ID	Power	rural	140	28.6%	1,404.24	0.100	69.7%
16079	ID	Shoshone	rural	379	28.5%	2,629.66	0.144	70.0%
16081	ID	Teton	rural	194	28.3%	449.46	0.432	86.7%
16083	ID	Twin Falls	rural	1,673	28.6%	1921.21	0.871	60.8%
16085	ID	Valley	rural	318	28.7%	3,664.52	0.087	63.9%
16087	ID	Washington	rural	273	28.8%	1,452.98	0.188	55.0%
17001	IL	Adams	rural	1,938	28.6%	855.20	2.266	78.7%
17003	IL	Alexander	urban	191	28.2%	235.51	0.812	83.6%
17005	IL	Bond	urban	428	28.6%	380.28	1.124	78.9%
17007	IL	Boone	urban	920	28.6%	280.72	3.277	62.8%
17009	IL	Brown	rural	134	28.8%	305.61	0.439	78.7%
17011	IL	Bureau	rural	1,103	28.6%	869.03	1.269	80.3%
17013	IL	Calhoun	urban	154	28.6%	253.82	0.606	78.8%
17015	IL	Carroll	rural	510	28.6%	444.81	1.146	70.7%
17017	IL	Cass	rural	317	28.5%	375.82	0.843	75.2%
17019	IL	Champaign	urban	2,077	28.4%	996.27	2.084	42.3%
17021	IL	Christian	rural	938	28.6%	709.38	1.322	76.9%
17023	IL	Clark	rural	467	28.8%	501.42	0.931	73.2%
17025	IL	Clay	rural	427	28.6%	468.32	0.912	85.9%
17027	IL	Clinton	urban	872	28.6%	474.09	1.840	81.8%
17029	IL	Coles	rural	993	28.5%	508.29	1.953	66.0%
17031	IL	Cook	urban	81,910	28.4%	945.33	86.647	65.0%
17033	IL	Crawford	rural	593	28.5%	443.63	1.337	86.4%
17035	IL	Cumberland	rural	260	28.6%	346.02	0.751	72.1%
17039	IL	De Witt	urban	406	28.6%	397.51	1.021	67.4%
17037	IL	DeKalb	urban	1,825	28.5%	631.31	2.891	69.7%
17041	IL	Douglas	rural	292	28.2%	416.66	0.701	51.0%
17043	IL	DuPage	urban	19,811	28.6%	327.50	60.493	69.8%
17045	IL	Edgar	rural	507	28.5%	623.37	0.813	75.6%
17047	IL	Edwards	rural	195	28.5%	222.42	0.878	85.9%
17049	IL	Effingham	rural	1,067	28.6%	478.78	2.230	83.5%
17051	IL	Fayette	rural	508	28.8%	716.48	0.709	81.4%
17053	IL	Ford	urban	327	28.6%	485.62	0.673	69.0%
17055	IL	Franklin	rural	1,092	28.7%	408.89	2.670	71.5%
17057	IL	Fulton	rural	950	28.4%	865.59	1.097	66.3%
17059	IL	Gallatin	rural	189	28.5%	323.07	0.586	81.8%
17061	IL	Greene	rural	389	28.6%	543.02	0.716	81.8%
17063	IL	Grundy	urban	1,118	28.6%	418.04	2.674	82.1%
17065	IL	Hamilton	rural	252	28.5%	434.67	0.581	80.9%
17067	IL	Hancock	rural	675	28.7%	793.73	0.851	81.8%
17069	IL	Hardin	rural	123	28.3%	177.53	0.692	78.3%
17071	IL	Henderson	rural	237	28.5%	378.87	0.626	79.6%
17073	IL	Henry	urban	1,481	28.5%	822.99	1.799	76.3%
17075	IL	Iroquois	rural	907	28.8%	1,117.32	0.812	77.1%
17077	IL	Jackson	urban	1,075	28.5%	584.08	1.841	66.5%
17079	IL	Jasper	rural	264	28.5%	494.51	0.534	83.1%
17081	IL	Jefferson	rural	1,003	28.5%	571.17	1.756	79.1%
17083	IL	Jersey	urban	455	28.5%	369.27	1.231	76.0%
17085	IL	Jo Daviess	rural	632	28.5%	601.09	1.051	54.4%
17087	IL	Johnson	rural	354	28.9%	343.92	1.029	69.5%
17089	IL	Kane	urban	9,257	28.7%	520.06	17.799	67.2%
17091	IL	Kankakee	urban	2,449	28.5%	676.56	3.620	77.0%
17093	IL	Kendall	urban	1,283	28.7%	320.34	4.005	69.4%
17095	IL	Knox	rural	1,414	28.5%	716.39	1.973	65.5%
17099	IL	LaSalle	rural	3,103	28.6%	1,135.12	2.734	80.0%
17097	IL	Lake	urban	13,989	28.7%	443.67	31.530	76.8%
17101	IL	Lawrence	rural	432	28.8%	372.18	1.162	86.3%
17103	IL	Lee	rural	887	28.5%	724.90	1.224	72.4%
17105	IL	Livingston	rural	829	28.6%	1,044.29	0.794	70.0%
17107	IL	Logan	rural	654	28.5%	618.06	1.058	68.2%
17115	IL	Macon	urban	3,016	28.4%	580.69	5.193	79.5%
17117	IL	Macoupin	urban	1,401	28.6%	862.91	1.624	80.2%
17119	IL	Madison	urban	4,961	28.5%	715.58	6.933	57.7%
17121	IL	Marion	rural	1,248	28.4%	572.36	2.181	82.1%
17123	IL	Marshall	urban	365	28.4%	386.79	0.943	75.0%
17125	IL	Mason	rural	506	28.5%	539.24	0.939	78.3%

17127	IL	Massac	rural	390	28.7%	237.22	1.643	77.8%
17109	IL	McDonough	rural	635	28.3%	589.41	1.077	63.8%
17111	IL	McHenry	urban	7,514	28.8%	603.17	12.458	78.5%
17113	IL	McLean	urban	2,828	28.5%	1,183.38	2.390	63.1%
17129	IL	Menard	urban	284	28.5%	314.44	0.902	61.8%
17131	IL	Mercer	urban	450	28.6%	561.20	0.801	70.4%
17133	IL	Monroe	urban	601	28.3%	385.01	1.560	53.6%
17135	IL	Montgomery	rural	898	28.5%	703.69	1.277	81.3%
17137	IL	Morgan	rural	886	28.5%	568.79	1.558	72.2%
17139	IL	Moultrie	rural	447	28.3%	335.94	1.330	77.7%
17141	IL	Ogle	rural	1,130	28.6%	758.57	1.490	65.0%
17143	IL	Peoria	urban	3,858	28.4%	619.21	6.230	62.9%
17145	IL	Perry	rural	515	28.6%	441.76	1.167	73.9%
17147	IL	Piatt	urban	364	28.3%	439.20	0.828	52.1%
17149	IL	Pike	rural	484	28.7%	831.38	0.582	82.5%
17151	IL	Pope	rural	124	28.5%	368.77	0.335	75.9%
17153	IL	Pulaski	rural	159	28.5%	199.18	0.797	77.7%
17155	IL	Putnam	rural	199	28.5%	160.16	1.241	79.4%
17157	IL	Randolph	rural	833	28.6%	575.50	1.447	74.0%
17159	IL	Richland	rural	516	28.6%	359.99	1.434	83.1%
17161	IL	Rock Island	urban	3,714	28.5%	427.64	8.686	69.4%
17165	IL	Saline	rural	732	28.5%	379.82	1.928	77.7%
17167	IL	Sangamon	urban	3,650	28.5%	868.30	4.204	55.4%
17169	IL	Schuyler	rural	213	28.3%	437.27	0.487	78.0%
17171	IL	Scott	rural	138	28.6%	250.91	0.548	76.8%
17173	IL	Shelby	rural	709	28.6%	758.52	0.935	84.7%
17163	IL	St. Clair	urban	4,049	28.4%	657.76	6.155	55.9%
17175	IL	Stark	urban	180	28.2%	288.08	0.626	71.2%
17177	IL	Stephenson	rural	1,167	28.5%	564.52	2.067	54.4%
17179	IL	Tazewell	urban	3,343	28.4%	648.97	5.151	67.5%
17181	IL	Union	rural	439	28.7%	413.46	1.061	68.5%
17183	IL	Vermilion	urban	1,471	28.4%	898.37	1.637	50.9%
17185	IL	Wabash	rural	347	28.2%	223.25	1.555	84.3%
17187	IL	Warren	rural	481	28.6%	542.41	0.887	73.0%
17189	IL	Washington	rural	381	28.3%	562.57	0.678	79.3%
17191	IL	Wayne	rural	530	28.8%	713.81	0.742	85.7%
17193	IL	White	rural	505	28.7%	494.77	1.020	80.8%
17195	IL	Whiteside	rural	1,695	28.5%	684.25	2.477	75.7%
17197	IL	Will	urban	9,993	28.7%	836.91	11.940	72.3%
17199	IL	Williamson	urban	1,573	28.6%	420.15	3.743	72.5%
17201	IL	Winnebago	urban	5,540	28.5%	513.36	10.792	58.9%
17203	IL	Woodford	urban	842	28.4%	527.80	1.595	72.7%
18001	IN	Adams	rural	555	28.6%	339.03	1.638	56.1%
18003	IN	Allen	urban	4,698	28.5%	657.31	7.147	44.1%
18005	IN	Bartholomew	urban	1,890	28.6%	406.91	4.645	72.3%
18007	IN	Benton	urban	231	28.6%	406.42	0.569	76.0%
18009	IN	Blackford	rural	358	28.8%	165.08	2.171	71.7%
18011	IN	Boone	urban	1,058	28.5%	422.91	2.501	64.7%
18013	IN	Brown	urban	266	28.5%	311.98	0.853	66.8%
18015	IN	Carroll	urban	390	28.6%	372.22	1.048	71.4%
18017	IN	Cass	rural	815	28.5%	412.15	1.979	69.0%
18019	IN	Clark	urban	2,275	28.6%	372.86	6.102	65.9%
18021	IN	Clay	urban	741	28.6%	357.54	2.073	75.8%
18023	IN	Clinton	rural	721	28.5%	405.07	1.779	72.2%
18025	IN	Crawford	rural	296	28.4%	305.64	0.970	73.8%
18027	IN	Daviess	rural	673	28.6%	429.49	1.567	83.1%
18033	IN	DeKalb	rural	640	28.6%	362.82	1.763	43.2%
18029	IN	Dearborn	urban	1,082	28.5%	305.03	3.546	65.7%
18031	IN	Decatur	rural	587	28.4%	372.57	1.574	66.9%
18035	IN	Delaware	urban	2,690	28.5%	392.12	6.861	73.6%
18037	IN	Dubois	rural	1,214	28.6%	427.27	2.842	83.6%
18039	IN	Elkhart	urban	3,447	28.5%	463.17	7.443	59.6%
18041	IN	Fayette	rural	644	28.5%	215.01	2.995	76.6%
18043	IN	Floyd	urban	1,676	28.6%	147.93	11.332	69.6%
18045	IN	Fountain	rural	558	28.5%	395.66	1.411	76.6%
18047	IN	Franklin	rural	380	28.5%	384.43	0.988	60.4%
18049	IN	Fulton	rural	364	28.3%	368.39	0.988	50.5%
18051	IN	Gibson	rural	743	28.6%	487.49	1.525	66.8%
18053	IN	Grant	rural	1,662	28.4%	414.07	4.015	69.9%
18055	IN	Greene	rural	859	28.5%	542.49	1.583	79.6%
18057	IN	Hamilton	urban	4,277	28.7%	394.27	10.848	62.1%
18059	IN	Hancock	urban	1,425	28.7%	306.02	4.656	60.1%
18061	IN	Harrison	urban	906	28.5%	484.52	1.870	71.4%
18063	IN	Hendricks	urban	2,396	28.7%	406.91	5.889	60.7%
18065	IN	Henry	rural	1,162	28.6%	391.88	2.964	68.9%
18067	IN	Howard	urban	2,281	28.5%	293.06	7.784	79.6%
18069	IN	Huntington	rural	661	28.4%	382.65	1.727	44.4%
18071	IN	Jackson	rural	892	28.6%	509.31	1.751	61.6%
18073	IN	Jasper	urban	872	28.7%	559.62	1.559	79.7%
18075	IN	Jay	rural	474	28.6%	383.91	1.234	63.8%
18077	IN	Jefferson	rural	897	28.7%	360.63	2.487	79.7%
18079	IN	Jennings	rural	529	28.4%	376.58	1.404	68.6%

18081	IN	Johnson	urban	2,753	28.7%	320.43	8.591	63.2%
18083	IN	Knox	rural	1,047	28.8%	516.03	2.029	84.6%
18085	IN	Kosciusko	rural	1,182	28.4%	531.38	2.224	48.0%
18087	IN	LaGrange	rural	541	28.5%	379.62	1.426	53.4%
18091	IN	LaPorte	urban	2,789	28.6%	598.30	4.662	79.7%
18089	IN	Lake	urban	9,669	28.4%	498.96	19.378	74.5%
18093	IN	Lawrence	rural	1,176	28.6%	449.17	2.618	74.8%
18095	IN	Madison	urban	3,040	28.5%	451.92	6.726	67.0%
18097	IN	Marion	urban	12,752	28.4%	396.30	32.177	58.0%
18099	IN	Marshall	rural	809	28.5%	443.63	1.823	51.6%
18101	IN	Martin	rural	289	29.0%	335.74	0.861	81.3%
18103	IN	Miami	rural	775	28.7%	373.84	2.073	74.9%
18105	IN	Monroe	urban	2,429	28.6%	394.51	6.157	69.8%
18107	IN	Montgomery	rural	861	28.8%	504.61	1.706	62.9%
18109	IN	Morgan	urban	1,521	28.7%	403.97	3.766	66.6%
18111	IN	Newton	urban	313	28.5%	401.76	0.778	80.4%
18113	IN	Noble	rural	643	28.6%	410.84	1.566	47.2%
18115	IN	Ohio	urban	141	28.6%	86.14	1.634	71.7%
18117	IN	Orange	rural	503	28.7%	398.39	1.264	72.9%
18119	IN	Owen	urban	460	28.6%	385.29	1.193	68.5%
18121	IN	Parke	rural	386	28.6%	444.66	0.868	75.8%
18123	IN	Perry	rural	542	28.5%	381.73	1.420	84.5%
18125	IN	Pike	rural	313	28.3%	334.24	0.937	72.7%
18127	IN	Porter	urban	3,540	28.7%	418.15	8.465	74.0%
18129	IN	Posey	urban	537	28.6%	409.57	1.311	69.0%
18131	IN	Pulaski	rural	355	28.3%	433.65	0.818	77.5%
18133	IN	Putnam	urban	674	28.7%	480.53	1.403	63.9%
18135	IN	Randolph	rural	730	28.7%	452.38	1.613	76.8%
18137	IN	Ripley	rural	815	28.7%	446.43	1.825	69.9%
18139	IN	Rush	rural	405	28.7%	408.12	0.992	74.0%
18143	IN	Scott	urban	558	28.6%	190.40	2.928	73.4%
18145	IN	Shelby	urban	838	28.6%	411.15	2.038	66.3%
18147	IN	Spencer	rural	547	28.5%	396.74	1.378	75.7%
18141	IN	St. Joseph	urban	4,973	28.4%	457.85	10.863	57.5%
18149	IN	Starke	rural	553	28.6%	309.13	1.788	77.5%
18151	IN	Steuben	rural	672	28.5%	308.94	2.174	49.4%
18153	IN	Sullivan	urban	516	28.6%	447.14	1.155	79.6%
18155	IN	Switzerland	rural	201	28.5%	220.63	0.909	75.5%
18157	IN	Tippecanoe	urban	2,693	28.6%	499.81	5.388	68.8%
18159	IN	Tipton	rural	402	28.8%	260.54	1.541	76.1%
18161	IN	Union	urban	199	28.5%	161.22	1.231	73.4%
18163	IN	Vanderburgh	urban	3,793	28.5%	233.48	16.244	64.3%
18165	IN	Vermillion	urban	447	28.5%	256.88	1.739	80.0%
18167	IN	Vigo	urban	2,390	28.5%	403.31	5.925	77.4%
18169	IN	Wabash	rural	663	28.4%	412.43	1.608	50.5%
18171	IN	Warren	rural	171	29.0%	364.68	0.468	79.8%
18173	IN	Warrick	urban	1,328	28.6%	384.82	3.450	67.0%
18175	IN	Washington	urban	500	28.6%	513.73	0.974	62.0%
18177	IN	Wayne	rural	1,910	28.7%	401.74	4.754	79.9%
18179	IN	Wells	urban	443	28.4%	368.09	1.202	49.4%
18181	IN	White	rural	765	28.7%	505.12	1.514	76.2%
18183	IN	Whitley	urban	506	28.5%	335.57	1.508	37.8%
20001	KS	Allen	rural	403	28.4%	500.30	0.805	85.3%
20003	KS	Anderson	rural	275	28.6%	579.65	0.474	88.6%
20005	KS	Atchison	rural	469	28.5%	431.17	1.088	86.0%
20007	KS	Barber	rural	174	28.7%	1,134.07	0.153	88.6%
20009	KS	Barton	rural	817	28.4%	895.40	0.912	90.7%
20011	KS	Bourbon	rural	411	28.4%	635.47	0.647	82.3%
20013	KS	Brown	rural	317	28.7%	570.87	0.556	91.0%
20015	KS	Butler	urban	1,424	28.4%	1,429.86	0.996	76.1%
20019	KS	Chautauqua	rural	132	28.4%	638.88	0.207	92.3%
20021	KS	Cherokee	rural	549	28.6%	587.57	0.935	82.4%
20023	KS	Cheyenne	rural	136	28.3%	1,019.89	0.134	92.0%
20027	KS	Clay	rural	302	28.6%	645.30	0.468	87.7%
20029	KS	Cloud	rural	362	28.7%	715.34	0.507	89.3%
20031	KS	Coffey	rural	290	28.6%	626.95	0.462	88.0%
20035	KS	Cowley	rural	1,009	28.5%	1,125.75	0.897	88.7%
20037	KS	Crawford	rural	1,019	28.6%	589.76	1.727	85.7%
20039	KS	Decatur	rural	134	28.6%	893.52	0.150	88.2%
20041	KS	Dickinson	rural	633	28.5%	847.07	0.747	90.2%
20043	KS	Doniphan	urban	244	28.5%	393.41	0.620	89.6%
20045	KS	Douglas	urban	2,239	28.6%	455.87	4.911	75.1%
20047	KS	Edwards	rural	109	28.8%	621.89	0.176	89.6%
20049	KS	Elk	rural	124	28.2%	644.26	0.192	91.8%
20051	KS	Ellis	rural	773	28.5%	899.91	0.858	86.7%
20053	KS	Ellsworth	rural	203	28.6%	715.85	0.283	91.7%
20055	KS	Finney	rural	624	28.3%	1,301.97	0.479	81.4%
20057	KS	Ford	rural	645	28.3%	1,098.27	0.587	85.7%
20059	KS	Franklin	rural	689	28.7%	571.76	1.204	82.5%
20061	KS	Geary	rural	451	28.3%	384.62	1.172	81.5%
20063	KS	Gove	rural	114	28.5%	1,071.66	0.107	89.3%
20065	KS	Graham	rural	107	28.4%	898.52	0.119	88.5%

20067	KS	Grant	rural	161	28.4%	574.80	0.280	86.8%
20069	KS	Gray	rural	194	28.9%	868.87	0.223	91.4%
20073	KS	Greenwood	rural	273	28.5%	1,143.30	0.239	91.0%
20077	KS	Harper	rural	209	28.6%	801.27	0.261	93.6%
20079	KS	Harvey	urban	1,002	28.5%	539.75	1.857	73.1%
20085	KS	Jackson	urban	386	28.8%	656.22	0.589	84.8%
20087	KS	Jefferson	urban	536	28.6%	532.57	1.006	80.2%
20089	KS	Jewell	rural	146	28.1%	909.78	0.160	89.5%
20091	KS	Johnson	urban	10,033	28.6%	473.37	21.195	57.9%
20095	KS	Kingman	urban	246	28.3%	863.36	0.285	89.8%
20099	KS	Labette	rural	597	28.2%	645.29	0.925	83.7%
20103	KS	Leavenworth	urban	1,491	28.6%	462.83	3.222	76.1%
20105	KS	Lincoln	rural	118	29.2%	719.40	0.164	89.8%
20107	KS	Linn	urban	262	28.5%	594.06	0.442	67.1%
20111	KS	Lyon	rural	884	28.6%	847.47	1.043	86.7%
20115	KS	Marion	rural	488	28.6%	944.29	0.517	90.9%
20117	KS	Marshall	rural	403	28.6%	900.18	0.448	89.9%
20113	KS	McPherson	rural	974	28.5%	898.27	1.084	83.8%
20119	KS	Meade	rural	141	28.2%	978.09	0.144	89.7%
20121	KS	Miami	urban	592	28.4%	575.66	1.028	67.1%
20123	KS	Mitchell	rural	268	28.5%	701.79	0.382	91.3%
20125	KS	Montgomery	rural	1,119	28.6%	643.53	1.738	88.8%
20127	KS	Morris	rural	267	28.6%	695.28	0.384	91.9%
20131	KS	Nemaha	rural	383	28.5%	717.43	0.534	92.6%
20133	KS	Neosho	rural	510	28.6%	571.47	0.893	89.1%
20135	KS	Ness	rural	125	28.6%	1,074.75	0.116	89.0%
20137	KS	Norton	rural	194	28.9%	878.13	0.221	89.0%
20139	KS	Osage	urban	509	28.6%	705.52	0.722	88.6%
20141	KS	Osborne	rural	165	28.7%	892.50	0.185	90.7%
20143	KS	Ottawa	rural	205	28.8%	720.73	0.285	92.0%
20145	KS	Pawnee	rural	118	27.9%	754.26	0.156	93.6%
20147	KS	Phillips	rural	228	28.8%	885.88	0.257	91.8%
20149	KS	Pottawatomie	urban	475	28.5%	841.02	0.565	83.0%
20151	KS	Pratt	rural	307	28.5%	735.04	0.418	93.4%
20153	KS	Rawlins	rural	120	28.3%	1,069.42	0.112	92.6%
20155	KS	Reno	rural	1,968	28.6%	1,255.35	1.568	85.7%
20157	KS	Republic	rural	249	28.6%	717.37	0.347	93.2%
20159	KS	Rice	rural	334	28.7%	726.24	0.459	90.2%
20161	KS	Riley	urban	1,186	28.7%	609.77	1.945	80.9%
20163	KS	Rooks	rural	201	28.6%	890.53	0.226	92.1%
20165	KS	Rush	rural	137	28.5%	717.76	0.190	90.9%
20167	KS	Russell	rural	272	28.5%	886.26	0.307	91.5%
20169	KS	Saline	rural	1,578	28.5%	720.22	2.191	81.9%
20171	KS	Scott	rural	106	28.7%	717.54	0.148	87.9%
20173	KS	Sedgwick	urban	9,679	28.5%	997.51	9.703	70.0%
20175	KS	Seward	rural	320	28.4%	639.50	0.500	83.9%
20177	KS	Shawnee	urban	4,936	28.6%	544.02	9.073	82.1%
20181	KS	Sherman	rural	195	28.6%	1,056.07	0.185	91.7%
20183	KS	Smith	rural	198	28.3%	895.46	0.221	91.1%
20185	KS	Stafford	rural	238	28.9%	792.05	0.300	89.7%
20189	KS	Stevens	rural	120	28.7%	727.29	0.165	85.6%
20191	KS	Sumner	urban	723	28.6%	1,181.94	0.612	79.7%
20193	KS	Thomas	rural	219	28.9%	1,074.69	0.204	90.1%
20195	KS	Trego	rural	106	28.5%	889.48	0.119	92.8%
20197	KS	Wabaunsee	urban	226	28.5%	794.30	0.285	88.3%
20201	KS	Washington	rural	230	28.1%	894.76	0.257	89.4%
20205	KS	Wilson	rural	287	28.6%	570.42	0.504	92.0%
20207	KS	Woodson	rural	123	28.8%	497.82	0.247	88.4%
20209	KS	Wyandotte	urban	1,926	28.2%	151.60	12.706	52.3%
21001	KY	Adair	rural	390	28.3%	405.28	0.963	69.9%
21003	KY	Allen	urban	411	28.7%	344.34	1.194	69.9%
21005	KY	Anderson	rural	320	28.2%	201.83	1.584	49.7%
21007	KY	Ballard	rural	269	28.6%	246.66	1.091	72.9%
21009	KY	Barren	rural	899	28.5%	487.54	1.844	67.7%
21011	KY	Bath	rural	206	28.1%	278.79	0.740	56.9%
21013	KY	Bell	rural	535	28.3%	359.00	1.491	69.2%
21015	KY	Boone	urban	1,684	28.6%	246.36	6.835	48.3%
21017	KY	Bourbon	urban	357	28.2%	289.72	1.231	54.8%
21019	KY	Boyd	urban	974	28.5%	159.86	6.095	66.2%
21021	KY	Boyle	rural	684	28.6%	180.17	3.799	62.8%
21023	KY	Bracken	urban	182	28.2%	205.61	0.884	60.3%
21025	KY	Breathitt	rural	203	28.2%	492.41	0.413	60.7%
21027	KY	Breckinridge	rural	465	28.6%	567.17	0.820	74.8%
21029	KY	Bullitt	urban	1,032	28.6%	297.02	3.475	58.3%
21031	KY	Butler	urban	182	28.1%	426.09	0.428	58.7%
21033	KY	Caldwell	rural	321	28.4%	344.79	0.931	69.3%
21035	KY	Calloway	rural	803	28.5%	385.02	2.086	64.6%
21037	KY	Campbell	urban	1,314	28.5%	151.31	8.682	48.0%
21039	KY	Carlisle	rural	132	27.9%	189.43	0.694	70.7%
21041	KY	Carroll	rural	212	28.5%	128.57	1.650	64.9%
21043	KY	Carter	rural	433	29.0%	409.50	1.059	63.6%
21045	KY	Casey	rural	364	28.7%	444.23	0.819	72.5%

21047	KY	Christian	urban	1,020	28.4%	717.50	1.421	67.5%
21049	KY	Clark	urban	667	28.4%	252.46	2.643	57.7%
21051	KY	Clay	rural	328	28.3%	469.25	0.699	68.8%
21053	KY	Clinton	rural	258	28.6%	197.25	1.309	71.7%
21055	KY	Crittenden	rural	226	29.1%	359.95	0.628	74.9%
21057	KY	Cumberland	rural	181	28.5%	305.18	0.595	68.0%
21059	KY	Daviess	urban	2,102	28.5%	458.35	4.587	68.2%
21061	KY	Edmonson	urban	190	28.4%	302.88	0.627	67.6%
21063	KY	Elliott	rural	246	28.5%	234.32	1.048	60.7%
21065	KY	Estill	rural	299	28.7%	253.08	1.182	65.3%
21067	KY	Fayette	urban	4,312	28.5%	283.65	15.203	55.0%
21069	KY	Fleming	rural	300	28.4%	348.54	0.862	60.8%
21071	KY	Floyd	rural	630	28.7%	393.34	1.601	61.6%
21073	KY	Franklin	rural	896	28.3%	207.75	4.315	45.5%
21075	KY	Fulton	rural	208	28.3%	205.50	1.011	77.4%
21077	KY	Gallatin	urban	107	28.1%	101.23	1.055	55.8%
21079	KY	Garrard	rural	327	28.4%	230.08	1.422	62.0%
21081	KY	Grant	urban	384	28.5%	257.96	1.488	51.7%
21083	KY	Graves	rural	875	28.4%	551.74	1.586	65.1%
21085	KY	Grayson	rural	660	28.7%	496.70	1.329	72.9%
21087	KY	Green	rural	270	28.5%	286.03	0.942	71.4%
21089	KY	Greenup	urban	952	28.6%	344.40	2.765	65.8%
21091	KY	Hancock	urban	214	28.6%	187.65	1.143	76.0%
21093	KY	Hardin	urban	2,026	28.5%	623.28	3.251	74.5%
21095	KY	Harlan	rural	551	28.4%	465.83	1.182	68.6%
21097	KY	Harrison	rural	380	28.4%	306.36	1.240	63.2%
21099	KY	Hart	rural	362	28.5%	412.09	0.878	71.8%
21101	KY	Henderson	urban	919	28.4%	436.67	2.105	62.4%
21103	KY	Henry	urban	315	28.4%	286.28	1.099	53.5%
21107	KY	Hopkins	rural	1,081	28.4%	542.00	1.995	68.5%
21109	KY	Jackson	rural	243	28.4%	345.20	0.703	67.8%
21111	KY	Jefferson	urban	13,846	28.5%	380.42	36.396	58.7%
21113	KY	Jessamine	urban	747	28.3%	172.12	4.342	56.0%
21115	KY	Johnson	rural	410	28.7%	261.95	1.565	63.0%
21117	KY	Kenton	urban	2,272	28.5%	160.25	14.176	51.8%
21119	KY	Knott	rural	207	28.3%	351.52	0.590	62.1%
21121	KY	Knox	rural	425	28.6%	386.30	1.099	68.9%
21123	KY	Larue	urban	325	28.6%	261.52	1.244	70.0%
21125	KY	Laurel	rural	929	28.5%	433.95	2.141	65.5%
21127	KY	Lawrence	rural	316	28.4%	415.60	0.761	72.5%
21129	KY	Lee	rural	146	28.5%	208.86	0.699	64.8%
21131	KY	Leslie	rural	189	28.5%	400.84	0.472	66.9%
21133	KY	Letcher	rural	397	28.5%	337.91	1.174	64.6%
21135	KY	Lewis	rural	261	28.4%	482.84	0.540	66.3%
21137	KY	Lincoln	rural	555	28.5%	334.09	1.663	62.7%
21139	KY	Livingston	rural	287	28.8%	313.13	0.916	73.6%
21141	KY	Logan	rural	650	28.7%	552.13	1.178	71.3%
21143	KY	Lyon	rural	229	28.9%	213.84	1.070	63.8%
21151	KY	Madison	rural	1,232	28.4%	437.29	2.817	54.6%
21153	KY	Magoffin	rural	204	28.6%	308.44	0.661	65.8%
21155	KY	Marion	rural	414	28.3%	343.01	1.208	69.4%
21157	KY	Marshall	rural	922	28.8%	301.25	3.061	69.7%
21159	KY	Martin	rural	173	28.8%	229.60	0.756	65.4%
21161	KY	Mason	rural	362	28.2%	240.13	1.507	62.2%
21145	KY	McCracken	rural	1,683	28.5%	248.74	6.767	70.3%
21147	KY	McCreary	rural	273	28.5%	426.80	0.640	66.0%
21149	KY	McLean	urban	227	28.2%	252.47	0.899	65.2%
21163	KY	Meade	urban	402	28.5%	305.42	1.315	70.2%
21165	KY	Menifee	rural	144	28.3%	203.58	0.707	54.9%
21167	KY	Mercer	rural	481	28.5%	248.80	1.935	60.0%
21169	KY	Metcalfe	rural	212	28.3%	289.65	0.733	56.9%
21171	KY	Monroe	rural	307	28.6%	329.37	0.931	73.1%
21173	KY	Montgomery	rural	460	28.3%	197.37	2.331	57.2%
21175	KY	Morgan	rural	208	28.6%	381.13	0.545	57.0%
21177	KY	Muhlenberg	rural	715	28.8%	467.08	1.531	73.1%
21179	KY	Nelson	rural	970	28.6%	417.51	2.323	68.7%
21181	KY	Nicholas	rural	154	28.5%	195.17	0.789	65.7%
21183	KY	Ohio	rural	412	28.5%	587.27	0.701	64.2%
21185	KY	Oldham	urban	789	28.6%	187.22	4.215	54.8%
21187	KY	Owen	rural	156	28.2%	351.10	0.444	59.4%
21189	KY	Owsley	rural	102	28.0%	197.41	0.515	67.6%
21191	KY	Pendleton	urban	222	28.3%	277.16	0.802	53.9%
21193	KY	Perry	rural	569	28.8%	339.67	1.674	67.6%
21195	KY	Pike	rural	1,068	28.4%	786.83	1.357	65.9%
21197	KY	Powell	rural	200	28.7%	178.98	1.120	57.0%
21199	KY	Pulaski	rural	1,502	28.7%	658.41	2.282	61.7%
21203	KY	Rockcastle	rural	323	28.8%	316.54	1.021	67.7%
21205	KY	Rowan	rural	307	28.5%	279.80	1.095	48.0%
21207	KY	Russell	rural	433	28.4%	253.66	1.706	68.5%
21209	KY	Scott	urban	636	28.5%	281.77	2.256	51.8%
21211	KY	Shelby	urban	705	28.4%	379.64	1.858	58.4%
21213	KY	Simpson	rural	339	28.6%	234.20	1.449	70.3%

21215	KY	Spencer	urban	266	28.3%	186.68	1.424	59.2%
21217	KY	Taylor	rural	669	28.6%	266.33	2.511	69.0%
21219	KY	Todd	rural	229	28.6%	374.50	0.612	72.1%
21221	KY	Trigg	urban	371	28.5%	441.43	0.840	63.8%
21223	KY	Trimble	urban	171	28.6%	151.65	1.126	65.6%
21225	KY	Union	rural	326	28.2%	342.85	0.951	70.0%
21227	KY	Warren	urban	2,088	28.3%	541.60	3.855	64.8%
21229	KY	Washington	rural	255	28.4%	297.27	0.856	68.4%
21231	KY	Wayne	rural	413	28.7%	458.17	0.902	63.6%
21233	KY	Webster	rural	296	28.2%	331.94	0.892	63.8%
21235	KY	Whitley	rural	976	28.7%	437.83	2.229	68.3%
21237	KY	Wolfe	rural	144	28.5%	222.17	0.650	60.1%
21239	KY	Woodford	urban	466	28.5%	188.78	2.467	50.6%
22001	LA	Acadia	urban	1,157	28.4%	655.12	1.766	82.2%
22003	LA	Allen	rural	435	28.4%	761.85	0.571	78.2%
22005	LA	Ascension	urban	830	28.4%	289.98	2.863	31.7%
22007	LA	Assumption	rural	343	28.2%	338.66	1.013	62.7%
22009	LA	Avoyelles	rural	908	28.3%	832.43	1.091	77.9%
22011	LA	Beauregard	rural	826	28.8%	1,157.34	0.714	80.0%
22013	LA	Bienville	rural	346	28.1%	811.27	0.426	72.5%
22015	LA	Bossier	urban	2,099	28.5%	840.06	2.499	71.2%
22017	LA	Caddo	urban	5,013	28.3%	878.54	5.706	67.1%
22019	LA	Calcasieu	urban	4,125	28.4%	1,063.66	3.878	75.5%
22021	LA	Caldwell	rural	231	28.3%	529.42	0.437	70.7%
22025	LA	Catahoula	rural	245	28.5%	708.03	0.346	76.8%
22027	LA	Claiborne	rural	351	28.1%	754.88	0.465	76.8%
22029	LA	Concordia	rural	417	28.3%	696.92	0.599	74.8%
22031	LA	De Soto	urban	620	28.2%	875.58	0.708	75.9%
22033	LA	East Baton Rouge	urban	5,245	28.4%	455.37	11.518	43.8%
22035	LA	East Carroll	rural	127	28.2%	420.7	0.303	77.8%
22037	LA	East Feliciana	urban	309	28.2%	453.41	0.681	48.5%
22039	LA	Evangeline	rural	682	28.4%	662.38	1.029	83.4%
22041	LA	Franklin	rural	461	28.2%	624.59	0.738	75.0%
22043	LA	Grant	urban	417	29.0%	643.03	0.649	73.3%
22045	LA	Iberia	urban	1,460	28.4%	574.11	2.544	81.0%
22047	LA	Iberville	urban	350	28.2%	618.63	0.566	37.1%
22049	LA	Jackson	rural	389	28.5%	569.18	0.683	70.8%
22051	LA	Jefferson	urban	4,219	28.5%	295.63	14.271	27.9%
22053	LA	Jefferson Davis	rural	665	28.5%	651.33	1.021	84.6%
22059	LA	LaSalle	rural	326	28.6%	624.68	0.522	77.6%
22055	LA	Lafayette	urban	4,260	28.4%	268.72	15.852	77.4%
22057	LA	Lafourche	urban	1,590	28.4%	1,068.21	1.489	65.7%
22061	LA	Lincoln	rural	832	28.2%	471.74	1.764	72.0%
22063	LA	Livingston	urban	1,196	28.5%	648.17	1.845	35.8%
22065	LA	Madison	rural	209	27.8%	624.44	0.335	79.1%
22067	LA	Morehouse	rural	546	28.4%	794.93	0.687	65.5%
22069	LA	Natchitoches	rural	785	28.3%	1,252.25	0.627	74.9%
22071	LA	Orleans	urban	3,300	28.3%	169.42	19.478	36.2%
22073	LA	Ouachita	urban	2,783	28.5%	610.41	4.559	67.2%
22075	LA	Plaquemines	urban	171	27.9%	779.91	0.219	29.5%
22077	LA	Pointe Coupee	urban	324	28.2%	557.35	0.582	44.4%
22079	LA	Rapides	urban	2,822	28.5%	1,317.96	2.141	74.5%
22081	LA	Red River	rural	178	28.9%	389.09	0.456	70.6%
22083	LA	Richland	rural	436	28.6%	559.04	0.781	70.5%
22085	LA	Sabine	rural	624	28.6%	866.66	0.720	78.6%
22087	LA	St. Bernard	urban	307	28.5%	377.52	0.812	37.0%
22089	LA	St. Charles	urban	393	28.3%	279.08	1.409	29.4%
22093	LA	St. James	urban	276	28.5%	241.54	1.141	43.7%
22095	LA	St. John the Baptist	urban	382	28.4%	213.07	1.791	34.1%
22097	LA	St. Landry	rural	1,971	28.3%	923.88	2.133	80.4%
22099	LA	St. Martin	urban	922	28.4%	737.65	1.250	78.2%
22101	LA	St. Mary	rural	1,012	28.3%	555.38	1.823	72.9%
22103	LA	St. Tammany	urban	3,158	28.5%	845.55	3.735	37.1%
22105	LA	Tangipahoa	urban	1,724	28.3%	791.27	2.179	52.2%
22107	LA	Tensas	rural	129	28.2%	602.78	0.214	75.9%
22109	LA	Terrebonne	urban	2,107	28.5%	1,231.81	1.711	73.2%
22111	LA	Union	urban	549	28.4%	876.99	0.626	71.0%
22113	LA	Vermilion	urban	1,336	28.5%	1,173.20	1.139	85.7%
22115	LA	Vernon	rural	737	28.6%	1,327.91	0.555	82.7%
22117	LA	Washington	rural	784	28.3%	669.52	1.171	60.5%
22119	LA	Webster	urban	1,046	28.3%	593.03	1.764	71.9%
22121	LA	West Baton Rouge	urban	226	28.2%	192.39	1.175	35.2%
22123	LA	West Carroll	rural	285	28.9%	359.65	0.793	81.0%
22125	LA	West Feliciana	urban	173	28.2%	403.21	0.428	53.8%
22127	LA	Winn	rural	303	28.5%	950.09	0.319	76.8%
25001	MA	Barnstable	urban	11,052	28.7%	393.72	28.070	78.4%
25003	MA	Berkshire	urban	4,609	28.6%	926.82	4.973	84.8%
25005	MA	Bristol	urban	11,768	28.7%	553.10	21.276	68.7%
25007	MA	Dukes	rural	763	28.6%	103.25	7.391	88.7%
25009	MA	Essex	urban	16,966	28.6%	492.56	34.445	66.8%
25011	MA	Franklin	rural	1,855	28.6%	699.32	2.653	63.8%
25013	MA	Hampden	urban	8,653	28.5%	617.14	14.021	57.8%

25015	MA	Hampshire	urban	3,607	28.6%	527.26	6.841	66.3%
25017	MA	Middlesex	urban	28,457	28.6%	817.82	34.796	58.5%
25019	MA	Nantucket	rural	322	28.6%	44.97	7.165	88.5%
25021	MA	Norfolk	urban	14,396	28.6%	396.11	36.343	64.0%
25023	MA	Plymouth	urban	12,434	28.7%	659.08	18.866	70.4%
25025	MA	Suffolk	urban	9,519	28.3%	58.15	163.704	63.0%
25027	MA	Worcester	urban	12,280	28.6%	1,510.77	8.128	47.7%
24001	MD	Allegany	urban	2,271	28.6%	424.16	5.355	88.2%
24003	MD	Anne Arundel	urban	11,727	28.6%	414.9	28.266	78.0%
24005	MD	Baltimore	urban	17,432	28.4%	598.30	29.135	76.5%
24510	MD	Baltimore	urban	10,045	28.2%	80.94	124.103	71.3%
24009	MD	Calvert	urban	1,791	28.4%	213.15	8.405	80.5%
24011	MD	Caroline	rural	809	28.5%	319.42	2.531	87.0%
24013	MD	Carroll	urban	4,491	28.7%	447.59	10.033	80.2%
24015	MD	Cecil	urban	2,199	28.6%	346.27	6.350	84.3%
24017	MD	Charles	urban	2,063	28.5%	457.75	4.508	75.2%
24019	MD	Dorchester	rural	963	28.5%	540.77	1.780	87.3%
24021	MD	Frederick	urban	4,434	28.7%	660.22	6.715	76.7%
24023	MD	Garrett	rural	855	28.7%	647.10	1.321	86.2%
24025	MD	Harford	urban	5,669	28.7%	437.09	12.970	80.2%
24027	MD	Howard	urban	4,585	28.6%	250.74	18.285	71.2%
24029	MD	Kent	rural	935	28.7%	277.03	3.375	88.1%
24031	MD	Montgomery	urban	18,453	28.6%	491.25	37.562	66.5%
24033	MD	Prince George's	urban	10,991	28.3%	482.69	22.770	60.2%
24035	MD	Queen Anne's	urban	1,258	28.8%	371.91	3.381	85.4%
24039	MD	Somerset	urban	596	28.4%	319.72	1.864	87.4%
24037	MD	St. Mary's	urban	1,821	28.6%	357.18	5.098	83.2%
24041	MD	Talbot	rural	1,710	28.7%	268.54	6.367	88.1%
24043	MD	Washington	urban	3,240	28.6%	457.78	7.079	77.8%
24045	MD	Wicomico	urban	2,337	28.4%	374.44	6.241	86.5%
24047	MD	Worcester	urban	2,199	28.7%	468.28	4.695	89.6%
23001	ME	Androscoggin	urban	2,201	28.6%	467.93	4.704	57.0%
23003	ME	Aroostook	rural	2,216	28.5%	6,671.33	0.332	75.6%
23005	ME	Cumberland	urban	6,290	28.6%	835.24	7.531	57.4%
23007	ME	Franklin	rural	740	28.7%	1,696.61	0.436	60.6%
23009	ME	Hancock	rural	1,843	28.8%	1,586.89	1.161	70.7%
23011	ME	Kennebec	rural	2,655	28.6%	867.52	3.060	55.2%
23013	ME	Knox	rural	1,373	28.5%	365.13	3.760	67.2%
23015	ME	Lincoln	rural	1,229	28.7%	455.82	2.697	62.4%
23017	ME	Oxford	rural	1,489	28.6%	2,076.84	0.717	66.4%
23019	ME	Penobscot	urban	3,542	28.5%	3,397.36	1.043	64.1%
23021	ME	Piscataquis	rural	608	28.6%	3,960.86	0.154	72.6%
23023	ME	Sagadahoc	urban	926	28.6%	253.69	3.652	59.1%
23025	ME	Somerset	rural	1,374	28.4%	3,924.40	0.350	70.3%
23027	ME	Waldo	rural	1,087	28.5%	729.92	1.489	63.8%
23029	ME	Washington	rural	1,149	28.6%	2,562.66	0.448	77.7%
23031	ME	York	urban	5,238	28.7%	990.71	5.287	65.3%
26001	MI	Alcona	rural	442	28.7%	674.59	0.655	66.5%
26003	MI	Alger	rural	314	28.3%	915.07	0.344	69.3%
26005	MI	Allegan	rural	1,280	28.4%	825.23	1.551	39.0%
26007	MI	Alpena	rural	1,000	28.6%	571.86	1.748	72.0%
26009	MI	Antrim	rural	648	28.7%	475.70	1.363	58.7%
26011	MI	Arenac	rural	466	28.3%	363.19	1.283	62.5%
26013	MI	Baraga	rural	231	28.7%	898.26	0.257	67.8%
26015	MI	Barry	urban	864	28.6%	553.09	1.562	47.3%
26017	MI	Bay	urban	2,233	28.5%	442.30	5.048	59.1%
26019	MI	Benzie	rural	475	28.6%	319.70	1.487	55.9%
26021	MI	Berrien	urban	3,965	28.5%	567.75	6.984	63.4%
26023	MI	Branch	rural	985	28.7%	506.37	1.946	69.0%
26025	MI	Calhoun	urban	2,985	28.5%	706.23	4.227	68.2%
26027	MI	Cass	urban	812	28.4%	490.06	1.657	61.6%
26029	MI	Charlevoix	rural	760	28.5%	416.34	1.825	60.1%
26031	MI	Cheboygan	rural	746	28.7%	715.26	1.043	65.1%
26033	MI	Chippewa	rural	764	28.6%	1,558.42	0.490	63.6%
26035	MI	Clare	rural	812	28.6%	564.32	1.439	64.6%
26037	MI	Clinton	urban	1,101	28.5%	566.41	1.943	56.7%
26039	MI	Crawford	rural	317	28.7%	556.28	0.570	64.3%
26041	MI	Delta	rural	1,178	28.5%	1,171.10	1.005	66.7%
26043	MI	Dickinson	rural	751	28.5%	761.40	0.986	65.5%
26045	MI	Eaton	urban	1,664	28.7%	575.17	2.894	57.8%
26047	MI	Emmet	rural	1,040	28.6%	467.49	2.224	62.4%
26049	MI	Genesee	urban	5,614	28.5%	636.98	8.813	48.5%
26051	MI	Gladwin	rural	790	28.7%	501.78	1.575	62.2%
26053	MI	Gogebic	rural	521	28.5%	1,101.85	0.473	65.1%
26055	MI	Grand Traverse	rural	2,257	28.6%	464.33	4.860	54.4%
26057	MI	Gratiot	rural	889	28.7%	568.46	1.564	65.2%
26059	MI	Hillsdale	rural	970	28.6%	598.13	1.621	63.1%
26061	MI	Houghton	rural	758	28.7%	1,009.10	0.751	63.9%
26063	MI	Huron	rural	1,004	28.7%	835.71	1.201	70.3%
26065	MI	Ingham	urban	5,230	28.5%	556.12	9.404	58.8%
26067	MI	Ionia	rural	938	28.6%	571.30	1.642	54.7%
26069	MI	Iosco	rural	910	28.5%	549.10	1.658	65.6%

26071	MI	Iron	rural	436	28.2%	1,166.15	0.374	69.8%
26073	MI	Isabella	rural	949	28.6%	572.68	1.658	58.0%
26075	MI	Jackson	urban	3,305	28.5%	701.67	4.710	63.1%
26077	MI	Kalamazoo	urban	3,728	28.5%	561.66	6.637	49.0%
26079	MI	Kalkaska	rural	336	28.6%	559.86	0.601	63.8%
26081	MI	Kent	urban	6,248	28.3%	846.95	7.377	34.7%
26083	MI	Keweenaw	rural	132	28.6%	540.11	0.245	65.0%
26085	MI	Lake	rural	307	28.6%	567.37	0.542	64.3%
26087	MI	Lapeer	urban	1,402	28.7%	643.01	2.180	54.0%
26089	MI	Leelanau	rural	586	28.5%	347.17	1.687	56.2%
26091	MI	Lenawee	rural	2,102	28.7%	749.55	2.804	59.5%
26093	MI	Livingston	urban	2,614	28.7%	565.25	4.624	52.6%
26095	MI	Luce	rural	176	28.7%	899.08	0.196	71.7%
26097	MI	Mackinac	rural	329	28.5%	1,021.57	0.322	63.5%
26099	MI	Macomb	urban	12,436	28.7%	479.22	25.951	52.4%
26101	MI	Manistee	rural	707	28.6%	542.15	1.304	61.9%
26103	MI	Marquette	rural	1,581	28.4%	1,808.40	0.874	64.1%
26105	MI	Mason	rural	830	28.7%	495.07	1.677	63.4%
26107	MI	Mecosta	rural	830	28.5%	555.07	1.496	53.2%
26109	MI	Menominee	rural	605	28.6%	1,044.08	0.580	57.2%
26111	MI	Midland	urban	1,565	28.7%	516.25	3.031	57.0%
26113	MI	Missaukee	rural	318	28.5%	564.73	0.563	64.2%
26115	MI	Monroe	urban	2,230	28.5%	549.39	4.060	52.8%
26117	MI	Montcalm	urban	1,203	28.5%	705.40	1.705	53.7%
26119	MI	Montmorency	rural	415	28.8%	546.66	0.758	66.2%
26121	MI	Muskegon	urban	2,544	28.3%	499.25	5.097	45.7%
26123	MI	Newaygo	rural	668	28.4%	813.20	0.822	40.8%
26125	MI	Oakland	urban	18,200	28.6%	867.66	20.975	51.6%
26127	MI	Oceana	rural	604	28.7%	512.07	1.180	53.8%
26129	MI	Ogemaw	rural	641	28.6%	563.49	1.137	69.5%
26131	MI	Ontonagon	rural	306	28.5%	1,311.22	0.234	71.4%
26133	MI	Osceola	rural	623	28.7%	566.39	1.100	59.5%
26135	MI	Oscoda	rural	228	28.6%	565.73	0.403	70.7%
26137	MI	Otsego	rural	623	28.7%	514.97	1.209	62.0%
26139	MI	Ottawa	urban	2,771	28.4%	563.47	4.917	29.8%
26141	MI	Presque Isle	rural	510	28.5%	658.72	0.773	68.0%
26143	MI	Roscommon	rural	945	28.7%	519.64	1.818	64.0%
26145	MI	Saginaw	urban	3,558	28.5%	800.11	4.447	55.2%
26151	MI	Sanilac	rural	945	28.6%	962.57	0.982	64.8%
26153	MI	Schoolcraft	rural	306	28.4%	1,171.36	0.261	73.2%
26155	MI	Shiawassee	rural	1,379	28.6%	530.67	2.599	55.4%
26147	MI	St. Clair	urban	3,083	28.6%	721.17	4.275	57.0%
26149	MI	St. Joseph	rural	1,304	28.7%	500.59	2.605	65.1%
26157	MI	Tuscola	rural	1,206	28.6%	803.13	1.502	61.5%
26159	MI	Van Buren	urban	1,632	28.5%	607.47	2.686	56.5%
26161	MI	Washtenaw	urban	5,308	28.6%	705.97	7.519	56.6%
26163	MI	Wayne	urban	20,256	28.4%	612.08	33.094	49.5%
26165	MI	Wexford	rural	800	28.5%	565.00	1.416	62.4%
27001	MN	Aitkin	rural	362	28.6%	1,821.66	0.199	34.9%
27003	MN	Anoka	urban	2,167	28.4%	423.01	5.122	24.5%
27005	MN	Becker	rural	485	28.3%	1,315.20	0.369	34.3%
27007	MN	Beltrami	rural	615	28.5%	2,504.94	0.246	41.0%
27009	MN	Benton	urban	240	28.2%	408.30	0.588	26.7%
27011	MN	Big Stone	rural	122	28.4%	499.02	0.244	44.6%
27013	MN	Blue Earth	urban	792	28.4%	747.84	1.059	37.2%
27015	MN	Brown	rural	537	28.4%	611.09	0.879	46.1%
27017	MN	Carlton	urban	511	28.5%	861.38	0.593	38.1%
27019	MN	Carver	urban	576	28.4%	354.33	1.625	26.5%
27021	MN	Cass	rural	523	28.6%	2,021.54	0.259	37.2%
27023	MN	Chippewa	rural	169	28.5%	581.12	0.292	35.0%
27025	MN	Chisago	urban	516	28.2%	414.86	1.244	26.3%
27027	MN	Clay	urban	680	28.3%	1,045.37	0.651	40.0%
27029	MN	Clearwater	rural	145	28.5%	998.94	0.145	37.2%
27031	MN	Cook	rural	132	28.4%	1,452.28	0.091	41.2%
27033	MN	Cottonwood	rural	180	28.1%	638.61	0.281	45.9%
27035	MN	Crow Wing	rural	1,105	28.3%	999.09	1.106	34.7%
27037	MN	Dakota	urban	2,507	28.5%	562.17	4.459	28.9%
27039	MN	Dodge	urban	232	28.3%	439.28	0.529	39.3%
27041	MN	Douglas	rural	511	28.3%	637.30	0.801	26.9%
27043	MN	Faribault	rural	250	28.1%	712.48	0.350	36.0%
27045	MN	Fillmore	urban	420	28.1%	861.30	0.488	43.5%
27047	MN	Freeborn	rural	480	28.3%	707.09	0.678	34.7%
27049	MN	Goodhue	rural	632	28.3%	756.84	0.835	33.8%
27051	MN	Grant	rural	125	28.2%	548.16	0.227	41.4%
27053	MN	Hennepin	urban	10,796	28.3%	553.59	19.502	28.9%
27055	MN	Houston	urban	381	28.4%	552.06	0.690	44.5%
27057	MN	Hubbard	rural	379	28.7%	925.67	0.410	38.2%
27059	MN	Isanti	urban	333	28.5%	435.79	0.765	29.3%
27061	MN	Itasca	rural	877	28.5%	2,667.72	0.329	42.8%
27063	MN	Jackson	rural	271	28.2%	702.98	0.385	54.4%
27065	MN	Kanabec	rural	217	28.4%	521.59	0.416	36.5%
27067	MN	Kandiyohi	rural	484	28.3%	796.78	0.608	29.7%

27071	MN	Koochiching	rural	254	28.2%	3,104.07	0.082	39.1%
27073	MN	Lac qui Parle	rural	111	28.2%	765.02	0.145	37.1%
27075	MN	Lake	rural	230	28.7%	2,109.29	0.109	44.0%
27079	MN	Le Sueur	urban	389	28.3%	448.76	0.866	31.9%
27081	MN	Lincoln	rural	153	28.3%	536.76	0.284	55.6%
27083	MN	Lyon	rural	491	28.4%	714.56	0.688	51.4%
27089	MN	Marshall	rural	159	28.1%	1,775.07	0.089	41.6%
27091	MN	Martin	rural	382	28.2%	712.35	0.536	40.0%
27085	MN	McLeod	rural	499	28.4%	491.47	1.016	29.9%
27093	MN	Meeker	rural	226	28.2%	608.18	0.372	25.0%
27095	MN	Mille Lacs	urban	384	28.4%	572.31	0.672	29.8%
27097	MN	Morrison	rural	395	28.1%	1,125.06	0.351	29.3%
27099	MN	Mower	rural	796	28.2%	711.33	1.119	50.6%
27101	MN	Murray	rural	230	28.2%	704.70	0.326	59.0%
27103	MN	Nicollet	urban	327	28.5%	448.49	0.730	34.1%
27105	MN	Nobles	rural	412	28.4%	715.11	0.577	60.6%
27107	MN	Norman	rural	132	28.4%	872.79	0.151	47.0%
27109	MN	Olmsted	urban	2,328	28.6%	653.35	3.563	49.1%
27111	MN	Otter Tail	rural	926	28.2%	1972.07	0.470	32.9%
27113	MN	Pennington	rural	169	28.5%	616.57	0.275	33.1%
27115	MN	Pine	rural	383	28.3%	1,411.29	0.271	32.3%
27117	MN	Pipestone	rural	254	28.5%	465.05	0.547	61.2%
27119	MN	Polk	urban	462	28.1%	1,971.13	0.234	40.3%
27121	MN	Pope	rural	200	28.7%	669.71	0.298	36.5%
27123	MN	Ramsey	urban	6,181	28.4%	152.21	40.610	27.5%
27127	MN	Redwood	rural	294	28.5%	878.57	0.334	41.6%
27129	MN	Renville	rural	280	28.5%	982.91	0.285	46.4%
27131	MN	Rice	rural	673	28.4%	495.68	1.357	30.8%
27133	MN	Rock	rural	212	28.1%	482.45	0.439	49.6%
27135	MN	Roseau	rural	134	27.9%	1,671.60	0.080	24.8%
27139	MN	Scott	urban	752	28.6%	356.48	2.110	27.8%
27141	MN	Sherburne	urban	464	28.5%	432.92	1.071	27.0%
27143	MN	Sibley	urban	188	28.7%	588.78	0.319	35.0%
27137	MN	St. Louis	urban	2,940	28.3%	6247.4	0.471	37.7%
27145	MN	Stearns	urban	1,807	28.4%	1,343.13	1.345	31.2%
27147	MN	Steele	rural	401	28.2%	429.65	0.933	30.3%
27149	MN	Stevens	rural	167	28.4%	563.60	0.296	49.0%
27151	MN	Swift	rural	196	28.2%	742.08	0.264	44.8%
27153	MN	Todd	rural	231	28.0%	944.98	0.245	25.1%
27157	MN	Wabasha	urban	385	28.6%	522.98	0.736	36.0%
27159	MN	Wadena	rural	215	28.1%	536.27	0.400	32.3%
27161	MN	Waseca	rural	228	28.0%	423.36	0.539	33.7%
27163	MN	Washington	urban	1,313	28.3%	384.28	3.416	26.8%
27165	MN	Watonwan	rural	222	28.4%	434.95	0.509	46.6%
27167	MN	Wilkin	rural	106	28.4%	750.96	0.141	41.0%
27169	MN	Winona	rural	733	28.5%	626.21	1.170	44.2%
27171	MN	Wright	urban	916	28.3%	661.46	1.385	26.3%
27173	MN	Yellow Medicine	rural	248	28.4%	759.1	0.326	51.3%
29001	MO	Adair	rural	595	28.6%	567.32	1.048	87.3%
29003	MO	Andrew	urban	346	28.6%	432.70	0.799	85.6%
29005	MO	Atchison	rural	232	28.6%	547.30	0.423	90.4%
29007	MO	Audrain	rural	617	28.4%	692.23	0.891	77.3%
29009	MO	Barry	rural	895	28.5%	778.25	1.150	56.2%
29011	MO	Barton	rural	289	28.5%	591.92	0.488	73.2%
29013	MO	Bates	urban	450	28.7%	836.69	0.537	74.9%
29015	MO	Benton	rural	765	28.7%	704.06	1.087	70.5%
29017	MO	Bollinger	urban	299	28.4%	617.91	0.483	78.7%
29019	MO	Boone	urban	3,010	28.6%	685.41	4.392	73.8%
29021	MO	Buchanan	urban	2,211	28.5%	408.03	5.418	83.2%
29023	MO	Butler	rural	1,076	28.7%	694.67	1.549	81.1%
29025	MO	Caldwell	urban	283	28.4%	426.39	0.664	79.0%
29027	MO	Callaway	urban	910	28.4%	834.57	1.090	74.8%
29029	MO	Camden	rural	1,454	28.7%	655.92	2.217	78.3%
29031	MO	Cape Girardeau	urban	1,987	28.7%	578.53	3.434	84.3%
29033	MO	Carroll	rural	308	28.6%	694.62	0.444	81.0%
29035	MO	Carter	rural	192	28.3%	507.36	0.378	79.6%
29037	MO	Cass	urban	1,444	28.5%	696.84	2.073	50.3%
29039	MO	Cedar	rural	397	28.6%	474.48	0.837	59.4%
29041	MO	Chariton	rural	242	28.5%	751.18	0.322	85.4%
29043	MO	Christian	urban	1,275	28.6%	562.65	2.266	46.5%
29045	MO	Clark	rural	191	28.7%	504.69	0.378	85.9%
29047	MO	Clay	urban	4,429	28.7%	397.30	11.147	64.7%
29049	MO	Clinton	urban	603	28.7%	418.96	1.438	80.0%
29051	MO	Cole	urban	1,871	28.6%	393.75	4.751	79.6%
29053	MO	Cooper	rural	433	28.6%	564.77	0.766	75.0%
29055	MO	Crawford	rural	439	28.3%	742.52	0.592	63.5%
29057	MO	Dade	rural	183	28.4%	490.01	0.373	52.8%
29059	MO	Dallas	urban	265	28.4%	540.77	0.490	44.1%
29061	MO	Daviess	rural	242	28.4%	563.24	0.430	84.2%
29063	MO	DeKalb	urban	236	28.6%	421.36	0.561	84.8%
29065	MO	Dent	rural	512	28.6%	752.79	0.681	84.6%
29067	MO	Douglas	rural	224	28.2%	813.63	0.275	43.3%

29069	MO	Dunklin	rural	807	28.6%	541.07	1.491	82.0%
29071	MO	Franklin	urban	1,591	28.6%	922.68	1.724	41.1%
29073	MO	Gasconade	rural	455	28.6%	517.80	0.878	66.6%
29075	MO	Gentry	rural	220	28.5%	491.42	0.447	89.1%
29077	MO	Greene	urban	4,725	28.6%	675.30	6.997	46.8%
29079	MO	Grundy	rural	378	28.7%	435.28	0.869	90.0%
29081	MO	Harrison	rural	334	28.4%	722.50	0.463	89.6%
29083	MO	Henry	rural	713	28.6%	696.95	1.023	70.5%
29085	MO	Hickory	rural	281	28.7%	399.09	0.703	56.5%
29087	MO	Holt	rural	172	28.1%	462.69	0.372	86.0%
29089	MO	Howard	rural	250	28.5%	463.85	0.538	76.3%
29091	MO	Howell	rural	1,203	28.6%	927.25	1.297	71.8%
29093	MO	Iron	rural	317	28.6%	550.26	0.575	82.2%
29095	MO	Jackson	urban	10,180	28.5%	604.46	16.841	48.9%
29097	MO	Jasper	urban	2,733	28.6%	638.49	4.281	67.0%
29099	MO	Jefferson	urban	2,674	28.6%	656.63	4.072	43.4%
29101	MO	Johnson	rural	876	28.6%	829.28	1.056	72.6%
29103	MO	Knox	rural	143	28.3%	504.01	0.284	89.7%
29105	MO	Laclede	rural	657	28.5%	764.72	0.859	45.8%
29107	MO	Lafayette	urban	793	28.6%	628.43	1.263	65.6%
29109	MO	Lawrence	rural	688	28.6%	611.74	1.125	53.6%
29111	MO	Lewis	rural	321	28.6%	505.04	0.637	85.0%
29113	MO	Lincoln	urban	720	28.6%	626.56	1.149	54.7%
29115	MO	Linn	rural	465	28.5%	615.56	0.756	85.2%
29117	MO	Livingston	rural	432	28.6%	532.33	0.812	83.8%
29121	MO	Macon	rural	538	28.5%	801.23	0.671	86.3%
29123	MO	Madison	rural	345	28.3%	494.39	0.697	79.2%
29125	MO	Maries	rural	195	28.9%	526.98	0.371	80.1%
29127	MO	Marion	rural	815	28.6%	436.92	1.864	80.2%
29119	MO	McDonald	urban	364	28.7%	539.48	0.676	57.3%
29129	MO	Mercer	rural	116	28.3%	453.84	0.256	86.7%
29131	MO	Miller	rural	791	28.5%	592.59	1.334	75.9%
29133	MO	Mississippi	rural	367	28.5%	411.58	0.891	87.8%
29135	MO	Moniteau	urban	346	28.6%	415.03	0.833	79.2%
29137	MO	Monroe	rural	328	28.3%	647.65	0.506	84.5%
29139	MO	Montgomery	rural	340	28.7%	536.25	0.634	74.9%
29141	MO	Morgan	rural	754	28.6%	597.63	1.261	79.0%
29143	MO	New Madrid	rural	383	28.7%	674.84	0.567	81.3%
29145	MO	Newton	urban	950	28.7%	624.76	1.521	67.5%
29147	MO	Nodaway	rural	593	28.7%	876.96	0.676	89.5%
29149	MO	Oregon	rural	326	28.5%	789.80	0.413	77.0%
29151	MO	Osage	urban	311	28.5%	604.35	0.514	80.8%
29153	MO	Ozark	rural	304	28.5%	744.97	0.408	61.9%
29155	MO	Pemiscot	rural	316	28.5%	492.54	0.642	74.2%
29157	MO	Perry	rural	504	28.5%	474.35	1.064	83.9%
29159	MO	Pettis	rural	1,069	28.6%	682.22	1.566	78.5%
29161	MO	Phelps	rural	1,072	28.6%	671.78	1.595	77.3%
29163	MO	Pike	rural	424	28.9%	670.44	0.632	76.7%
29165	MO	Platte	urban	1,622	28.5%	420.19	3.861	64.2%
29167	MO	Polk	urban	576	28.4%	635.52	0.906	46.0%
29169	MO	Pulaski	rural	740	28.5%	547.10	1.353	80.6%
29171	MO	Putnam	rural	187	28.4%	517.32	0.361	86.8%
29173	MO	Ralls	rural	229	28.4%	469.78	0.487	80.2%
29175	MO	Randolph	rural	597	28.4%	482.68	1.237	76.1%
29177	MO	Ray	urban	499	28.7%	568.81	0.877	77.4%
29179	MO	Reynolds	rural	176	28.6%	808.48	0.218	80.0%
29181	MO	Ripley	rural	360	28.7%	629.54	0.572	82.1%
29195	MO	Saline	rural	507	28.7%	755.50	0.671	73.0%
29197	MO	Schuyler	rural	169	28.9%	307.30	0.549	82.5%
29199	MO	Scotland	rural	139	28.6%	436.67	0.318	89.2%
29201	MO	Scott	rural	1,116	28.6%	419.99	2.657	82.4%
29203	MO	Shannon	rural	203	28.8%	1,003.82	0.203	75.5%
29205	MO	Shelby	rural	243	28.7%	500.86	0.485	87.9%
29183	MO	St. Charles	urban	5,487	28.7%	560.44	9.790	46.4%
29185	MO	St. Clair	rural	292	28.8%	669.98	0.436	69.3%
29187	MO	St. Francois	rural	1,422	28.7%	451.89	3.146	73.7%
29189	MO	St. Louis	urban	17,975	28.5%	507.8	35.397	48.7%
29510	MO	St. Louis City	urban	3,042	28.0%	61.91	49.143	45.3%
29186	MO	Ste. Genevieve	rural	439	28.6%	499.15	0.879	77.3%
29207	MO	Stoddard	rural	838	28.6%	823.22	1.018	77.7%
29209	MO	Stone	rural	914	28.7%	464.03	1.971	54.7%
29211	MO	Sullivan	rural	197	28.5%	647.98	0.304	86.6%
29213	MO	Taney	rural	1,275	28.7%	632.44	2.016	54.5%
29215	MO	Texas	rural	760	28.5%	1,177.27	0.645	81.9%
29217	MO	Vernon	rural	533	28.7%	826.40	0.645	74.3%
29219	MO	Warren	urban	476	28.5%	428.60	1.111	45.3%
29221	MO	Washington	rural	473	28.6%	759.91	0.623	72.3%
29223	MO	Wayne	rural	479	28.7%	759.18	0.631	77.6%
29225	MO	Webster	urban	611	28.3%	592.56	1.030	40.3%
29229	MO	Wright	rural	444	28.4%	681.77	0.651	53.1%
28001	MS	Adams	rural	829	28.1%	462.41	1.792	78.9%
28003	MS	Alcorn	rural	1,051	28.9%	400.04	2.626	91.4%

28005	MS	Amite	rural	314	28.2%	730.10	0.430	82.5%
28007	MS	Attala	rural	499	28.2%	734.98	0.679	78.8%
28009	MS	Benton	urban	199	28.2%	406.62	0.490	77.6%
28011	MS	Bolivar	rural	725	28.4%	876.57	0.827	85.0%
28013	MS	Calhoun	rural	399	28.2%	586.57	0.681	87.7%
28015	MS	Carroll	rural	282	28.3%	628.24	0.449	88.8%
28017	MS	Chickasaw	rural	453	28.2%	501.78	0.902	81.7%
28019	MS	Choctaw	rural	186	28.2%	418.18	0.444	86.3%
28021	MS	Claiborne	rural	166	28.0%	487.41	0.341	73.0%
28023	MS	Clarke	rural	479	28.0%	691.55	0.693	85.1%
28025	MS	Clay	rural	484	28.2%	410.08	1.181	82.0%
28027	MS	Coahoma	rural	442	28.2%	552.44	0.800	83.5%
28029	MS	Copiah	urban	657	28.2%	777.24	0.846	68.9%
28031	MS	Covington	rural	471	28.3%	413.79	1.139	77.7%
28033	MS	DeSoto	urban	3,007	28.7%	476.15	6.316	72.6%
28035	MS	Forrest	urban	2,009	28.5%	466.31	4.308	74.3%
28037	MS	Franklin	rural	198	28.1%	563.78	0.351	87.0%
28039	MS	George	rural	552	28.4%	478.71	1.154	71.7%
28041	MS	Greene	rural	200	27.9%	712.76	0.281	84.7%
28043	MS	Grenada	rural	578	28.3%	422.11	1.370	83.9%
28045	MS	Hancock	urban	817	28.7%	473.75	1.724	61.5%
28047	MS	Harrison	urban	4,115	28.6%	573.99	7.169	73.1%
28049	MS	Hinds	urban	3,988	28.4%	869.74	4.585	64.4%
28051	MS	Holmes	rural	356	27.8%	756.70	0.470	80.8%
28053	MS	Humphreys	rural	198	27.6%	418.49	0.473	85.6%
28057	MS	Itawamba	rural	511	28.8%	532.79	0.958	90.4%
28059	MS	Jackson	urban	2,496	28.6%	722.75	3.453	68.2%
28061	MS	Jasper	rural	404	28.0%	676.24	0.598	81.9%
28063	MS	Jefferson	rural	124	27.3%	519.93	0.239	81.1%
28065	MS	Jefferson Davis	rural	268	28.5%	408.44	0.655	85.9%
28067	MS	Jones	rural	1,557	28.4%	694.80	2.241	79.6%
28069	MS	Kemper	rural	213	27.6%	766.18	0.278	84.0%
28071	MS	Lafayette	rural	840	28.5%	631.71	1.330	84.5%
28073	MS	Lamar	urban	644	28.6%	497.06	1.296	73.5%
28075	MS	Lauderdale	rural	1,926	28.3%	703.63	2.737	84.8%
28077	MS	Lawrence	rural	402	28.6%	430.67	0.934	84.0%
28079	MS	Leake	rural	486	28.2%	583.00	0.834	76.7%
28081	MS	Lee	rural	2,209	28.7%	449.95	4.910	86.1%
28083	MS	Leflore	rural	661	28.2%	592.54	1.116	88.3%
28085	MS	Lincoln	rural	820	28.6%	586.11	1.399	82.4%
28087	MS	Lowndes	rural	1,357	28.2%	505.51	2.685	82.0%
28089	MS	Madison	urban	1,887	28.3%	714.51	2.641	70.8%
28091	MS	Marion	rural	597	28.3%	542.38	1.101	79.1%
28093	MS	Marshall	urban	826	28.2%	706.19	1.169	73.4%
28095	MS	Monroe	rural	917	28.5%	765.09	1.198	87.3%
28097	MS	Montgomery	rural	366	28.3%	406.98	0.898	91.1%
28099	MS	Neshoba	rural	527	28.3%	570.14	0.925	83.1%
28101	MS	Newton	rural	714	28.5%	578.10	1.235	84.7%
28103	MS	Noxubee	rural	255	27.8%	695.14	0.366	91.2%
28105	MS	Oktibbeha	rural	838	28.4%	458.20	1.829	82.8%
28107	MS	Panola	rural	730	28.6%	685.14	1.065	79.1%
28109	MS	Pearl River	rural	1,204	28.6%	810.86	1.485	66.3%
28111	MS	Perry	urban	274	28.5%	647.25	0.424	85.2%
28113	MS	Pike	rural	942	28.3%	409.01	2.304	75.9%
28115	MS	Pontotoc	rural	659	28.6%	497.69	1.324	85.5%
28117	MS	Prentiss	rural	766	28.6%	414.98	1.846	91.3%
28119	MS	Quitman	rural	141	28.1%	405.01	0.349	85.4%
28121	MS	Rankin	urban	2,831	28.6%	775.48	3.650	71.2%
28123	MS	Scott	rural	619	28.2%	609.18	1.017	76.0%
28125	MS	Sharkey	rural	102	28.0%	431.72	0.236	84.6%
28127	MS	Simpson	urban	623	28.5%	589.16	1.057	77.3%
28129	MS	Smith	rural	274	28.3%	636.25	0.431	77.9%
28131	MS	Stone	rural	431	28.5%	445.48	0.967	70.6%
28133	MS	Sunflower	rural	429	28.0%	697.75	0.614	86.6%
28135	MS	Tallahatchie	rural	255	28.3%	645.29	0.395	86.9%
28137	MS	Tate	urban	580	28.6%	404.76	1.433	74.7%
28139	MS	Tippah	rural	635	28.5%	457.81	1.386	89.1%
28141	MS	Tishomingo	rural	700	28.7%	424.25	1.650	92.0%
28143	MS	Tunica	urban	168	28.0%	454.67	0.370	74.8%
28145	MS	Union	rural	711	28.4%	415.60	1.712	87.2%
28147	MS	Walthall	rural	301	28.2%	403.94	0.745	82.9%
28149	MS	Warren	rural	1,078	28.4%	588.50	1.832	78.1%
28151	MS	Washington	rural	1,051	28.0%	724.74	1.450	78.0%
28153	MS	Wayne	rural	435	28.5%	810.75	0.537	86.4%
28155	MS	Webster	rural	314	28.3%	420.94	0.746	89.9%
28157	MS	Wilkinson	rural	182	27.9%	678.11	0.269	76.2%
28159	MS	Winston	rural	490	28.2%	607.24	0.808	81.5%
28161	MS	Yalobusha	rural	455	28.2%	467.13	0.973	84.1%
28163	MS	Yazoo	urban	537	28.2%	922.95	0.582	84.1%
30001	MT	Beaverhead	rural	337	28.4%	5,541.62	0.061	79.2%
30003	MT	Big Horn	rural	213	28.6%	4995.46	0.043	76.1%
30005	MT	Blaine	rural	151	28.7%	4,227.55	0.036	81.8%

30007	MT	Broadwater	rural	218	28.6%	1,192.54	0.183	70.5%
30009	MT	Carbon	urban	357	28.2%	2,048.79	0.174	70.7%
30013	MT	Cascade	urban	2,063	28.4%	2,698.16	0.764	66.5%
30015	MT	Chouteau	rural	162	28.2%	3,972.49	0.041	74.3%
30017	MT	Custer	rural	377	28.4%	3,783.36	0.100	83.6%
30021	MT	Dawson	rural	291	28.4%	2,371.86	0.123	92.0%
30023	MT	Deer Lodge	rural	350	28.5%	736.53	0.475	81.3%
30027	MT	Fergus	rural	415	28.2%	4,339.80	0.096	71.8%
30029	MT	Flathead	rural	3,002	28.5%	5,087.66	0.590	69.1%
30031	MT	Gallatin	rural	1,964	28.4%	2,602.69	0.754	69.8%
30035	MT	Glacier	rural	221	28.4%	2,995.94	0.074	75.2%
30039	MT	Granite	rural	120	28.4%	1,727.41	0.070	78.6%
30041	MT	Hill	rural	432	28.6%	2,898.95	0.149	85.8%
30043	MT	Jefferson	rural	359	28.4%	1,656.26	0.216	68.6%
30047	MT	Lake	rural	800	28.6%	1,490.15	0.537	72.6%
30049	MT	Lewis and Clark	rural	1,740	28.5%	3458.83	0.503	67.9%
30051	MT	Liberty	rural	103	28.3%	1,430.05	0.072	91.3%
30053	MT	Lincoln	rural	689	28.4%	3,612.92	0.191	66.3%
30057	MT	Madison	rural	351	28.1%	3,587.48	0.098	82.6%
30061	MT	Mineral	rural	203	28.4%	1,219.44	0.167	75.3%
30063	MT	Missoula	urban	2,673	28.6%	2,593.42	1.031	72.3%
30065	MT	Musselshell	rural	175	28.1%	1,868.16	0.094	77.5%
30067	MT	Park	rural	525	28.5%	2,803.06	0.187	72.3%
30071	MT	Phillips	rural	165	28.5%	5,140.04	0.032	87.7%
30073	MT	Pondera	rural	195	28.4%	1,622.86	0.120	74.9%
30077	MT	Powell	rural	213	28.1%	2,326.39	0.091	78.5%
30081	MT	Ravalli	rural	1,626	28.5%	2,390.82	0.680	70.0%
30083	MT	Richland	rural	299	28.7%	2,084.14	0.144	92.6%
30085	MT	Roosevelt	rural	235	28.6%	2,354.79	0.100	89.7%
30087	MT	Rosebud	rural	217	28.6%	5,010.40	0.043	76.9%
30089	MT	Sanders	rural	506	28.2%	2,760.52	0.183	73.7%
30091	MT	Sheridan	rural	170	28.4%	1,677.08	0.101	90.2%
30093	MT	Silver Bow	rural	1,013	28.5%	718.48	1.410	75.6%
30095	MT	Stillwater	rural	303	28.6%	1,795.35	0.169	68.5%
30097	MT	Sweet Grass	rural	139	28.2%	1855.2	0.075	80.1%
30099	MT	Teton	rural	179	28.4%	2,272.37	0.079	60.4%
30105	MT	Valley	rural	328	28.4%	4,925.82	0.067	90.4%
30111	MT	Yellowstone	urban	3,701	28.5%	2,633.29	1.405	68.1%
37001	NC	Alamance	urban	2,052	28.5%	423.94	4.839	37.5%
37003	NC	Alexander	urban	729	28.7%	259.99	2.805	59.9%
37005	NC	Alleghany	rural	319	28.5%	235.06	1.357	57.4%
37007	NC	Anson	rural	472	28.3%	531.45	0.889	69.6%
37009	NC	Ashe	rural	801	28.6%	426.13	1.880	65.2%
37011	NC	Avery	rural	578	28.7%	247.09	2.339	67.4%
37013	NC	Beaufort	rural	1,563	28.5%	827.19	1.890	77.6%
37015	NC	Bertie	rural	514	27.8%	699.27	0.734	82.3%
37017	NC	Bladen	rural	563	28.0%	874.33	0.644	65.3%
37019	NC	Brunswick	urban	4,986	28.7%	846.97	5.886	75.0%
37021	NC	Buncombe	urban	6,598	28.6%	656.67	10.048	65.9%
37023	NC	Burke	urban	1,627	28.6%	507.10	3.209	58.2%
37025	NC	Cabarrus	urban	2,930	28.5%	361.75	8.099	55.9%
37027	NC	Caldwell	urban	1,560	28.4%	471.57	3.309	55.6%
37029	NC	Camden	rural	186	28.2%	240.56	0.773	76.5%
37031	NC	Carteret	rural	1,914	28.6%	506.25	3.780	75.3%
37033	NC	Caswell	rural	336	27.9%	424.92	0.792	48.4%
37035	NC	Catawba	urban	3,329	28.6%	398.72	8.349	58.9%
37037	NC	Chatham	urban	1,038	28.5%	682.19	1.522	50.6%
37039	NC	Cherokee	rural	974	28.6%	455.43	2.139	68.5%
37041	NC	Chowan	rural	476	28.6%	172.47	2.759	82.2%
37043	NC	Clay	rural	448	28.5%	214.75	2.087	73.5%
37045	NC	Cleveland	rural	2,277	28.5%	464.25	4.905	68.0%
37047	NC	Columbus	rural	1,180	28.2%	937.29	1.259	76.7%
37049	NC	Craven	urban	2,963	28.5%	708.96	4.179	83.5%
37051	NC	Cumberland	urban	4,382	28.4%	652.31	6.718	67.5%
37053	NC	Currituck	urban	568	28.6%	261.85	2.170	79.5%
37055	NC	Dare	rural	1,185	28.7%	383.42	3.091	83.2%
37057	NC	Davidson	urban	1,723	28.4%	552.67	3.118	35.3%
37059	NC	Davie	urban	676	28.7%	264.11	2.559	38.9%
37061	NC	Duplin	rural	950	28.2%	816.22	1.164	74.2%
37063	NC	Durham	urban	4,080	28.4%	285.98	14.268	58.8%
37065	NC	Edgecombe	urban	804	28.0%	505.34	1.590	73.4%
37067	NC	Forsyth	urban	4,537	28.4%	408.15	11.116	34.8%
37069	NC	Franklin	urban	930	28.3%	491.68	1.892	60.0%
37071	NC	Gaston	urban	3,504	28.6%	356.03	9.843	54.4%
37073	NC	Gates	urban	244	28.2%	340.44	0.718	74.4%
37075	NC	Graham	rural	231	28.3%	292.08	0.790	70.0%
37077	NC	Granville	rural	833	28.2%	531.57	1.567	58.3%
37079	NC	Greene	rural	301	28.2%	265.93	1.131	77.8%
37081	NC	Guilford	urban	6,177	28.4%	645.70	9.567	38.3%
37083	NC	Halifax	rural	1,236	27.9%	724.09	1.707	70.2%
37085	NC	Harnett	rural	1,353	28.2%	594.99	2.274	68.0%
37087	NC	Haywood	urban	1,796	28.7%	553.69	3.243	62.9%

37089	NC	Henderson	urban	4,182	28.8%	373.07	11.210	68.2%
37091	NC	Hertford	rural	500	27.8%	353.06	1.415	79.4%
37093	NC	Hoke	urban	339	28.3%	390.74	0.868	63.1%
37095	NC	Hyde	rural	111	28.7%	612.70	0.182	74.1%
37097	NC	Iredell	urban	2,853	28.5%	573.83	4.972	57.3%
37099	NC	Jackson	rural	931	28.7%	490.75	1.897	67.0%
37101	NC	Johnston	urban	2,386	28.5%	791.30	3.016	66.8%
37103	NC	Jones	urban	245	28.3%	470.71	0.522	77.5%
37105	NC	Lee	rural	1,441	28.6%	254.96	5.652	68.4%
37107	NC	Lenoir	rural	1,519	28.4%	400.59	3.793	78.3%
37109	NC	Lincoln	urban	1,612	28.5%	297.94	5.411	64.7%
37113	NC	Macon	rural	1,564	28.8%	515.56	3.034	80.4%
37115	NC	Madison	urban	589	28.6%	449.57	1.310	66.4%
37117	NC	Martin	rural	611	28.1%	461.22	1.326	78.5%
37111	NC	McDowell	rural	1,015	28.4%	440.61	2.303	60.0%
37119	NC	Mecklenburg	urban	13,349	28.5%	523.84	25.484	57.9%
37121	NC	Mitchell	rural	457	28.5%	221.42	2.065	66.0%
37123	NC	Montgomery	rural	528	28.4%	491.76	1.074	65.4%
37125	NC	Moore	rural	3,055	28.6%	697.84	4.377	68.0%
37127	NC	Nash	urban	2,349	28.3%	540.41	4.347	71.5%
37129	NC	New Hanover	urban	5,240	28.6%	191.53	27.356	75.1%
37131	NC	Northampton	rural	512	27.8%	536.59	0.954	74.8%
37133	NC	Onslow	urban	2,381	28.5%	762.74	3.121	78.9%
37135	NC	Orange	urban	2,472	28.5%	397.96	6.213	55.5%
37137	NC	Pamlico	urban	454	28.3%	336.54	1.349	82.5%
37139	NC	Pasquotank	rural	892	28.3%	226.88	3.931	77.6%
37141	NC	Pender	urban	1,271	28.4%	869.79	1.462	74.7%
37143	NC	Perquimans	rural	481	28.6%	247.09	1.948	81.2%
37145	NC	Person	urban	654	28.4%	392.32	1.668	54.5%
37147	NC	Pitt	urban	2,863	28.4%	651.97	4.392	73.9%
37149	NC	Polk	rural	841	28.6%	237.79	3.536	73.2%
37151	NC	Randolph	urban	1,823	28.6%	782.52	2.330	36.4%
37153	NC	Richmond	rural	946	28.4%	473.82	1.997	75.1%
37155	NC	Robeson	rural	2,062	28.4%	949.22	2.172	66.4%
37157	NC	Rockingham	urban	1,281	28.3%	565.55	2.266	37.5%
37159	NC	Rowan	urban	2,050	28.4%	511.37	4.009	49.2%
37161	NC	Rutherford	rural	1,711	28.4%	564.15	3.033	71.9%
37163	NC	Sampson	rural	1,012	28.4%	944.74	1.071	69.1%
37165	NC	Scotland	rural	662	28.4%	318.84	2.075	70.0%
37167	NC	Stanly	rural	1,331	28.5%	395.09	3.369	63.5%
37169	NC	Stokes	urban	507	28.3%	448.86	1.130	29.3%
37171	NC	Surry	rural	1,199	28.5%	532.17	2.253	39.5%
37173	NC	Swain	rural	407	28.7%	528.00	0.772	76.2%
37175	NC	Transylvania	rural	1,327	28.9%	378.53	3.506	68.9%
37179	NC	Union	urban	2,686	28.5%	631.52	4.253	63.3%
37181	NC	Vance	rural	833	28.0%	253.52	3.286	56.6%
37183	NC	Wake	urban	13,556	28.5%	835.22	16.230	57.8%
37185	NC	Warren	rural	361	27.6%	428.46	0.843	61.4%
37187	NC	Washington	rural	345	28.3%	348.13	0.991	80.8%
37189	NC	Watauga	rural	898	28.7%	312.56	2.873	62.3%
37191	NC	Wayne	urban	2,238	28.5%	553.09	4.046	71.3%
37193	NC	Wilkes	rural	1,202	28.4%	754.28	1.594	48.0%
37195	NC	Wilson	rural	1,659	28.3%	368.17	4.506	75.8%
37197	NC	Yadkin	urban	477	28.2%	334.83	1.423	31.6%
37199	NC	Yancey	rural	583	28.6%	312.60	1.866	64.9%
38001	ND	Adams	rural	101	28.2%	987.62	0.102	85.0%
38003	ND	Barnes	rural	350	28.4%	1,491.55	0.235	73.4%
38005	ND	Benson	rural	136	28.4%	1,388.71	0.098	79.9%
38009	ND	Bottineau	rural	288	28.6%	1,668.42	0.173	90.2%
38011	ND	Bowman	rural	112	28.5%	1,161.81	0.096	83.4%
38015	ND	Burleigh	urban	2,011	28.5%	1,632.65	1.232	68.9%
38017	ND	Cass	urban	2,651	28.4%	1,764.94	1.502	66.5%
38019	ND	Cavalier	rural	169	28.3%	1,488.75	0.114	83.1%
38021	ND	Dickey	rural	173	28.4%	1,131.47	0.153	81.7%
38029	ND	Emmons	rural	113	28.5%	1,510.43	0.075	64.4%
38031	ND	Foster	rural	113	28.3%	635.45	0.178	76.9%
38035	ND	Grand Forks	urban	1,269	28.4%	1436.38	0.883	72.8%
38041	ND	Hettinger	rural	106	28.3%	1,132.22	0.093	78.9%
38045	ND	LaMoure	rural	145	28.5%	1,145.94	0.127	71.5%
38049	ND	McHenry	rural	183	28.5%	1,873.95	0.098	76.8%
38051	ND	McIntosh	rural	115	28.4%	974.73	0.118	66.6%
38053	ND	McKenzie	rural	145	28.6%	2,760.32	0.053	88.2%
38055	ND	McLean	rural	338	28.4%	2,110.88	0.160	77.5%
38057	ND	Mercer	rural	248	28.4%	1,042.96	0.237	77.3%
38059	ND	Morton	urban	675	28.6%	1,926.27	0.350	67.5%
38061	ND	Mountrail	rural	188	28.2%	1,825.30	0.103	86.5%
38063	ND	Nelson	rural	185	28.3%	981.77	0.189	92.8%
38067	ND	Pembina	rural	251	28.2%	1,118.69	0.224	78.0%
38069	ND	Pierce	rural	111	28.6%	1,018.60	0.109	62.9%
38071	ND	Ramsey	rural	416	28.4%	1,186.85	0.350	89.8%
38073	ND	Ransom	rural	171	28.2%	862.36	0.198	81.3%
38077	ND	Richland	rural	372	28.4%	1,435.78	0.259	72.3%

38079	ND	Rolette	rural	215	28.6%	903.08	0.238	74.7%
38081	ND	Sargent	rural	156	28.4%	858.51	0.181	83.5%
38089	ND	Stark	rural	634	28.5%	1,334.74	0.475	78.1%
38093	ND	Stutsman	rural	500	28.6%	2,221.72	0.225	60.9%
38097	ND	Traill	rural	250	28.5%	861.95	0.290	78.3%
38099	ND	Walsh	rural	363	28.2%	1,281.93	0.283	79.3%
38101	ND	Ward	rural	1,226	28.5%	2,013.28	0.609	78.0%
38103	ND	Wells	rural	218	28.5%	1,271.05	0.172	95.4%
38105	ND	Williams	rural	584	28.4%	2,077.40	0.281	89.8%
31001	NE	Adams	rural	931	28.6%	563.27	1.653	87.4%
31003	NE	Antelope	rural	244	28.6%	857.22	0.284	92.0%
31011	NE	Boone	rural	222	28.3%	686.55	0.324	91.3%
31013	NE	Box Butte	rural	339	28.5%	1075.29	0.315	89.5%
31015	NE	Boyd	rural	114	28.1%	539.94	0.211	95.3%
31017	NE	Brown	rural	138	28.7%	1,221.33	0.113	93.2%
31019	NE	Buffalo	rural	1,166	28.6%	968.11	1.204	82.4%
31021	NE	Burt	rural	271	28.5%	491.58	0.551	84.0%
31023	NE	Butler	rural	310	28.6%	584.91	0.529	88.5%
31025	NE	Cass	urban	682	28.5%	557.45	1.224	77.2%
31027	NE	Cedar	rural	262	28.6%	740.31	0.354	72.3%
31029	NE	Chase	rural	167	28.8%	894.42	0.187	95.1%
31031	NE	Cherry	rural	231	28.6%	5,960.42	0.039	93.4%
31033	NE	Cheyenne	rural	304	28.3%	1,196.29	0.254	86.1%
31035	NE	Clay	rural	267	28.3%	572.29	0.466	91.0%
31037	NE	Colfax	rural	365	28.2%	411.66	0.885	88.9%
31039	NE	Cuming	rural	343	28.7%	570.62	0.601	88.3%
31041	NE	Custer	rural	436	28.5%	2,575.52	0.169	92.4%
31043	NE	Dakota	urban	214	28.1%	264.25	0.811	59.7%
31045	NE	Dawes	rural	264	28.4%	1,396.46	0.189	75.4%
31047	NE	Dawson	rural	603	28.4%	1,013.10	0.596	80.9%
31051	NE	Dixon	urban	286	28.6%	476.23	0.600	64.7%
31053	NE	Dodge	rural	1,190	28.5%	528.71	2.251	81.6%
31055	NE	Douglas	urban	9,755	28.5%	328.45	29.702	65.0%
31059	NE	Fillmore	rural	256	28.3%	575.37	0.446	94.5%
31061	NE	Franklin	rural	156	28.4%	575.82	0.270	93.7%
31065	NE	Furnas	rural	231	28.4%	719.13	0.322	92.3%
31067	NE	Gage	rural	765	28.5%	851.49	0.898	84.7%
31069	NE	Garden	rural	123	28.5%	1,704.28	0.072	92.7%
31071	NE	Garfield	rural	109	28.3%	569.79	0.192	92.1%
31077	NE	Greeley	rural	117	28.9%	569.81	0.205	94.0%
31079	NE	Hall	urban	1,606	28.4%	546.29	2.941	84.2%
31081	NE	Hamilton	urban	328	28.7%	542.88	0.603	88.2%
31083	NE	Harlan	rural	135	28.6%	553.47	0.243	93.1%
31087	NE	Hitchcock	rural	141	28.5%	709.94	0.198	93.6%
31089	NE	Holt	rural	401	28.4%	2,412.40	0.166	93.5%
31093	NE	Howard	urban	235	28.1%	569.34	0.412	90.7%
31095	NE	Jefferson	rural	306	28.6%	570.18	0.537	87.2%
31097	NE	Johnson	rural	172	28.5%	376.05	0.457	89.1%
31099	NE	Kearney	rural	206	28.5%	516.24	0.400	88.8%
31101	NE	Keith	rural	348	28.7%	1,061.60	0.328	88.0%
31105	NE	Kimball	rural	157	28.6%	951.85	0.165	91.3%
31107	NE	Knox	rural	361	28.6%	1,108.35	0.326	83.4%
31109	NE	Lancaster	urban	6,452	28.5%	837.55	7.704	78.3%
31111	NE	Lincoln	rural	956	28.6%	2,564.07	0.373	77.3%
31119	NE	Madison	rural	1,016	28.6%	572.74	1.774	77.7%
31121	NE	Merrick	urban	244	28.9%	484.88	0.504	85.4%
31123	NE	Morrill	rural	177	28.2%	1,423.84	0.125	86.4%
31125	NE	Nance	rural	125	28.2%	441.63	0.284	88.6%
31127	NE	Nemaha	rural	227	28.6%	407.38	0.558	90.5%
31129	NE	Nuckolls	rural	198	28.4%	575.16	0.344	90.3%
31131	NE	Otoe	rural	477	28.4%	615.63	0.775	83.7%
31133	NE	Pawnee	rural	126	28.5%	431.07	0.292	92.5%
31135	NE	Perkins	rural	120	28.3%	883.34	0.136	91.6%
31137	NE	Phelps	rural	333	28.9%	539.79	0.616	92.8%
31139	NE	Pierce	rural	229	28.4%	573.25	0.399	90.6%
31141	NE	Platte	rural	906	28.6%	674.06	1.345	86.1%
31143	NE	Polk	rural	179	28.5%	438.34	0.408	92.4%
31145	NE	Red Willow	rural	414	28.6%	716.99	0.578	92.5%
31147	NE	Richardson	rural	344	28.8%	551.84	0.624	93.7%
31151	NE	Saline	rural	441	28.7%	574.02	0.769	89.8%
31153	NE	Sarpy	urban	2,536	28.6%	238.99	10.613	75.7%
31155	NE	Saunders	urban	582	28.6%	750.23	0.776	79.2%
31157	NE	Scotts Bluff	rural	1,189	28.6%	739.4	1.608	83.2%
31159	NE	Seward	urban	517	28.5%	571.43	0.904	87.6%
31161	NE	Sheridan	rural	217	28.3%	2,440.86	0.089	80.4%
31163	NE	Sherman	rural	124	28.3%	565.83	0.219	84.1%
31169	NE	Thayer	rural	255	28.8%	573.81	0.444	93.4%
31173	NE	Thurston	rural	138	28.8%	393.58	0.350	79.7%
31175	NE	Valley	rural	192	28.7%	568.05	0.339	93.5%
31177	NE	Washington	urban	512	28.4%	389.96	1.312	75.3%
31179	NE	Wayne	rural	207	28.3%	442.91	0.467	80.0%
31181	NE	Webster	rural	164	28.6%	574.91	0.286	92.3%

31185	NE	York	rural	520	28.6%	572.51	0.907	92.8%
33001	NH	Belknap	rural	2,359	28.5%	400.23	5.895	81.6%
33003	NH	Carroll	rural	2,177	28.6%	931.06	2.338	86.0%
33005	NH	Cheshire	rural	2,420	28.7%	706.66	3.424	80.7%
33007	NH	Coos	rural	1,266	28.7%	1,794.69	0.706	91.0%
33009	NH	Grafton	rural	3,010	28.7%	1,708.75	1.761	82.1%
33011	NH	Hillsborough	urban	9,377	28.7%	876.14	10.702	76.8%
33013	NH	Merrimack	rural	4,142	28.6%	934.12	4.434	80.5%
33015	NH	Rockingham	urban	8,223	28.7%	694.72	11.837	79.0%
33017	NH	Strafford	urban	2,988	28.6%	368.97	8.098	84.2%
33019	NH	Sullivan	rural	1,487	28.7%	537.31	2.767	87.3%
34001	NJ	Atlantic	urban	5,222	28.6%	555.70	9.397	75.5%
34003	NJ	Bergen	urban	17,493	28.6%	233.01	75.076	67.4%
34005	NJ	Burlington	urban	8,702	28.6%	798.58	10.897	66.3%
34007	NJ	Camden	urban	8,825	28.6%	221.26	39.886	64.3%
34009	NJ	Cape May	urban	3,139	28.7%	251.42	12.485	78.6%
34011	NJ	Cumberland	urban	2,519	28.6%	483.70	5.208	70.7%
34013	NJ	Essex	urban	9,441	28.4%	126.21	74.807	60.5%
34015	NJ	Gloucester	urban	4,654	28.6%	322.01	14.451	65.7%
34017	NJ	Hudson	urban	5,797	28.5%	46.19	125.510	57.0%
34019	NJ	Hunterdon	urban	3,190	28.7%	427.82	7.456	77.2%
34021	NJ	Mercer	urban	6,223	28.5%	224.56	27.712	68.0%
34023	NJ	Middlesex	urban	11,967	28.6%	308.91	38.740	65.4%
34025	NJ	Monmouth	urban	13,370	28.6%	468.79	28.520	71.7%
34027	NJ	Morris	urban	10,706	28.6%	460.18	23.265	73.8%
34029	NJ	Ocean	urban	14,687	28.8%	628.78	23.357	69.0%
34031	NJ	Passaic	urban	7,507	28.7%	184.59	40.670	64.6%
34033	NJ	Salem	urban	1,386	28.5%	331.90	4.177	75.9%
34035	NJ	Somerset	urban	6,176	28.7%	301.81	20.462	74.9%
34037	NJ	Sussex	urban	3,353	28.7%	519.01	6.459	76.2%
34039	NJ	Union	urban	8,171	28.5%	102.85	79.442	63.9%
34041	NJ	Warren	urban	2,582	28.6%	356.92	7.235	77.8%
35001	NM	Bernalillo	urban	8,152	28.6%	1,160.83	7.023	35.3%
35003	NM	Catron	rural	209	28.7%	6,923.69	0.030	73.8%
35005	NM	Chaves	rural	1,408	28.3%	6,065.27	0.232	79.9%
35006	NM	Cibola	rural	353	28.5%	4,539.48	0.078	64.1%
35007	NM	Colfax	rural	379	28.3%	3,758.06	0.101	72.0%
35009	NM	Curry	rural	935	28.4%	1,404.80	0.666	84.5%
35013	NM	Do+¦a Ana	urban	3,606	28.6%	3807.51	0.947	61.1%
35015	NM	Eddy	rural	1,183	28.5%	4,175.73	0.283	84.1%
35017	NM	Grant	rural	990	28.3%	3,961.63	0.250	70.8%
35023	NM	Hidalgo	rural	121	27.7%	3,436.86	0.035	70.7%
35025	NM	Lea	rural	1,177	28.3%	4,390.93	0.268	88.5%
35027	NM	Lincoln	rural	797	28.7%	4,831.09	0.165	71.5%
35028	NM	Los Alamos	rural	558	28.8%	109.17	5.113	81.4%
35029	NM	Luna	rural	655	28.3%	2,965.19	0.221	60.6%
35031	NM	McKinley	rural	958	28.2%	5,449.81	0.176	69.1%
35033	NM	Mora	rural	117	27.3%	1,931.27	0.061	67.2%
35035	NM	Otero	rural	1,345	28.5%	6,613.21	0.203	73.8%
35037	NM	Quay	rural	309	28.4%	2,874.35	0.107	76.1%
35039	NM	Rio Arriba	rural	739	28.2%	5860.84	0.126	58.3%
35041	NM	Roosevelt	rural	386	28.5%	2,447.43	0.158	83.3%
35045	NM	San Juan	urban	2,531	28.5%	5513.07	0.459	83.9%
35047	NM	San Miguel	rural	592	28.3%	4715.82	0.125	67.0%
35043	NM	Sandoval	urban	1,615	28.7%	3,710.65	0.435	38.5%
35049	NM	Santa Fe	urban	4,122	28.7%	1909.41	2.159	59.3%
35051	NM	Sierra	rural	483	28.5%	4,178.96	0.116	63.3%
35053	NM	Socorro	rural	296	28.3%	6,646.68	0.045	57.5%
35055	NM	Taos	rural	1,059	28.6%	2,203.11	0.481	68.0%
35057	NM	Torrance	urban	206	28.4%	3,344.85	0.062	39.5%
35059	NM	Union	rural	143	28.4%	3,823.74	0.037	88.0%
35061	NM	Valencia	urban	879	28.6%	1,066.17	0.824	33.8%
32510	NV	Carson City	urban	2,235	28.6%	144.66	15.452	78.9%
32001	NV	Churchill	rural	748	28.4%	4,930.46	0.152	81.4%
32003	NV	Clark	urban	25,149	28.6%	7,891.43	3.187	44.3%
32005	NV	Douglas	rural	1,703	28.7%	709.72	2.400	82.1%
32007	NV	Elko	rural	841	28.5%	17,169.83	0.049	85.0%
32013	NV	Humboldt	rural	404	28.2%	9,640.76	0.042	86.2%
32015	NV	Lander	rural	133	28.5%	5,490.10	0.024	85.0%
32017	NV	Lincoln	rural	256	28.5%	10,633.20	0.024	70.6%
32019	NV	Lyon	rural	1,640	28.5%	2,001.19	0.819	67.9%
32021	NV	Mineral	rural	157	28.3%	3,752.84	0.042	74.0%
32023	NV	Nye	rural	1,232	28.5%	18,181.92	0.068	41.3%
32027	NV	Pershing	rural	111	28.3%	6,036.56	0.018	84.2%
32031	NV	Washoe	urban	9,101	28.5%	6,302.37	1.444	57.0%
32033	NV	White Pine	rural	279	28.5%	8875.65	0.031	89.2%
36001	NY	Albany	urban	3,801	28.4%	522.80	7.270	41.1%
36003	NY	Allegany	rural	744	28.4%	1,029.31	0.723	46.8%
36005	NY	Bronx	urban	8,610	28.6%	42.10	204.506	31.0%
36007	NY	Broome	urban	3,729	28.5%	705.77	5.283	50.6%
36009	NY	Cattaraugus	rural	1,122	28.3%	1,308.35	0.858	37.4%
36011	NY	Cayuga	rural	1,541	28.4%	691.58	2.228	60.7%

36013	NY	Chautauqua	rural	2,126	28.5%	1,060.23	2.005	39.8%
36015	NY	Chemung	urban	1,617	28.4%	407.35	3.970	53.2%
36017	NY	Chenango	rural	983	28.5%	893.55	1.100	51.1%
36019	NY	Clinton	rural	1,622	28.5%	1,037.85	1.562	69.2%
36021	NY	Columbia	rural	1,459	28.5%	634.71	2.298	57.3%
36023	NY	Cortland	rural	920	28.5%	498.76	1.845	61.7%
36025	NY	Delaware	rural	1,106	28.5%	1,442.44	0.767	60.9%
36027	NY	Dutchess	urban	5,767	28.7%	795.63	7.248	67.9%
36029	NY	Erie	urban	8,891	28.3%	1,042.69	8.527	25.3%
36031	NY	Essex	rural	1,092	28.4%	1,794.23	0.609	71.0%
36033	NY	Franklin	rural	852	28.5%	1,629.12	0.523	62.5%
36035	NY	Fulton	rural	681	28.4%	495.47	1.375	37.4%
36037	NY	Genesee	rural	633	28.2%	492.94	1.284	27.0%
36039	NY	Greene	rural	971	28.4%	647.16	1.501	53.0%
36041	NY	Hamilton	rural	187	28.7%	1,717.37	0.109	63.4%
36043	NY	Herkimer	urban	1,113	28.5%	1,411.47	0.789	49.6%
36045	NY	Jefferson	urban	1,819	28.5%	1,268.59	1.434	60.9%
36047	NY	Kings	urban	17,732	28.4%	70.82	250.387	36.0%
36049	NY	Lewis	rural	512	28.5%	1,274.68	0.401	61.6%
36051	NY	Livingston	urban	590	28.2%	631.76	0.933	24.5%
36053	NY	Madison	urban	1,262	28.7%	654.84	1.927	51.5%
36055	NY	Monroe	urban	4,910	28.3%	657.21	7.471	17.8%
36057	NY	Montgomery	rural	888	28.2%	403.04	2.202	44.3%
36059	NY	Nassau	urban	20,314	28.6%	284.72	71.349	53.3%
36061	NY	New York	urban	22,299	28.8%	22.83	976.758	48.7%
36063	NY	Niagara	urban	2,410	28.3%	522.36	4.613	29.7%
36065	NY	Oneida	urban	4,209	28.5%	1,212.43	3.471	52.0%
36067	NY	Onondaga	urban	7,872	28.5%	778.39	10.114	51.1%
36069	NY	Ontario	urban	1,119	28.4%	644.07	1.738	24.3%
36071	NY	Orange	urban	5,548	28.6%	811.69	6.835	67.7%
36073	NY	Orleans	urban	407	28.3%	391.26	1.041	29.0%
36075	NY	Oswego	urban	1,952	28.5%	951.65	2.051	51.8%
36077	NY	Otsego	rural	1,598	28.5%	1,001.70	1.596	65.5%
36079	NY	Putnam	urban	1,801	28.7%	230.31	7.822	65.7%
36081	NY	Queens	urban	17,974	28.5%	108.53	165.616	33.5%
36083	NY	Rensselaer	urban	2,143	28.5%	652.43	3.285	41.1%
36085	NY	Richmond	urban	4,540	28.7%	58.37	77.786	36.5%
36087	NY	Rockland	urban	5,214	28.7%	173.55	30.045	62.8%
36091	NY	Saratoga	urban	3,493	28.5%	809.98	4.312	43.3%
36093	NY	Schenectady	urban	2,494	28.4%	204.52	12.196	41.0%
36095	NY	Schoharie	urban	705	28.4%	621.82	1.134	60.2%
36097	NY	Schuyler	rural	349	28.4%	328.33	1.063	54.0%
36099	NY	Seneca	rural	449	28.4%	323.71	1.388	40.6%
36089	NY	St. Lawrence	rural	2,315	28.5%	2680.38	0.864	68.5%
36101	NY	Steuben	rural	1,831	28.6%	1,390.56	1.317	53.8%
36103	NY	Suffolk	urban	23,417	28.7%	912.05	25.675	58.8%
36105	NY	Sullivan	rural	1,805	28.6%	968.13	1.865	77.1%
36107	NY	Tioga	urban	930	28.4%	518.60	1.794	51.7%
36109	NY	Tompkins	urban	1,857	28.5%	474.65	3.912	62.9%
36111	NY	Ulster	urban	3,794	28.6%	1,124.24	3.375	63.0%
36113	NY	Warren	urban	1,418	28.5%	866.95	1.635	48.1%
36115	NY	Washington	urban	1,101	28.4%	831.18	1.325	48.2%
36117	NY	Wayne	urban	846	28.3%	603.83	1.401	23.2%
36119	NY	Westchester	urban	16,186	28.6%	430.50	37.599	57.2%
36121	NY	Wyoming	rural	403	28.4%	592.75	0.680	28.1%
36123	NY	Yates	urban	357	28.6%	338.14	1.056	32.6%
39001	OH	Adams	rural	523	28.6%	583.87	0.896	52.2%
39003	OH	Allen	urban	2,008	28.3%	402.50	4.989	62.1%
39005	OH	Ashland	rural	887	28.6%	422.95	2.096	48.4%
39007	OH	Ashtabula	rural	2,028	28.4%	701.93	2.890	59.3%
39009	OH	Athens	rural	797	28.4%	503.60	1.582	46.9%
39011	OH	Auglaize	rural	1,263	28.5%	401.39	3.145	64.0%
39013	OH	Belmont	urban	986	28.3%	532.13	1.852	37.1%
39015	OH	Brown	urban	591	28.6%	490.02	1.206	44.8%
39017	OH	Butler	urban	4,625	28.5%	467.06	9.902	44.7%
39019	OH	Carroll	urban	332	28.6%	394.61	0.842	39.4%
39021	OH	Champaign	rural	566	28.4%	428.67	1.319	42.7%
39023	OH	Clark	urban	1,984	28.4%	397.47	4.991	37.9%
39025	OH	Clermont	urban	2,042	28.5%	452.10	4.517	41.4%
39027	OH	Clinton	rural	737	28.4%	408.68	1.803	49.3%
39029	OH	Columbiana	rural	1,890	28.5%	531.89	3.554	46.7%
39031	OH	Coshocton	rural	653	28.6%	563.91	1.159	59.5%
39033	OH	Crawford	rural	1,005	28.5%	401.79	2.501	60.6%
39035	OH	Cuyahoga	urban	19,128	28.3%	457.19	41.838	44.4%
39037	OH	Darke	rural	1,155	28.6%	598.10	1.932	62.1%
39039	OH	Defiance	rural	884	28.5%	411.46	2.149	65.7%
39041	OH	Delaware	urban	1,712	28.7%	443.10	3.863	42.7%
39043	OH	Erie	rural	1,999	28.6%	251.56	7.947	63.9%
39045	OH	Fairfield	urban	1,640	28.6%	504.41	3.252	36.2%
39047	OH	Fayette	rural	376	28.5%	406.36	0.926	42.0%
39049	OH	Franklin	urban	11,683	28.4%	532.19	21.953	38.3%
39051	OH	Fulton	urban	878	28.5%	405.44	2.167	57.4%

39053	OH	Gallia	rural	632	28.6%	466.53	1.354	61.0%
39055	OH	Geauga	urban	1,741	28.7%	400.16	4.351	50.7%
39057	OH	Greene	urban	1,798	28.4%	413.73	4.346	46.6%
39059	OH	Guernsey	rural	761	28.6%	522.25	1.458	54.2%
39061	OH	Hamilton	urban	12,371	28.4%	405.91	30.477	46.2%
39063	OH	Hancock	rural	1,386	28.6%	531.36	2.608	61.0%
39065	OH	Hardin	rural	595	28.5%	470.40	1.266	64.0%
39067	OH	Harrison	rural	338	28.5%	402.34	0.841	52.4%
39069	OH	Henry	rural	637	28.4%	416.01	1.532	64.8%
39071	OH	Highland	rural	688	28.7%	553.08	1.244	54.3%
39073	OH	Hocking	urban	469	28.5%	421.32	1.114	55.6%
39075	OH	Holmes	rural	331	28.3%	422.53	0.785	42.8%
39077	OH	Huron	rural	1,493	28.6%	491.49	3.037	67.0%
39079	OH	Jackson	rural	558	28.5%	420.30	1.327	59.0%
39081	OH	Jefferson	urban	1,357	28.4%	408.33	3.324	49.8%
39083	OH	Knox	rural	1,075	28.5%	525.49	2.045	51.9%
39085	OH	Lake	urban	4,229	28.6%	227.49	18.588	46.6%
39087	OH	Lawrence	urban	1,271	28.5%	453.37	2.803	65.0%
39089	OH	Licking	urban	2,205	28.5%	682.50	3.231	41.7%
39091	OH	Logan	rural	1,005	28.4%	458.43	2.193	59.8%
39093	OH	Lorain	urban	5,303	28.6%	491.10	10.799	52.5%
39095	OH	Lucas	urban	5,169	28.4%	340.86	15.165	42.0%
39097	OH	Madison	urban	549	28.4%	465.88	1.179	36.5%
39099	OH	Mahoning	urban	3,329	28.3%	411.62	8.088	36.7%
39101	OH	Marion	rural	1,084	28.4%	403.76	2.684	50.9%
39103	OH	Medina	urban	2,836	28.6%	421.36	6.731	45.6%
39105	OH	Meigs	rural	431	28.4%	430.10	1.003	62.2%
39107	OH	Mercer	rural	868	28.4%	462.45	1.876	62.5%
39109	OH	Miami	urban	1,910	28.5%	406.58	4.697	48.2%
39111	OH	Monroe	rural	260	28.3%	455.72	0.570	41.1%
39113	OH	Montgomery	urban	8,092	28.4%	461.55	17.531	40.2%
39115	OH	Morgan	rural	246	28.3%	416.42	0.591	52.4%
39117	OH	Morrow	urban	415	28.6%	406.08	1.021	50.5%
39119	OH	Muskingum	rural	1,654	28.5%	664.58	2.489	54.7%
39121	OH	Noble	rural	163	28.5%	398.01	0.410	52.9%
39123	OH	Ottawa	rural	1,109	28.5%	254.92	4.352	57.6%
39125	OH	Paulding	rural	350	28.3%	416.44	0.841	60.0%
39127	OH	Perry	urban	651	28.4%	407.97	1.595	53.6%
39129	OH	Pickaway	urban	660	28.2%	501.32	1.317	39.0%
39131	OH	Pike	rural	435	28.5%	440.28	0.989	58.1%
39133	OH	Portage	urban	1,962	28.5%	487.38	4.025	38.8%
39135	OH	Preble	rural	645	28.4%	424.12	1.520	45.6%
39137	OH	Putnam	rural	768	28.6%	482.52	1.591	67.8%
39139	OH	Richland	urban	2,824	28.6%	495.27	5.702	62.1%
39141	OH	Ross	rural	1,249	28.5%	689.19	1.813	47.8%
39143	OH	Sandusky	rural	1,046	28.5%	408.45	2.562	59.9%
39145	OH	Scioto	rural	1,422	28.5%	610.21	2.330	62.1%
39147	OH	Seneca	rural	1,371	28.6%	551.02	2.488	67.8%
39149	OH	Shelby	rural	847	28.4%	407.68	2.077	58.5%
39151	OH	Stark	urban	5,565	28.4%	575.27	9.675	35.0%
39153	OH	Summit	urban	6,703	28.3%	412.75	16.241	35.6%
39155	OH	Trumbull	urban	3,347	28.5%	618.30	5.413	40.8%
39157	OH	Tuscarawas	rural	1,535	28.5%	567.64	2.705	41.9%
39159	OH	Union	urban	482	28.5%	431.73	1.118	45.2%
39161	OH	Van Wert	rural	524	28.5%	409.16	1.281	61.9%
39163	OH	Vinton	rural	190	28.1%	412.36	0.461	55.3%
39165	OH	Warren	urban	2,632	28.6%	401.31	6.559	44.6%
39167	OH	Washington	rural	1,587	28.7%	631.97	2.510	67.4%
39169	OH	Wayne	rural	1,885	28.4%	554.93	3.397	44.5%
39171	OH	Williams	rural	775	28.7%	420.97	1.841	56.0%
39173	OH	Wood	urban	1,739	28.4%	617.20	2.817	45.2%
39175	OH	Wyandot	rural	501	28.4%	406.86	1.230	61.5%
40001	OK	Adair	rural	442	28.3%	573.48	0.771	81.6%
40003	OK	Alfalfa	rural	189	28.7%	866.45	0.218	91.1%
40005	OK	Atoka	rural	342	28.5%	975.52	0.350	86.8%
40007	OK	Beaver	rural	149	28.3%	1,814.67	0.082	86.7%
40009	OK	Beckham	rural	446	28.5%	901.81	0.495	86.8%
40011	OK	Blaine	rural	315	28.6%	928.42	0.340	87.7%
40013	OK	Bryan	rural	1,163	28.6%	904.47	1.286	84.9%
40015	OK	Caddo	rural	811	28.8%	1,278.29	0.634	85.9%
40017	OK	Canadian	urban	1,947	28.7%	896.63	2.171	61.8%
40019	OK	Carter	rural	1,399	28.6%	822.18	1.701	85.1%
40021	OK	Cherokee	rural	979	28.8%	749.41	1.307	78.5%
40023	OK	Choctaw	rural	453	28.6%	770.36	0.588	85.5%
40027	OK	Cleveland	urban	3,789	28.8%	538.77	7.033	70.7%
40029	OK	Coal	rural	160	28.2%	516.68	0.309	86.2%
40031	OK	Comanche	urban	2,218	28.5%	1,069.29	2.074	87.5%
40033	OK	Cotton	urban	169	29.0%	632.65	0.266	86.6%
40035	OK	Craig	rural	615	28.6%	761.35	0.807	81.6%
40037	OK	Creek	urban	1,310	28.5%	950.14	1.379	54.6%
40039	OK	Custer	rural	613	28.5%	988.82	0.620	85.7%
40041	OK	Delaware	rural	1,198	28.6%	738.18	1.623	78.0%

40043	OK	Dewey	rural	168	28.9%	999.48	0.169	86.4%
40045	OK	Ellis	rural	135	28.6%	1,231.52	0.110	84.2%
40047	OK	Garfield	rural	1,681	28.6%	1,058.47	1.588	85.8%
40049	OK	Garvin	rural	898	28.7%	802.12	1.119	84.1%
40051	OK	Grady	urban	1,083	28.7%	1,100.50	0.984	79.0%
40053	OK	Grant	rural	154	28.7%	1,000.87	0.153	89.6%
40055	OK	Greer	rural	183	28.7%	639.32	0.287	93.8%
40059	OK	Harper	rural	129	28.1%	1,039.02	0.125	89.5%
40061	OK	Haskell	rural	389	28.9%	576.52	0.674	82.7%
40063	OK	Hughes	rural	339	28.3%	804.65	0.421	79.9%
40065	OK	Jackson	rural	541	28.4%	802.65	0.674	89.0%
40067	OK	Jefferson	rural	203	28.7%	758.83	0.268	85.8%
40069	OK	Johnston	rural	295	28.9%	642.94	0.459	85.7%
40071	OK	Kay	rural	1,429	28.5%	919.73	1.553	83.8%
40073	OK	Kingfisher	rural	403	28.6%	898.16	0.449	84.0%
40075	OK	Kiowa	rural	267	28.5%	1,015.23	0.263	85.4%
40077	OK	Latimer	rural	225	28.7%	722.08	0.312	84.2%
40079	OK	Le Flore	urban	1,200	28.5%	1589.21	0.755	77.4%
40081	OK	Lincoln	urban	794	28.7%	952.31	0.834	73.2%
40083	OK	Logan	urban	661	28.5%	743.83	0.889	70.0%
40085	OK	Love	rural	284	28.7%	514.00	0.553	86.6%
40093	OK	Major	rural	251	28.6%	954.99	0.263	90.3%
40095	OK	Marshall	rural	499	28.8%	371.08	1.345	84.8%
40097	OK	Mayes	rural	1,056	28.6%	655.39	1.612	75.8%
40087	OK	McClain	urban	972	28.8%	570.70	1.702	77.5%
40089	OK	McCurtain	rural	856	28.6%	1,850.01	0.463	83.9%
40091	OK	McIntosh	rural	743	28.8%	618.50	1.201	82.2%
40099	OK	Murray	rural	400	28.4%	416.46	0.962	87.6%
40101	OK	Muskogee	rural	1,771	28.4%	810.45	2.185	77.0%
40103	OK	Noble	rural	302	28.5%	731.90	0.413	88.6%
40105	OK	Nowata	rural	317	28.4%	565.78	0.560	85.6%
40107	OK	Okfuskee	rural	289	28.5%	618.57	0.467	79.4%
40109	OK	Oklahoma	urban	14,348	28.6%	708.82	20.242	63.6%
40111	OK	Okmulgee	urban	937	28.4%	697.35	1.344	75.6%
40113	OK	Osage	urban	483	28.5%	2,246.36	0.215	75.0%
40115	OK	Ottawa	rural	907	28.6%	470.82	1.927	81.7%
40117	OK	Pawnee	urban	455	28.7%	567.95	0.801	78.5%
40119	OK	Payne	rural	1,721	28.8%	684.70	2.514	86.1%
40121	OK	Pittsburg	rural	1,271	28.5%	1,305.46	0.973	82.6%
40123	OK	Pontotoc	rural	978	28.7%	720.44	1.357	82.8%
40125	OK	Pottawatomie	rural	1,601	28.8%	787.67	2.033	70.0%
40127	OK	Pushmataha	rural	370	28.8%	1,395.84	0.265	82.6%
40129	OK	Roger Mills	rural	116	28.7%	1141.14	0.102	89.0%
40131	OK	Rogers	urban	1,500	28.7%	675.63	2.220	57.4%
40133	OK	Seminole	rural	609	28.5%	632.84	0.962	78.2%
40135	OK	Sequoyah	urban	1,025	28.7%	673.27	1.523	74.1%
40137	OK	Stephens	rural	1,189	28.7%	870.24	1.366	81.2%
40139	OK	Texas	rural	359	28.4%	2,041.26	0.176	82.3%
40141	OK	Tillman	rural	195	28.6%	871.13	0.224	86.8%
40143	OK	Tulsa	urban	11,866	28.6%	570.25	20.808	54.4%
40145	OK	Wagoner	urban	651	28.6%	561.56	1.159	56.2%
40147	OK	Washington	rural	1,707	28.6%	415.45	4.108	85.1%
40149	OK	Washita	rural	352	28.6%	1,003.17	0.351	87.4%
40151	OK	Woods	rural	263	28.5%	1,286.45	0.205	89.5%
40153	OK	Woodward	rural	500	28.6%	1,242.40	0.403	88.0%
41001	OR	Baker	rural	776	28.6%	3,068.36	0.253	87.3%
41003	OR	Benton	urban	1,157	28.5%	675.94	1.711	42.4%
41005	OR	Clackamas	urban	4,492	28.5%	1,870.32	2.402	27.3%
41007	OR	Clatsop	rural	1,105	28.7%	829.05	1.333	63.9%
41009	OR	Columbia	urban	721	28.5%	657.36	1.097	34.9%
41011	OR	Coos	rural	2,365	28.6%	1,596.17	1.482	78.8%
41013	OR	Crook	rural	818	28.7%	2,979.09	0.275	69.6%
41015	OR	Curry	rural	1,154	28.6%	1,627.46	0.709	79.9%
41017	OR	Deschutes	urban	5,013	28.7%	3,018.19	1.661	62.8%
41019	OR	Douglas	rural	3,228	28.6%	5,036.07	0.641	57.4%
41023	OR	Grant	rural	291	28.3%	4,528.54	0.064	71.4%
41025	OR	Harney	rural	285	28.4%	10,133.17	0.028	84.0%
41027	OR	Hood River	rural	486	28.4%	521.95	0.930	63.7%
41029	OR	Jackson	urban	5,945	28.6%	2,783.55	2.136	59.0%
41031	OR	Jefferson	rural	612	28.5%	1,780.78	0.344	63.4%
41033	OR	Josephine	urban	2,548	28.6%	1,639.67	1.554	53.3%
41035	OR	Klamath	rural	1,888	28.5%	5,941.05	0.318	69.3%
41037	OR	Lake	rural	331	28.4%	8,138.98	0.041	84.7%
41039	OR	Lane	urban	6,028	28.5%	4,553.12	1.324	39.0%
41041	OR	Lincoln	rural	1,783	28.8%	979.77	1.819	68.8%
41043	OR	Linn	urban	2,014	28.5%	2,290.13	0.879	37.6%
41045	OR	Malheur	rural	762	28.6%	9,887.53	0.077	81.1%
41047	OR	Marion	urban	3,411	28.5%	1,182.33	2.885	29.3%
41049	OR	Morrow	rural	256	28.8%	2,031.61	0.126	76.0%
41051	OR	Multnomah	urban	7,036	28.4%	431.30	16.314	28.8%
41053	OR	Polk	urban	1,015	28.6%	740.79	1.369	29.6%
41057	OR	Tillamook	rural	899	28.8%	1,102.58	0.816	65.2%

41059	OR	Umatilla	rural	1,703	28.6%	3,215.51	0.530	76.6%
41061	OR	Union	rural	876	28.6%	2,036.61	0.430	82.3%
41063	OR	Wallowa	rural	387	28.7%	3,146.19	0.123	88.8%
41065	OR	Wasco	rural	732	28.5%	2,381.52	0.308	65.6%
41067	OR	Washington	urban	4,328	28.6%	724.23	5.976	28.7%
41071	OR	Yamhill	urban	1,384	28.6%	715.86	1.933	36.4%
42001	PA	Adams	urban	2,153	28.6%	518.67	4.151	58.2%
42003	PA	Allegheny	urban	11,851	28.2%	730.07	16.233	23.5%
42005	PA	Armstrong	urban	827	28.2%	653.20	1.265	24.0%
42007	PA	Beaver	urban	1,783	28.1%	434.71	4.101	22.8%
42009	PA	Bedford	rural	834	28.5%	1,012.30	0.823	37.0%
42011	PA	Berks	urban	7,572	28.5%	856.51	8.841	54.3%
42013	PA	Blair	urban	1,950	28.3%	525.80	3.708	35.0%
42015	PA	Bradford	rural	1,532	28.5%	1,147.40	1.336	63.2%
42017	PA	Bucks	urban	11,940	28.6%	604.31	19.758	52.2%
42019	PA	Butler	urban	2,015	28.2%	788.60	2.555	26.3%
42021	PA	Cambria	urban	1,704	28.2%	688.35	2.476	25.9%
42023	PA	Cameron	rural	137	28.2%	396.23	0.345	60.9%
42025	PA	Carbon	urban	1,612	28.6%	381.46	4.225	69.9%
42027	PA	Centre	urban	1,650	28.4%	1,109.92	1.486	37.6%
42029	PA	Chester	urban	9,695	28.7%	750.51	12.918	61.2%
42031	PA	Clarion	rural	808	28.4%	600.83	1.344	54.8%
42033	PA	Clearfield	rural	1,513	28.4%	1,144.72	1.321	47.7%
42035	PA	Clinton	rural	632	28.6%	887.98	0.711	45.7%
42037	PA	Columbia	urban	1,300	28.7%	483.11	2.691	46.3%
42039	PA	Crawford	rural	1,888	28.5%	1,012.30	1.865	55.4%
42041	PA	Cumberland	urban	4,637	28.5%	545.46	8.500	48.3%
42043	PA	Dauphin	urban	3,449	28.3%	525.05	6.569	39.0%
42045	PA	Delaware	urban	10,119	28.6%	183.84	55.045	55.6%
42047	PA	Elk	rural	869	28.3%	827.36	1.050	69.1%
42049	PA	Erie	urban	4,084	28.4%	799.15	5.111	41.0%
42051	PA	Fayette	urban	1,836	28.3%	790.34	2.323	33.5%
42053	PA	Forest	rural	148	28.4%	427.19	0.348	53.3%
42055	PA	Franklin	urban	3,459	28.7%	772.22	4.479	64.2%
42057	PA	Fulton	rural	350	28.6%	437.55	0.799	65.8%
42059	PA	Greene	rural	541	28.2%	575.95	0.940	41.8%
42061	PA	Huntingdon	rural	871	28.4%	874.64	0.996	50.3%
42063	PA	Indiana	rural	1,028	28.3%	827.03	1.243	29.5%
42065	PA	Jefferson	rural	925	28.5%	652.43	1.417	51.1%
42067	PA	Juniata	rural	354	28.4%	391.35	0.905	40.1%
42069	PA	Lackawanna	urban	4,640	28.5%	459.08	10.108	58.3%
42071	PA	Lancaster	urban	9,833	28.5%	943.81	10.418	52.6%
42073	PA	Lawrence	rural	1,234	28.3%	358.18	3.445	29.0%
42075	PA	Lebanon	urban	2,728	28.6%	361.83	7.540	50.7%
42077	PA	Lehigh	urban	6,914	28.6%	345.17	20.031	57.3%
42079	PA	Luzerne	urban	7,256	28.6%	890.33	8.150	61.2%
42081	PA	Lycoming	urban	2,392	28.5%	1,228.59	1.947	54.6%
42083	PA	McKean	rural	1,019	28.4%	979.20	1.041	68.0%
42085	PA	Mercer	urban	1,967	28.5%	672.57	2.925	40.5%
42087	PA	Mifflin	rural	795	28.6%	411.03	1.934	41.3%
42089	PA	Monroe	urban	3,232	28.6%	608.29	5.314	68.1%
42091	PA	Montgomery	urban	17,094	28.6%	483.04	35.388	56.6%
42093	PA	Montour	urban	301	28.5%	130.24	2.309	36.4%
42095	PA	Northampton	urban	6,394	28.6%	369.67	17.297	61.3%
42097	PA	Northumberland	rural	1,881	28.6%	458.37	4.103	50.8%
42099	PA	Perry	urban	684	28.5%	551.45	1.240	41.0%
42101	PA	Philadelphia	urban	14,965	28.4%	134.10	111.593	39.5%
42103	PA	Pike	urban	1,154	28.8%	544.96	2.118	78.4%
42105	PA	Potter	rural	482	28.4%	1,081.32	0.446	60.9%
42107	PA	Schuylkill	rural	3,224	28.5%	778.63	4.141	58.2%
42109	PA	Snyder	rural	649	28.6%	328.71	1.974	44.3%
42111	PA	Somerset	rural	1,049	28.2%	1,074.37	0.977	29.4%
42113	PA	Sullivan	rural	193	28.7%	449.94	0.430	61.9%
42115	PA	Susquehanna	rural	1,094	28.6%	823.43	1.329	69.6%
42117	PA	Tioga	rural	1,132	28.6%	1,133.79	0.999	65.0%
42119	PA	Union	rural	780	28.5%	315.98	2.467	50.8%
42121	PA	Venango	rural	1,075	28.6%	674.28	1.595	50.3%
42123	PA	Warren	rural	1,095	28.6%	884.13	1.238	68.9%
42125	PA	Washington	urban	2,431	28.2%	856.99	2.836	25.6%
42127	PA	Wayne	rural	2,093	28.7%	725.60	2.885	73.6%
42129	PA	Westmoreland	urban	3,974	28.2%	1,027.55	3.867	22.6%
42131	PA	Wyoming	urban	707	28.6%	397.32	1.778	58.4%
42133	PA	York	urban	8,075	28.5%	904.18	8.931	54.0%
72005	PR	Aguadilla		132	28.5%	36.53	3.623	6.3%
72013	PR	Arecibo		188	29.5%	125.95	1.494	5.1%
72021	PR	Bayam+¦n		482	28.6%	44.32	10.882	6.8%
72023	PR	Cabo Rojo		107	29.3%	70.37	1.526	6.1%
72025	PR	Caguas		339	29.2%	58.60	5.782	7.0%
72031	PR	Carolina		339	28.6%	45.32	7.474	6.8%
72033	PR	Cata+¦o		153	29.0%	4.84	31.659	6.6%
72035	PR	Cayey		109	28.3%	51.93	2.095	7.1%
72061	PR	Guaynabo		333	29.2%	27.58	12.085	12.9%

72069	PR	Humacao		132	28.3%	44.75	2.949	7.3%
72087	PR	Lo+¡za		104	29.5%	19.37	5.385	5.6%
72097	PR	Mayag++ez		193	29.0%	77.65	2.484	5.9%
72113	PR	Ponce		411	28.9%	114.76	3.580	7.1%
72127	PR	San Juan		1,645	28.8%	47.85	34.380	11.7%
72139	PR	Trujillo Alto		128	29.9%	20.76	6.176	7.7%
44001	RI	Bristol	urban	874	28.7%	24.16	36.160	42.3%
44003	RI	Kent	urban	2,640	28.7%	168.53	15.665	40.5%
44005	RI	Newport	urban	2,055	28.6%	102.39	20.067	58.4%
44007	RI	Providence	urban	7,687	28.6%	409.50	18.771	40.2%
44009	RI	Washington	urban	2,775	28.6%	329.23	8.427	53.2%
45001	SC	Abbeville	rural	491	28.1%	490.48	1.001	68.0%
45003	SC	Aiken	urban	4,195	28.5%	1,071.03	3.916	71.7%
45005	SC	Allendale	rural	152	28.1%	408.09	0.371	60.6%
45007	SC	Anderson	urban	3,894	28.5%	715.43	5.444	63.7%
45009	SC	Bamberg	rural	273	28.4%	393.37	0.693	66.8%
45011	SC	Barnwell	rural	441	28.5%	548.39	0.805	69.5%
45013	SC	Beaufort	urban	7,151	28.8%	576.28	12.409	78.7%
45015	SC	Berkeley	urban	2,574	28.5%	1,098.86	2.343	71.2%
45017	SC	Calhoun	urban	236	28.6%	381.15	0.619	68.4%
45019	SC	Charleston	urban	9,318	28.5%	916.09	10.171	73.8%
45021	SC	Cherokee	rural	944	28.3%	392.66	2.405	66.5%
45023	SC	Chester	urban	730	28.4%	580.66	1.257	72.4%
45025	SC	Chesterfield	rural	927	28.4%	799.08	1.160	79.2%
45027	SC	Clarendon	rural	804	28.5%	606.94	1.325	71.3%
45029	SC	Colleton	rural	762	28.4%	1,056.49	0.721	67.8%
45031	SC	Darlington	urban	1,470	28.4%	561.15	2.619	79.6%
45033	SC	Dillon	rural	592	28.4%	404.87	1.462	77.8%
45035	SC	Dorchester	urban	2,457	28.6%	573.23	4.285	71.1%
45037	SC	Edgefield	urban	364	28.1%	500.41	0.728	67.8%
45039	SC	Fairfield	urban	459	28.2%	686.28	0.669	65.9%
45041	SC	Florence	urban	3,352	28.4%	799.96	4.191	81.8%
45043	SC	Georgetown	rural	2,454	28.6%	813.55	3.016	79.4%
45045	SC	Greenville	urban	9,209	28.6%	785.12	11.730	62.3%
45047	SC	Greenwood	rural	1,842	28.5%	454.73	4.050	72.8%
45049	SC	Hampton	rural	463	27.9%	559.90	0.827	73.4%
45051	SC	Horry	urban	10,098	28.7%	1,133.90	8.905	78.2%
45053	SC	Jasper	urban	376	28.2%	655.32	0.574	64.9%
45055	SC	Kershaw	urban	1,581	28.5%	726.56	2.176	75.4%
45057	SC	Lancaster	urban	1,461	28.4%	549.16	2.661	79.6%
45059	SC	Laurens	urban	1,203	28.2%	713.80	1.686	65.2%
45061	SC	Lee	rural	325	27.9%	410.18	0.792	76.2%
45063	SC	Lexington	urban	6,004	28.6%	698.91	8.591	72.5%
45067	SC	Marion	rural	794	28.4%	489.23	1.623	79.8%
45069	SC	Marlboro	rural	548	28.1%	479.67	1.142	74.4%
45065	SC	McCormick	rural	403	28.6%	359.13	1.121	65.9%
45071	SC	Newberry	rural	974	28.5%	630.04	1.545	72.3%
45073	SC	Oconee	rural	2,270	28.6%	626.33	3.624	71.9%
45075	SC	Orangeburg	rural	1,720	28.3%	1,106.10	1.555	63.3%
45077	SC	Pickens	urban	2,626	28.6%	496.41	5.289	61.8%
45079	SC	Richland	urban	6,590	28.5%	757.07	8.705	72.2%
45081	SC	Saluda	urban	315	28.4%	452.78	0.696	68.2%
45083	SC	Spartanburg	urban	5,994	28.5%	807.93	7.419	59.1%
45085	SC	Sumter	urban	2,191	28.2%	665.07	3.294	78.2%
45087	SC	Union	urban	599	28.2%	514.17	1.165	65.5%
45089	SC	Williamsburg	rural	639	28.1%	934.16	0.684	71.9%
45091	SC	York	urban	5,962	28.7%	680.59	8.760	70.6%
46005	SD	Beadle	rural	487	28.5%	1,258.71	0.387	72.4%
46009	SD	Bon Homme	rural	239	28.6%	563.7	0.423	78.2%
46011	SD	Brookings	rural	552	28.5%	792.21	0.697	72.0%
46013	SD	Brown	rural	1,028	28.4%	1,712.98	0.600	82.3%
46015	SD	Brule	rural	156	28.4%	817.24	0.191	77.2%
46019	SD	Butte	rural	274	28.3%	2,249.90	0.122	65.6%
46023	SD	Charles Mix	rural	262	28.7%	1097.49	0.239	83.1%
46027	SD	Clay	rural	254	28.3%	412.19	0.617	75.0%
46029	SD	Codington	rural	509	28.4%	688.50	0.740	53.8%
46033	SD	Custer	urban	356	28.8%	1,557.00	0.229	74.0%
46035	SD	Davison	rural	571	28.6%	435.56	1.312	79.7%
46037	SD	Day	rural	197	28.5%	1,027.87	0.192	70.4%
46039	SD	Deuel	rural	113	28.5%	622.69	0.181	62.2%
46043	SD	Douglas	rural	124	28.4%	431.80	0.286	78.5%
46045	SD	Edmunds	rural	148	28.8%	1,125.96	0.131	85.1%
46047	SD	Fall River	rural	340	28.4%	1739.92	0.195	72.9%
46051	SD	Grant	rural	188	28.4%	681.46	0.276	55.9%
46053	SD	Gregory	rural	183	28.4%	1,014.96	0.181	78.7%
46057	SD	Hamlin	rural	126	28.4%	507.23	0.249	61.8%
46059	SD	Hand	rural	108	28.2%	1,436.61	0.075	83.7%
46061	SD	Hanson	rural	210	28.7%	434.51	0.484	80.5%
46065	SD	Hughes	rural	470	28.3%	741.56	0.634	79.6%
46067	SD	Hutchinson	rural	265	28.2%	812.90	0.326	73.4%
46077	SD	Kingsbury	rural	212	28.2%	832.24	0.254	76.5%
46079	SD	Lake	rural	537	28.4%	563.28	0.954	77.1%

46081	SD	Lawrence	rural	725	28.6%	800.04	0.906	69.9%
46083	SD	Lincoln	urban	307	28.5%	577.28	0.533	61.4%
46091	SD	Marshall	rural	164	28.5%	838.07	0.196	84.1%
46087	SD	McCook	urban	154	28.8%	574.20	0.268	76.5%
46093	SD	Meade	urban	585	28.3%	3,470.98	0.168	68.5%
46099	SD	Minnehaha	urban	4,433	28.6%	807.15	5.492	67.0%
46101	SD	Moody	rural	146	28.2%	519.39	0.282	74.7%
46103	SD	Pennington	urban	3,025	28.5%	2,776.55	1.090	71.3%
46105	SD	Perkins	rural	119	28.0%	2,870.48	0.041	79.6%
46107	SD	Potter	rural	131	28.4%	861.14	0.152	89.4%
46109	SD	Roberts	rural	236	28.4%	1,101.04	0.214	66.9%
46115	SD	Spink	rural	231	28.1%	1,503.93	0.153	84.1%
46123	SD	Tripp	rural	213	28.3%	1,612.45	0.132	85.7%
46125	SD	Turner	urban	245	28.8%	617.06	0.396	66.6%
46127	SD	Union	urban	406	28.4%	460.54	0.882	70.0%
46129	SD	Walworth	rural	196	28.7%	708.63	0.276	87.4%
46135	SD	Yankton	rural	602	28.4%	521.16	1.156	72.3%
47001	TN	Anderson	urban	1,538	28.4%	337.16	4.561	54.1%
47003	TN	Bedford	rural	849	28.5%	473.64	1.792	62.2%
47005	TN	Benton	rural	496	28.8%	394.14	1.258	73.5%
47007	TN	Bledsoe	rural	229	28.3%	406.42	0.563	66.2%
47009	TN	Blount	urban	2,349	28.5%	558.71	4.204	49.4%
47011	TN	Bradley	urban	1,805	28.6%	328.76	5.491	53.8%
47013	TN	Campbell	urban	623	28.5%	480.19	1.297	43.8%
47015	TN	Cannon	urban	304	28.4%	265.64	1.143	60.7%
47017	TN	Carroll	rural	895	28.6%	599.25	1.493	76.6%
47019	TN	Carter	urban	760	28.5%	341.20	2.227	41.1%
47021	TN	Cheatham	urban	528	28.5%	302.44	1.746	44.4%
47023	TN	Chester	urban	306	28.4%	285.74	1.071	73.4%
47025	TN	Claiborne	rural	537	28.6%	434.58	1.235	45.0%
47027	TN	Clay	rural	179	28.5%	236.54	0.756	81.0%
47029	TN	Cocke	rural	671	28.4%	434.57	1.543	47.1%
47031	TN	Coffee	rural	1,427	28.6%	428.96	3.328	69.3%
47033	TN	Crockett	urban	381	28.5%	265.53	1.434	80.2%
47035	TN	Cumberland	rural	2,345	28.8%	681.03	3.443	73.2%
47037	TN	Davidson	urban	7,733	28.4%	504.03	15.342	45.5%
47041	TN	DeKalb	rural	312	28.6%	304.35	1.025	46.0%
47039	TN	Decatur	rural	270	28.7%	333.84	0.808	69.8%
47043	TN	Dickson	urban	834	28.6%	489.90	1.702	51.9%
47045	TN	Dyer	rural	913	28.6%	512.33	1.782	80.8%
47047	TN	Fayette	urban	672	28.8%	704.79	0.953	65.8%
47049	TN	Fentress	rural	520	28.8%	498.61	1.042	78.2%
47051	TN	Franklin	rural	845	28.7%	554.54	1.524	63.2%
47053	TN	Gibson	rural	1,343	28.5%	602.74	2.229	78.1%
47055	TN	Giles	rural	802	28.6%	610.93	1.312	75.4%
47057	TN	Grainger	urban	378	28.2%	280.60	1.347	41.6%
47059	TN	Greene	rural	1,442	28.6%	622.16	2.318	50.3%
47061	TN	Grundy	rural	328	28.6%	360.53	0.908	60.7%
47063	TN	Hamblen	urban	1,064	28.6%	161.18	6.602	50.2%
47065	TN	Hamilton	urban	6,628	28.5%	542.43	12.218	55.6%
47069	TN	Hardeman	rural	521	28.6%	667.77	0.780	73.0%
47071	TN	Hardin	rural	733	28.5%	577.32	1.270	81.3%
47073	TN	Hawkins	urban	743	28.5%	486.98	1.526	33.2%
47075	TN	Haywood	rural	343	28.3%	533.11	0.644	72.0%
47077	TN	Henderson	rural	664	28.5%	520.07	1.277	70.0%
47079	TN	Henry	rural	1,097	28.7%	562.10	1.951	77.9%
47081	TN	Hickman	urban	446	28.5%	612.50	0.729	54.4%
47083	TN	Houston	rural	215	28.2%	200.29	1.076	72.6%
47085	TN	Humphreys	rural	496	28.5%	530.98	0.935	73.2%
47087	TN	Jackson	rural	250	28.7%	308.32	0.812	74.3%
47089	TN	Jefferson	urban	1,172	28.6%	274.08	4.274	48.1%
47091	TN	Johnson	rural	415	28.7%	298.47	1.391	50.8%
47093	TN	Knox	urban	6,934	28.5%	508.22	13.644	47.1%
47095	TN	Lake	rural	146	28.6%	165.78	0.882	85.3%
47097	TN	Lauderdale	rural	536	28.4%	471.99	1.136	74.3%
47099	TN	Lawrence	rural	1,289	28.6%	617.13	2.089	78.5%
47101	TN	Lewis	rural	266	28.5%	282.09	0.943	69.0%
47103	TN	Lincoln	rural	843	28.7%	570.34	1.478	65.6%
47105	TN	Loudon	urban	1,539	28.6%	229.22	6.715	52.3%
47111	TN	Macon	urban	405	28.5%	307.14	1.320	64.0%
47113	TN	Madison	urban	2,086	28.5%	557.12	3.744	73.5%
47115	TN	Marion	urban	511	28.5%	498.16	1.025	58.0%
47117	TN	Marshall	rural	577	28.4%	375.46	1.537	59.9%
47119	TN	Maury	urban	1,971	28.5%	613.14	3.215	62.6%
47107	TN	McMinn	rural	1,202	28.6%	430.12	2.795	63.6%
47109	TN	McNairy	rural	893	28.7%	562.86	1.587	79.8%
47121	TN	Meigs	rural	289	28.5%	195.12	1.481	59.6%
47123	TN	Monroe	rural	946	28.6%	635.56	1.489	52.0%
47125	TN	Montgomery	urban	2,275	28.5%	539.18	4.218	70.4%
47129	TN	Morgan	urban	229	28.3%	522.18	0.438	43.3%
47131	TN	Obion	rural	989	28.6%	544.73	1.816	83.1%
47133	TN	Overton	rural	593	28.7%	433.48	1.369	78.4%

47135	TN	Perry	rural	198	28.8%	414.73	0.477	61.5%
47137	TN	Pickett	rural	140	28.4%	162.98	0.856	78.9%
47139	TN	Polk	urban	445	28.5%	434.68	1.025	66.7%
47141	TN	Putnam	rural	2,058	28.7%	401.10	5.130	71.3%
47143	TN	Rhea	rural	771	28.5%	315.38	2.446	64.0%
47145	TN	Roane	urban	1,073	28.3%	360.71	2.974	49.0%
47147	TN	Robertson	urban	985	28.6%	476.29	2.068	47.2%
47149	TN	Rutherford	urban	3,047	28.6%	619.36	4.920	50.0%
47151	TN	Scott	rural	319	28.6%	532.30	0.600	57.3%
47153	TN	Sequatchie	urban	272	28.6%	265.86	1.023	61.3%
47155	TN	Sevier	rural	1,796	28.5%	592.50	3.032	47.0%
47157	TN	Shelby	urban	14,360	28.4%	763.17	18.816	63.1%
47159	TN	Smith	urban	363	28.6%	314.29	1.153	61.6%
47161	TN	Stewart	rural	362	28.5%	459.33	0.788	74.0%
47163	TN	Sullivan	urban	2,372	28.5%	413.36	5.738	33.4%
47165	TN	Sumner	urban	2,358	28.7%	529.45	4.454	44.0%
47167	TN	Tipton	urban	1,060	28.3%	458.37	2.313	69.6%
47169	TN	Trousdale	urban	114	28.3%	114.19	1.000	52.4%
47171	TN	Unicoi	urban	374	28.5%	186.17	2.008	47.2%
47173	TN	Union	urban	190	28.4%	223.55	0.849	34.7%
47175	TN	Van Buren	rural	115	28.5%	273.42	0.420	74.0%
47177	TN	Warren	rural	1,012	28.7%	432.68	2.339	68.5%
47179	TN	Washington	urban	2,203	28.5%	326.46	6.747	43.9%
47181	TN	Wayne	rural	359	28.3%	734.10	0.489	74.1%
47183	TN	Weakley	rural	803	28.4%	580.36	1.384	80.8%
47185	TN	White	rural	744	28.6%	376.67	1.976	71.3%
47187	TN	Williamson	urban	3,074	28.5%	582.60	5.276	51.4%
47189	TN	Wilson	urban	1,935	28.6%	570.83	3.390	51.0%
48001	TX	Anderson	rural	1,098	28.5%	1,062.60	1.033	67.4%
48003	TX	Andrews	rural	223	28.1%	1,500.71	0.149	71.7%
48005	TX	Angelina	rural	1,711	28.5%	797.78	2.145	66.8%
48007	TX	Aransas	urban	652	28.6%	252.07	2.585	55.1%
48009	TX	Archer	urban	156	28.8%	903.11	0.173	77.7%
48013	TX	Atascosa	urban	527	28.6%	1,219.54	0.432	49.4%
48015	TX	Austin	urban	776	28.6%	646.51	1.200	69.3%
48017	TX	Bailey	rural	143	28.6%	826.80	0.173	79.3%
48019	TX	Bandera	urban	667	28.5%	790.96	0.843	66.4%
48021	TX	Bastrop	urban	1,445	28.4%	888.15	1.627	64.1%
48023	TX	Baylor	rural	127	28.5%	867.48	0.146	78.5%
48025	TX	Bee	rural	347	28.6%	880.24	0.395	56.8%
48027	TX	Bell	urban	3,405	28.5%	1,051.02	3.240	57.6%
48029	TX	Bexar	urban	19,982	28.6%	1,239.82	16.117	46.9%
48031	TX	Blanco	rural	410	28.5%	709.25	0.579	72.1%
48035	TX	Bosque	rural	513	28.5%	982.98	0.522	60.5%
48037	TX	Bowie	urban	1,925	28.6%	885.01	2.175	69.6%
48039	TX	Brazoria	urban	4,177	28.7%	1,357.70	3.076	54.5%
48041	TX	Brazos	urban	2,458	28.6%	585.45	4.199	69.0%
48043	TX	Brewster	rural	249	28.4%	6,183.73	0.040	64.9%
48049	TX	Brown	rural	1,061	28.6%	944.43	1.124	73.2%
48051	TX	Burleson	urban	438	28.3%	659.03	0.664	67.8%
48053	TX	Burnet	rural	1,746	28.6%	994.26	1.756	69.3%
48055	TX	Caldwell	urban	642	28.5%	545.26	1.177	64.9%
48057	TX	Calhoun	rural	423	28.4%	506.84	0.835	76.7%
48059	TX	Callahan	urban	369	28.4%	899.37	0.410	70.5%
48061	TX	Cameron	urban	3,870	28.5%	890.92	4.344	47.6%
48063	TX	Camp	rural	327	28.5%	195.83	1.672	67.0%
48065	TX	Carson	urban	146	28.4%	920.22	0.158	72.9%
48067	TX	Cass	rural	851	28.5%	936.96	0.908	66.7%
48069	TX	Castro	rural	155	28.2%	894.43	0.173	80.4%
48071	TX	Chambers	urban	305	28.2%	597.14	0.511	60.8%
48073	TX	Cherokee	rural	869	28.6%	1,052.91	0.825	65.1%
48075	TX	Childress	rural	166	28.7%	696.40	0.239	72.3%
48077	TX	Clay	urban	219	28.4%	1,088.72	0.201	77.1%
48081	TX	Coke	rural	101	28.6%	911.47	0.111	72.2%
48083	TX	Coleman	rural	330	28.3%	1,261.95	0.261	80.5%
48085	TX	Collin	urban	11,063	28.8%	841.22	13.151	61.6%
48089	TX	Colorado	rural	629	28.2%	960.27	0.655	81.6%
48091	TX	Comal	urban	3,370	28.6%	559.48	6.023	63.1%
48093	TX	Comanche	rural	437	28.7%	937.75	0.466	74.4%
48097	TX	Cooke	rural	894	28.6%	874.76	1.022	71.1%
48099	TX	Coryell	urban	815	28.8%	1,052.07	0.775	66.3%
48107	TX	Crosby	urban	121	28.4%	900.20	0.134	64.6%
48111	TX	Dallam	rural	225	28.8%	1,503.26	0.150	76.1%
48113	TX	Dallas	urban	30,820	28.6%	871.28	35.374	56.1%
48115	TX	Dawson	rural	271	28.7%	900.31	0.301	74.7%
48123	TX	DeWitt	rural	444	28.6%	908.98	0.488	79.8%
48117	TX	Deaf Smith	rural	343	28.4%	1496.87	0.229	76.9%
48119	TX	Delta	rural	163	28.6%	256.83	0.635	77.9%
48121	TX	Denton	urban	6,833	28.7%	878.43	7.779	58.1%
48127	TX	Dimmit	rural	129	28.4%	1,328.88	0.097	57.0%
48129	TX	Donley	rural	117	28.6%	926.89	0.126	75.3%
48131	TX	Duval	rural	153	28.5%	1,793.48	0.086	54.0%

48133	TX	Eastland	rural	574	28.9%	926.49	0.619	71.0%
48135	TX	Ector	urban	2,061	28.4%	897.69	2.296	69.9%
48141	TX	El Paso	urban	7,479	28.5%	1012.69	7.385	41.0%
48139	TX	Ellis	urban	2,684	28.5%	935.49	2.869	62.4%
48143	TX	Erath	rural	757	28.5%	1,083.07	0.699	69.6%
48145	TX	Falls	urban	235	27.9%	765.48	0.307	56.8%
48147	TX	Fannin	rural	836	28.7%	890.84	0.939	72.5%
48149	TX	Fayette	rural	933	28.4%	950.01	0.983	78.0%
48151	TX	Fisher	rural	113	28.8%	898.94	0.126	76.2%
48153	TX	Floyd	rural	138	28.8%	992.14	0.139	69.9%
48157	TX	Fort Bend	urban	5,160	28.7%	861.48	5.989	48.7%
48159	TX	Franklin	rural	238	28.9%	284.39	0.835	72.1%
48161	TX	Freestone	rural	426	28.6%	877.74	0.485	71.3%
48163	TX	Frio	rural	182	28.7%	1,133.50	0.161	58.6%
48165	TX	Gaines	rural	219	28.4%	1,502.38	0.146	78.4%
48167	TX	Galveston	urban	5,228	28.4%	378.36	13.818	60.4%
48171	TX	Gillespie	rural	1,210	28.6%	1,058.21	1.143	77.4%
48175	TX	Goliad	urban	161	28.2%	852.01	0.188	73.1%
48177	TX	Gonzales	rural	421	28.4%	1,066.69	0.394	72.7%
48179	TX	Gray	rural	583	28.8%	925.97	0.629	76.4%
48181	TX	Grayson	urban	3,077	28.6%	932.80	3.299	71.9%
48183	TX	Gregg	urban	2,909	28.5%	273.30	10.643	68.5%
48185	TX	Grimes	rural	552	28.2%	787.46	0.700	66.3%
48187	TX	Guadalupe	urban	2,172	28.5%	711.30	3.054	63.0%
48189	TX	Hale	rural	665	28.5%	1,004.68	0.662	71.9%
48191	TX	Hall	rural	106	29.0%	883.49	0.120	77.7%
48193	TX	Hamilton	rural	229	28.8%	835.91	0.274	64.1%
48195	TX	Hansford	rural	145	28.4%	919.81	0.157	81.7%
48197	TX	Hardeman	rural	115	28.3%	695.11	0.165	75.9%
48199	TX	Hardin	urban	918	28.7%	890.57	1.031	53.2%
48201	TX	Harris	urban	41,061	28.6%	1,703.48	24.104	47.1%
48203	TX	Harrison	rural	1,067	28.1%	899.95	1.186	67.9%
48207	TX	Haskell	rural	183	28.7%	903.13	0.202	75.7%
48209	TX	Hays	urban	2,354	28.6%	677.98	3.473	61.5%
48213	TX	Henderson	rural	1,624	28.6%	873.75	1.859	66.4%
48215	TX	Hidalgo	urban	6,085	28.6%	1,570.87	3.873	51.7%
48217	TX	Hill	rural	938	28.5%	958.86	0.978	68.3%
48219	TX	Hockley	rural	408	28.4%	908.39	0.449	68.5%
48221	TX	Hood	urban	1,906	28.6%	420.64	4.531	67.5%
48223	TX	Hopkins	rural	936	28.6%	767.17	1.220	79.1%
48225	TX	Houston	rural	627	28.2%	1,230.91	0.509	73.1%
48227	TX	Howard	rural	614	28.4%	900.79	0.682	72.9%
48231	TX	Hunt	urban	1,819	28.7%	840.32	2.164	74.6%
48233	TX	Hutchinson	rural	526	28.5%	887.42	0.593	77.9%
48237	TX	Jack	rural	192	28.5%	910.66	0.211	76.9%
48239	TX	Jackson	rural	253	28.5%	829.43	0.305	70.5%
48241	TX	Jasper	rural	702	28.5%	938.85	0.748	63.5%
48245	TX	Jefferson	urban	3,415	28.4%	876.30	3.897	53.7%
48249	TX	Jim Wells	rural	493	28.5%	864.97	0.569	46.4%
48251	TX	Johnson	urban	2,394	28.7%	724.69	3.304	47.2%
48253	TX	Jones	urban	338	28.4%	928.55	0.364	73.2%
48255	TX	Karnes	rural	310	28.1%	747.56	0.414	73.6%
48257	TX	Kaufman	urban	2,186	28.5%	780.70	2.799	61.9%
48259	TX	Kendall	urban	1,484	28.8%	662.45	2.240	66.6%
48265	TX	Kerr	rural	2,265	28.8%	1,103.32	2.053	78.0%
48267	TX	Kimble	rural	162	28.9%	1,250.99	0.130	77.0%
48273	TX	Kleberg	rural	319	28.7%	881.31	0.362	48.1%
48277	TX	Lamar	rural	1,480	28.5%	907.19	1.632	83.8%
48279	TX	Lamb	rural	304	28.4%	1,016.18	0.299	71.7%
48281	TX	Lampasas	urban	505	28.7%	712.84	0.708	65.4%
48285	TX	Lavaca	rural	741	28.4%	969.71	0.764	79.0%
48287	TX	Lee	rural	353	28.3%	629.02	0.562	67.2%
48289	TX	Leon	rural	601	28.5%	1,073.15	0.560	68.6%
48291	TX	Liberty	urban	1,120	28.3%	1,158.42	0.967	50.6%
48293	TX	Limestone	rural	448	28.4%	905.29	0.495	64.5%
48297	TX	Live Oak	rural	167	28.7%	1039.7	0.161	63.4%
48299	TX	Llano	rural	786	28.8%	934.03	0.842	70.3%
48303	TX	Lubbock	urban	4,319	28.6%	895.60	4.822	60.8%
48305	TX	Lynn	urban	134	28.9%	891.87	0.150	72.8%
48313	TX	Madison	rural	252	28.8%	466.07	0.541	67.8%
48315	TX	Marion	rural	254	28.4%	380.88	0.667	69.4%
48319	TX	Mason	rural	169	28.6%	928.80	0.182	75.4%
48321	TX	Matagorda	rural	632	28.5%	1,100.28	0.575	65.4%
48323	TX	Maverick	rural	697	27.9%	1,279.26	0.545	55.5%
48307	TX	McCulloch	rural	264	28.6%	1,065.60	0.247	79.5%
48309	TX	McLennan	urban	4,020	28.5%	1,037.10	3.876	59.2%
48325	TX	Medina	urban	691	28.6%	1,325.36	0.521	55.4%
48329	TX	Midland	urban	2,109	28.3%	900.30	2.342	70.7%
48331	TX	Milam	rural	489	28.1%	1,016.93	0.481	56.6%
48333	TX	Mills	rural	139	28.1%	748.26	0.185	63.8%
48335	TX	Mitchell	rural	194	28.9%	911.09	0.213	75.8%
48337	TX	Montague	rural	690	28.6%	930.91	0.741	80.4%

48339	TX	Montgomery	urban	7,015	28.6%	1,041.73	6.734	52.4%
48341	TX	Moore	rural	316	28.4%	899.69	0.351	76.1%
48343	TX	Morris	rural	330	28.3%	251.98	1.309	61.9%
48347	TX	Nacogdoches	rural	1,214	28.5%	946.54	1.283	71.5%
48349	TX	Navarro	rural	1,023	28.5%	1,009.63	1.013	71.0%
48351	TX	Newton	urban	238	28.4%	933.68	0.255	65.5%
48353	TX	Nolan	rural	377	28.3%	912.00	0.413	74.2%
48355	TX	Nueces	urban	3,440	28.7%	838.48	4.103	39.5%
48357	TX	Ochiltree	rural	197	28.8%	917.63	0.215	84.3%
48361	TX	Orange	urban	1,407	28.4%	333.67	4.218	55.7%
48363	TX	Palo Pinto	rural	715	28.6%	951.79	0.752	78.1%
48365	TX	Panola	rural	516	28.5%	801.75	0.644	71.5%
48367	TX	Parker	urban	2,306	28.6%	903.48	2.552	66.7%
48369	TX	Parmer	rural	207	28.5%	880.78	0.235	84.8%
48371	TX	Pecos	rural	228	28.1%	4,763.85	0.048	68.2%
48373	TX	Polk	rural	2,038	28.6%	1,057.09	1.928	66.8%
48375	TX	Potter	urban	4,118	28.6%	908.37	4.534	68.9%
48377	TX	Presidio	rural	195	27.1%	3,855.24	0.051	71.3%
48379	TX	Rains	rural	345	28.6%	229.45	1.503	73.9%
48381	TX	Randall	urban	653	28.5%	911.54	0.716	73.9%
48385	TX	Real	rural	145	28.8%	699.19	0.207	75.6%
48387	TX	Red River	rural	401	28.5%	1036.58	0.386	79.8%
48389	TX	Reeves	rural	208	27.9%	2,635.37	0.079	73.1%
48391	TX	Refugio	rural	165	28.3%	770.44	0.215	68.0%
48395	TX	Robertson	urban	337	28.4%	855.68	0.393	68.4%
48397	TX	Rockwall	urban	1,494	28.7%	127.04	11.759	64.9%
48399	TX	Runnels	rural	367	28.7%	1,050.94	0.350	83.6%
48401	TX	Rusk	urban	860	28.4%	924.03	0.930	67.8%
48403	TX	Sabine	rural	489	28.6%	491.39	0.996	75.5%
48405	TX	San Augustine	rural	233	28.2%	530.66	0.440	72.6%
48407	TX	San Jacinto	rural	354	28.6%	569.24	0.621	52.7%
48409	TX	San Patricio	urban	859	28.8%	693.45	1.239	41.0%
48411	TX	San Saba	rural	176	28.4%	1135.3	0.155	68.8%
48415	TX	Scurry	rural	354	28.5%	905.44	0.391	72.4%
48419	TX	Shelby	rural	580	28.3%	795.58	0.729	72.9%
48423	TX	Smith	urban	4,971	28.5%	921.45	5.395	70.2%
48425	TX	Somervell	urban	175	28.1%	186.46	0.941	65.0%
48427	TX	Starr	rural	696	27.8%	1,223.18	0.569	67.6%
48429	TX	Stephens	rural	223	28.4%	896.72	0.248	73.0%
48435	TX	Sutton	rural	102	28.8%	1,453.93	0.070	86.5%
48437	TX	Swisher	rural	232	28.3%	890.16	0.261	82.4%
48439	TX	Tarrant	urban	19,537	28.6%	863.61	22.623	45.9%
48441	TX	Taylor	urban	2,867	28.6%	915.55	3.131	71.3%
48445	TX	Terry	rural	209	28.6%	888.84	0.235	64.3%
48449	TX	Titus	rural	571	28.5%	406.05	1.407	69.6%
48451	TX	Tom Green	urban	2,315	28.5%	1521.97	1.521	71.2%
48453	TX	Travis	urban	12,967	28.5%	990.20	13.096	61.8%
48455	TX	Trinity	rural	419	28.5%	693.61	0.604	65.4%
48457	TX	Tyler	rural	500	28.6%	924.50	0.541	68.2%
48459	TX	Upshur	urban	788	28.4%	582.95	1.351	65.0%
48463	TX	Uvalde	rural	503	28.5%	1,551.95	0.324	63.8%
48465	TX	Val Verde	rural	733	28.0%	3144.75	0.233	63.8%
48467	TX	Van Zandt	rural	1,366	28.6%	842.56	1.621	69.1%
48469	TX	Victoria	urban	1,589	28.6%	882.14	1.802	67.2%
48471	TX	Walker	rural	915	28.6%	784.17	1.167	58.8%
48473	TX	Waller	urban	478	28.2%	513.43	0.930	56.7%
48475	TX	Ward	rural	162	28.5%	835.60	0.194	68.4%
48477	TX	Washington	rural	1,079	28.4%	603.95	1.786	74.8%
48479	TX	Webb	urban	2,453	28.5%	3,361.48	0.730	62.0%
48481	TX	Wharton	rural	920	28.3%	1,086.15	0.847	74.4%
48483	TX	Wheeler	rural	168	28.6%	914.52	0.184	80.4%
48485	TX	Wichita	urban	2,746	28.5%	627.78	4.374	77.7%
48487	TX	Wilbarger	rural	270	28.3%	970.84	0.278	67.1%
48489	TX	Willacy	rural	200	28.8%	590.55	0.338	50.0%
48491	TX	Williamson	urban	7,463	28.8%	1,118.30	6.673	61.1%
48493	TX	Wilson	urban	623	28.4%	803.73	0.775	55.1%
48495	TX	Winkler	rural	119	28.6%	841.11	0.142	74.6%
48497	TX	Wise	urban	1,096	28.6%	904.42	1.212	65.1%
48499	TX	Wood	rural	1,667	28.8%	645.23	2.584	69.8%
48501	TX	Yoakum	rural	152	28.5%	799.71	0.190	77.1%
48503	TX	Young	rural	605	28.5%	914.47	0.661	87.3%
48505	TX	Zapata	rural	174	28.3%	998.41	0.174	74.2%
48507	TX	Zavala	rural	125	28.6%	1,297.41	0.096	53.9%
49001	UT	Beaver	rural	175	28.7%	2,589.88	0.068	88.3%
49003	UT	Box Elder	urban	745	28.6%	5745.55	0.130	54.4%
49005	UT	Cache	urban	1,033	28.6%	1,164.81	0.887	43.1%
49007	UT	Carbon	rural	552	28.4%	1,478.49	0.373	86.9%
49011	UT	Davis	urban	3,342	28.6%	298.78	11.184	51.0%
49013	UT	Duchesne	rural	333	28.5%	3,240.94	0.103	65.1%
49015	UT	Emery	rural	289	28.5%	4,462.31	0.065	89.7%
49017	UT	Garfield	rural	186	28.2%	5,175.12	0.036	90.1%
49019	UT	Grand	rural	313	28.7%	3,671.54	0.085	88.8%

49021	UT	Iron	rural	953	28.7%	3,296.68	0.289	70.0%
49023	UT	Juab	urban	231	28.6%	3,392.28	0.068	89.0%
49025	UT	Kane	rural	311	28.9%	3,990.23	0.078	87.8%
49027	UT	Millard	rural	359	28.6%	6,572.42	0.055	90.8%
49029	UT	Morgan	urban	171	28.3%	609.20	0.281	59.5%
49035	UT	Salt Lake	urban	10,764	28.6%	742.28	14.501	44.2%
49037	UT	San Juan	rural	273	28.6%	7819.99	0.035	87.4%
49039	UT	Sanpete	rural	627	28.5%	1,590.15	0.394	82.1%
49041	UT	Sevier	rural	553	28.8%	1,910.58	0.289	84.2%
49043	UT	Summit	rural	662	28.4%	1,871.71	0.354	61.4%
49045	UT	Tooele	urban	656	28.7%	6,941.35	0.095	60.2%
49047	UT	Uintah	rural	400	28.3%	4,479.69	0.089	59.8%
49049	UT	Utah	urban	4,396	28.7%	2,003.45	2.194	48.5%
49051	UT	Wasatch	rural	422	28.4%	1,175.50	0.359	51.0%
49053	UT	Washington	urban	4,233	28.7%	2,426.36	1.744	67.3%
49055	UT	Wayne	rural	103	29.0%	2,460.68	0.042	90.6%
49057	UT	Weber	urban	3,279	28.6%	576.08	5.691	53.9%
51001	VA	Accomack	rural	1,107	28.4%	449.50	2.462	77.0%
51003	VA	Albemarle	urban	1,567	28.5%	720.70	2.174	79.4%
51510	VA	Alexandria	urban	2,612	28.5%	15.03	173.758	69.2%
51005	VA	Alleghany	rural	207	28.6%	445.46	0.465	78.8%
51007	VA	Amelia	urban	309	28.5%	355.27	0.869	69.0%
51009	VA	Amherst	urban	840	28.3%	473.93	1.772	75.9%
51011	VA	Appomattox	urban	376	28.3%	333.49	1.126	79.1%
51013	VA	Arlington	urban	3,014	28.6%	25.97	116.069	66.5%
51015	VA	Augusta	urban	1,362	28.7%	967.00	1.408	78.7%
51017	VA	Bath	rural	211	28.8%	529.16	0.398	86.7%
51019	VA	Bedford	urban	2,124	28.6%	753.02	2.820	75.9%
51021	VA	Bland	rural	181	28.6%	357.72	0.505	73.1%
51023	VA	Botetourt	urban	924	28.5%	541.20	1.708	70.7%
51520	VA	Bristol	urban	571	28.5%	13.01	43.924	48.4%
51025	VA	Brunswick	rural	407	27.5%	566.17	0.718	75.3%
51027	VA	Buchanan	rural	380	28.4%	502.76	0.756	56.4%
51029	VA	Buckingham	urban	282	27.9%	579.66	0.487	74.0%
51530	VA	Buena Vista	rural	207	28.6%	6.7	30.952	71.8%
51031	VA	Campbell	urban	1,261	28.5%	503.87	2.502	75.4%
51033	VA	Caroline	urban	561	28.1%	527.51	1.064	73.1%
51035	VA	Carroll	rural	739	28.6%	474.69	1.556	80.2%
51036	VA	Charles City	urban	239	28.0%	182.82	1.305	68.3%
51037	VA	Charlotte	rural	472	28.3%	475.27	0.993	77.8%
51540	VA	Charlottesville	urban	2,119	28.6%	10.24	206.935	78.4%
51550	VA	Chesapeake	urban	3,643	28.4%	340.80	10.690	69.7%
51041	VA	Chesterfield	urban	5,461	28.5%	423.30	12.901	67.5%
51043	VA	Clarke	urban	400	28.5%	176.18	2.269	79.1%
51570	VA	Colonial Heights	urban	589	28.7%	7.52	78.375	75.7%
51580	VA	Covington	rural	477	28.5%	5.47	87.294	83.2%
51045	VA	Craig	urban	114	28.0%	329.53	0.345	72.5%
51047	VA	Culpeper	urban	1,094	28.5%	379.23	2.886	77.5%
51049	VA	Cumberland	rural	148	28.1%	297.46	0.498	70.5%
51590	VA	Danville	rural	1,634	28.3%	42.93	38.052	76.7%
51051	VA	Dickenson	rural	279	28.4%	330.53	0.845	59.2%
51053	VA	Dinwiddie	urban	396	28.3%	503.72	0.786	71.7%
51595	VA	Emporia	rural	257	28.2%	6.89	37.247	67.2%
51057	VA	Essex	rural	321	28.2%	257.12	1.250	74.3%
51059	VA	Fairfax	urban	15,970	28.6%	390.97	40.848	66.0%
51600	VA	Fairfax	urban	2,445	28.7%	6.24	391.796	66.5%
51610	VA	Falls Church	urban	325	28.8%	2	162.449	62.9%
51061	VA	Fauquier	urban	1,572	28.6%	647.45	2.428	76.9%
51063	VA	Floyd	urban	424	28.6%	380.42	1.115	74.2%
51065	VA	Fluvanna	urban	724	28.7%	286.01	2.532	76.3%
51067	VA	Franklin	urban	1,199	28.6%	690.43	1.737	68.9%
51620	VA	Franklin	rural	351	28.3%	8.21	42.754	81.2%
51069	VA	Frederick	urban	1,252	28.7%	413.50	3.027	79.0%
51630	VA	Fredericksburg	urban	906	28.3%	10.44	86.792	80.5%
51640	VA	Galax	rural	536	28.7%	8.24	65.065	85.3%
51071	VA	Giles	urban	467	28.5%	355.78	1.311	69.3%
51073	VA	Gloucester	urban	1,024	28.6%	217.81	4.700	76.2%
51075	VA	Goochland	urban	464	28.6%	281.42	1.649	70.9%
51077	VA	Grayson	rural	480	28.8%	442.17	1.085	84.0%
51079	VA	Greene	urban	458	28.6%	156.25	2.934	78.6%
51083	VA	Halifax	rural	1,068	28.1%	817.84	1.305	79.2%
51650	VA	Hampton	urban	2,516	28.2%	51.41	48.948	68.5%
51085	VA	Hanover	urban	2,865	28.6%	468.54	6.115	70.6%
51660	VA	Harrisonburg	urban	818	28.6%	17.42	46.940	78.0%
51087	VA	Henrico	urban	4,072	28.6%	233.70	17.425	69.9%
51089	VA	Henry	rural	1,114	28.7%	382.33	2.913	66.1%
51091	VA	Highland	rural	113	28.8%	415.16	0.271	86.2%
51670	VA	Hopewell	urban	530	28.5%	10.28	51.575	77.0%
51093	VA	Isle of Wight	urban	854	28.4%	315.61	2.707	70.6%
51095	VA	James City	urban	817	28.8%	142.44	5.738	79.1%
51099	VA	King George	rural	415	28.4%	179.64	2.311	75.3%
51101	VA	King William	urban	376	28.3%	273.94	1.374	78.7%

51097	VA	King and Queen	rural	190	28.2%	315.14	0.603	77.6%
51103	VA	Lancaster	rural	724	28.6%	133.25	5.432	82.8%
51105	VA	Lee	rural	431	28.7%	435.52	0.990	55.4%
51678	VA	Lexington	rural	583	28.6%	2.50	233.150	81.6%
51107	VA	Loudoun	urban	4,172	28.7%	515.56	8.092	68.0%
51109	VA	Louisa	rural	933	28.5%	496.30	1.879	75.6%
51111	VA	Lunenburg	rural	266	28.2%	431.68	0.616	77.9%
51680	VA	Lynchburg	urban	2,148	28.4%	49.13	43.730	76.8%
51113	VA	Madison	rural	338	28.5%	320.68	1.055	79.2%
51683	VA	Manassas	urban	1,876	28.7%	9.88	189.897	66.1%
51690	VA	Martinsville	rural	860	28.5%	10.96	78.423	68.6%
51115	VA	Mathews	urban	428	28.5%	85.93	4.982	81.5%
51117	VA	Mecklenburg	rural	1,045	28.4%	625.49	1.671	75.9%
51119	VA	Middlesex	rural	518	28.6%	130.31	3.972	78.7%
51121	VA	Montgomery	urban	1,662	28.6%	387.01	4.293	74.4%
51125	VA	Nelson	urban	685	28.4%	470.86	1.455	79.2%
51127	VA	New Kent	urban	454	28.3%	209.73	2.163	74.3%
51700	VA	Newport News	urban	2,963	28.4%	68.71	43.124	68.9%
51710	VA	Norfolk	urban	2,970	28.2%	54.12	54.869	65.8%
51131	VA	Northampton	rural	423	28.2%	211.61	2.001	76.7%
51133	VA	Northumberland	rural	600	28.7%	191.30	3.134	80.2%
51720	VA	Norton	rural	114	28.6%	7.48	15.198	58.9%
51135	VA	Nottoway	rural	386	28.2%	314.39	1.228	74.8%
51137	VA	Orange	rural	1,295	28.6%	340.78	3.801	79.1%
51139	VA	Page	rural	697	28.7%	310.86	2.241	80.5%
51141	VA	Patrick	rural	583	28.6%	483.10	1.208	69.9%
51730	VA	Petersburg	urban	755	28.1%	22.93	32.908	69.2%
51143	VA	Pittsylvania	rural	1,254	28.4%	968.94	1.294	75.1%
51735	VA	Poquoson	urban	244	28.6%	15.32	15.911	80.5%
51740	VA	Portsmouth	urban	1,497	28.0%	33.65	44.500	64.3%
51145	VA	Powhatan	urban	736	28.4%	260.22	2.830	70.6%
51147	VA	Prince Edward	rural	575	28.0%	349.96	1.644	75.6%
51149	VA	Prince George	urban	436	28.4%	265.16	1.644	78.9%
51153	VA	Prince William	urban	3,860	28.8%	336.4	11.473	67.4%
51155	VA	Pulaski	urban	853	28.6%	319.86	2.666	75.3%
51750	VA	Radford	urban	396	28.9%	9.87	40.135	74.0%
51157	VA	Rappahannock	urban	390	28.5%	266.23	1.466	80.5%
51159	VA	Richmond	rural	229	28.5%	191.49	1.198	74.1%
51760	VA	Richmond	urban	5,838	28.4%	59.81	97.604	61.0%
51161	VA	Roanoke	urban	1,281	28.6%	250.52	5.113	76.2%
51770	VA	Roanoke	urban	3,464	28.5%	42.56	81.399	69.2%
51163	VA	Rockbridge	rural	396	28.7%	597.56	0.663	77.4%
51165	VA	Rockingham	urban	1,705	28.6%	849.09	2.008	76.6%
51167	VA	Russell	rural	560	28.5%	473.82	1.182	63.6%
51775	VA	Salem	urban	1,078	28.5%	14.44	74.679	74.4%
51169	VA	Scott	urban	388	28.3%	535.53	0.725	38.7%
51171	VA	Shenandoah	rural	1,396	28.8%	508.78	2.744	80.1%
51173	VA	Smyth	rural	856	28.5%	450.93	1.899	71.5%
51175	VA	Southampton	rural	315	28.2%	599.14	0.526	76.5%
51177	VA	Spotsylvania	urban	1,427	28.7%	401.50	3.555	76.2%
51179	VA	Stafford	urban	2,326	28.8%	268.96	8.647	75.9%
51790	VA	Staunton	urban	1,215	28.6%	19.98	60.793	82.2%
51800	VA	Suffolk	urban	1,595	28.4%	400.17	3.985	72.1%
51181	VA	Surry	rural	146	28.5%	278.95	0.524	65.9%
51183	VA	Sussex	urban	272	28.2%	490.22	0.554	70.7%
51185	VA	Tazewell	rural	1,001	28.5%	518.85	1.928	64.8%
51810	VA	Virginia Beach	urban	8,215	28.6%	249.02	32.987	74.3%
51187	VA	Warren	urban	892	28.5%	213.47	4.177	80.5%
51191	VA	Washington	urban	1,153	28.6%	560.97	2.055	62.8%
51820	VA	Waynesboro	urban	945	28.7%	15.04	62.831	79.3%
51193	VA	Westmoreland	rural	590	28.5%	229.37	2.572	79.0%
51830	VA	Williamsburg	urban	1,128	28.6%	9.02	125.110	79.4%
51840	VA	Winchester	urban	1,513	28.7%	9.23	163.899	78.2%
51195	VA	Wise	rural	702	28.5%	403.19	1.742	57.0%
51197	VA	Wythe	rural	929	28.6%	461.82	2.012	75.2%
51199	VA	York	urban	2,965	28.7%	104.78	28.298	79.8%
50001	VT	Addison	rural	1,063	28.5%	766.33	1.387	78.2%
50003	VT	Bennington	rural	1,359	28.7%	674.98	2.013	83.6%
50005	VT	Caledonia	rural	1,010	28.6%	648.86	1.556	83.1%
50007	VT	Chittenden	urban	3,109	28.6%	536.58	5.794	80.3%
50009	VT	Essex	rural	266	28.3%	663.60	0.402	86.0%
50011	VT	Franklin	urban	1,128	28.6%	633.71	1.780	81.0%
50013	VT	Grand Isle	urban	258	28.5%	81.81	3.158	79.6%
50015	VT	Lamoille	rural	1,593	28.6%	458.80	3.471	78.9%
50017	VT	Orange	rural	934	28.6%	687.03	1.359	82.6%
50019	VT	Orleans	rural	990	28.6%	693.27	1.428	83.3%
50021	VT	Rutland	rural	2,256	28.5%	929.82	2.426	84.2%
50023	VT	Washington	rural	1,937	28.6%	687.23	2.819	81.9%
50025	VT	Windham	rural	1,472	28.7%	785.31	1.875	82.5%
50027	VT	Windsor	rural	2,147	28.6%	969.34	2.215	81.1%
53001	WA	Adams	rural	216	28.4%	1,924.98	0.112	81.3%
53003	WA	Asotin	urban	713	28.5%	636.21	1.121	75.1%

53005	WA	Benton	urban	4,067	28.6%	1,700.38	2.392	79.3%
53007	WA	Chelan	urban	1,575	28.5%	2,920.52	0.539	71.7%
53009	WA	Clallam	rural	3,995	28.7%	1,738.33	2.298	86.6%
53011	WA	Clark	urban	4,811	28.5%	629.00	7.649	29.8%
53013	WA	Columbia	urban	171	28.8%	868.63	0.197	89.3%
53015	WA	Cowlitz	urban	1,697	28.6%	1,140.13	1.489	41.1%
53017	WA	Douglas	urban	1,536	28.5%	1,819.26	0.844	69.0%
53019	WA	Ferry	rural	276	28.8%	2,203.16	0.125	87.1%
53021	WA	Franklin	urban	1,108	28.6%	1,242.17	0.892	78.1%
53023	WA	Garfield	rural	105	28.3%	710.69	0.148	92.7%
53025	WA	Grant	rural	1,879	28.5%	2,679.51	0.701	75.4%
53027	WA	Grays Harbor	rural	2,424	28.5%	1902.03	1.274	84.6%
53029	WA	Island	rural	2,673	28.8%	208.45	12.825	61.4%
53031	WA	Jefferson	rural	2,039	28.7%	1,803.70	1.130	87.9%
53033	WA	King	urban	30,846	28.5%	2,115.57	14.580	50.6%
53035	WA	Kitsap	urban	6,346	28.6%	394.94	16.068	67.0%
53037	WA	Kittitas	rural	1,165	28.6%	2,297.27	0.507	84.9%
53039	WA	Klickitat	rural	853	28.4%	1,871.31	0.456	85.7%
53041	WA	Lewis	rural	1,975	28.6%	2,402.80	0.822	58.1%
53043	WA	Lincoln	rural	476	28.6%	2,310.49	0.206	90.5%
53045	WA	Mason	rural	1,943	28.6%	959.42	2.025	69.1%
53047	WA	Okanogan	rural	1,488	28.4%	5,267.98	0.282	82.9%
53049	WA	Pacific	rural	1,063	28.8%	932.66	1.140	90.1%
53051	WA	Pend Oreille	urban	512	28.7%	1399.99	0.365	81.7%
53053	WA	Pierce	urban	14,131	28.5%	1,669.51	8.464	58.6%
53055	WA	San Juan	rural	829	28.6%	173.91	4.768	69.8%
53057	WA	Skagit	urban	3,295	28.7%	1,731.20	1.903	59.9%
53059	WA	Skamania	urban	267	28.6%	1,655.68	0.162	78.5%
53061	WA	Snohomish	urban	8,550	28.6%	2,087.27	4.096	41.0%
53063	WA	Spokane	urban	9,571	28.5%	1,763.79	5.426	55.8%
53065	WA	Stevens	urban	1,501	28.5%	2,477.76	0.606	81.4%
53067	WA	Thurston	urban	5,263	28.7%	721.96	7.289	52.6%
53069	WA	Wahkiakum	rural	166	28.7%	263.38	0.629	64.6%
53071	WA	Walla Walla	urban	1,723	28.4%	1270.13	1.356	80.0%
53073	WA	Whatcom	urban	4,136	28.5%	2,106.86	1.963	52.3%
53075	WA	Whitman	rural	819	28.7%	2,159.09	0.379	85.4%
53077	WA	Yakima	urban	4,695	28.5%	4,295.40	1.093	71.9%
55001	WI	Adams	rural	404	28.8%	645.65	0.626	67.8%
55003	WI	Ashland	rural	417	28.6%	1,045.04	0.399	56.5%
55005	WI	Barron	rural	1,129	28.4%	862.71	1.308	53.6%
55007	WI	Bayfield	rural	447	28.7%	1,477.86	0.302	59.1%
55009	WI	Brown	urban	3,050	28.5%	529.71	5.757	37.2%
55011	WI	Buffalo	rural	423	28.3%	671.64	0.630	69.4%
55013	WI	Burnett	rural	479	28.4%	821.85	0.583	57.9%
55015	WI	Calumet	urban	250	28.2%	318.24	0.785	24.8%
55017	WI	Chippewa	urban	1,349	28.4%	1,008.37	1.338	59.4%
55019	WI	Clark	rural	515	28.3%	1,209.82	0.426	40.2%
55021	WI	Columbia	urban	1,448	28.5%	765.53	1.892	60.8%
55023	WI	Crawford	rural	398	28.6%	570.66	0.698	57.1%
55025	WI	Dane	urban	9,409	28.6%	1,197.24	7.859	64.5%
55027	WI	Dodge	rural	1,342	28.6%	875.62	1.532	57.4%
55029	WI	Door	rural	1,120	28.5%	481.98	2.323	66.7%
55031	WI	Douglas	urban	839	28.3%	1,304.14	0.643	49.2%
55033	WI	Dunn	rural	747	28.4%	850.11	0.879	56.2%
55035	WI	Eau Claire	urban	2,105	28.5%	637.98	3.299	61.1%
55037	WI	Florence	rural	107	28.1%	488.20	0.220	63.1%
55039	WI	Fond du Lac	urban	1,649	28.5%	719.55	2.291	40.7%
55041	WI	Forest	rural	257	28.7%	1,014.07	0.253	55.7%
55043	WI	Grant	rural	1,131	28.5%	1,146.85	0.987	53.9%
55045	WI	Green	urban	1,000	28.6%	583.96	1.713	72.2%
55047	WI	Green Lake	rural	354	28.4%	349.44	1.013	37.3%
55049	WI	Iowa	urban	419	28.1%	762.58	0.549	52.8%
55051	WI	Iron	rural	168	28.5%	758.17	0.222	49.6%
55053	WI	Jackson	rural	344	28.4%	987.72	0.348	47.7%
55055	WI	Jefferson	rural	1,761	28.6%	556.47	3.164	59.8%
55057	WI	Juneau	rural	731	28.4%	766.93	0.953	67.6%
55059	WI	Kenosha	urban	2,667	28.6%	271.99	9.804	64.7%
55061	WI	Kewaunee	urban	359	28.4%	342.52	1.049	42.2%
55063	WI	La Crosse	urban	1,782	28.4%	451.69	3.946	42.9%
55065	WI	Lafayette	rural	349	28.3%	633.59	0.552	61.0%
55067	WI	Langlade	rural	454	28.5%	870.64	0.522	49.6%
55069	WI	Lincoln	rural	726	28.7%	878.97	0.826	49.6%
55071	WI	Manitowoc	rural	1,530	28.5%	589.08	2.597	46.4%
55073	WI	Marathon	urban	2,071	28.4%	1,544.98	1.340	44.0%
55075	WI	Marinette	rural	1,136	28.4%	1,399.35	0.812	52.6%
55077	WI	Marquette	rural	495	28.7%	455.60	1.087	56.1%
55079	WI	Milwaukee	urban	11,235	28.3%	241.40	46.542	47.1%
55081	WI	Monroe	rural	822	28.4%	900.78	0.912	54.2%
55083	WI	Oconto	urban	566	28.4%	997.99	0.567	38.6%
55085	WI	Oneida	rural	1,301	28.4%	1,112.97	1.169	58.9%
55087	WI	Outagamie	urban	1,935	28.6%	637.52	3.035	29.4%
55089	WI	Ozaukee	urban	1,873	28.6%	233.08	8.036	51.4%

55091	WI	Pepin	rural	229	28.6%	231.98	0.986	69.1%
55093	WI	Pierce	urban	719	28.3%	573.75	1.253	49.4%
55095	WI	Polk	rural	824	28.4%	913.96	0.902	49.5%
55097	WI	Portage	rural	1,143	28.5%	800.68	1.428	49.6%
55099	WI	Price	rural	406	28.3%	1,254.38	0.324	53.4%
55101	WI	Racine	urban	3,611	28.6%	332.50	10.861	53.5%
55103	WI	Richland	rural	472	28.5%	586.15	0.805	72.6%
55105	WI	Rock	urban	3,042	28.6%	718.14	4.236	58.0%
55107	WI	Rusk	rural	308	28.3%	913.59	0.337	48.8%
55111	WI	Sauk	rural	1,222	28.6%	830.90	1.470	52.0%
55113	WI	Sawyer	rural	494	28.5%	1,257.30	0.393	58.7%
55115	WI	Shawano	rural	596	28.5%	893.06	0.667	34.0%
55117	WI	Sheboygan	urban	2,025	28.5%	511.27	3.962	48.1%
55109	WI	St. Croix	urban	1,093	28.4%	722.33	1.513	47.5%
55119	WI	Taylor	rural	327	28.3%	974.88	0.335	46.7%
55121	WI	Trempealeau	rural	664	28.2%	732.96	0.906	51.1%
55123	WI	Vernon	rural	489	28.4%	791.58	0.618	36.9%
55125	WI	Vilas	rural	923	28.6%	856.60	1.078	64.4%
55127	WI	Walworth	rural	2,395	28.6%	555.13	4.315	70.5%
55129	WI	Washburn	rural	675	28.4%	797.11	0.847	55.4%
55131	WI	Washington	urban	2,560	28.7%	430.70	5.943	51.0%
55133	WI	Waukesha	urban	7,923	28.5%	549.57	14.417	50.8%
55135	WI	Waupaca	rural	935	28.5%	747.71	1.251	35.9%
55137	WI	Waushara	rural	425	28.4%	626.15	0.679	39.9%
55139	WI	Winnebago	urban	1,920	28.5%	434.49	4.420	34.0%
55141	WI	Wood	rural	1,473	28.5%	793.12	1.857	38.6%
54001	WV	Barbour	rural	324	28.7%	341.06	0.949	64.5%
54003	WV	Berkeley	urban	2,047	28.6%	321.14	6.375	66.8%
54005	WV	Boone	urban	408	28.4%	501.54	0.813	67.9%
54007	WV	Braxton	rural	280	28.3%	510.81	0.549	59.7%
54009	WV	Brooke	urban	405	28.4%	89.20	4.542	50.6%
54011	WV	Cabell	urban	2,043	28.5%	281.02	7.269	55.8%
54013	WV	Calhoun	rural	183	28.4%	279.25	0.655	69.0%
54015	WV	Clay	urban	199	28.5%	341.90	0.582	57.2%
54017	WV	Doddridge	rural	112	28.8%	319.72	0.349	60.8%
54019	WV	Fayette	urban	975	28.4%	661.55	1.474	63.7%
54021	WV	Gilmer	rural	138	28.4%	338.50	0.407	59.7%
54023	WV	Grant	rural	402	28.6%	477.37	0.841	67.3%
54025	WV	Greenbrier	rural	950	28.5%	1,019.57	0.932	69.5%
54027	WV	Hampshire	urban	580	28.5%	640.25	0.906	70.7%
54029	WV	Hancock	urban	880	28.5%	82.61	10.651	66.3%
54031	WV	Hardy	rural	335	28.4%	582.31	0.576	67.1%
54033	WV	Harrison	rural	1,549	28.5%	416.01	3.723	61.5%
54035	WV	Jackson	rural	643	28.4%	464.35	1.384	65.8%
54037	WV	Jefferson	urban	1,144	28.6%	209.63	5.457	65.6%
54039	WV	Kanawha	urban	3,733	28.4%	901.59	4.141	54.8%
54041	WV	Lewis	rural	370	28.5%	384.90	0.961	54.5%
54043	WV	Lincoln	urban	362	28.4%	437.04	0.828	56.9%
54045	WV	Logan	rural	669	28.3%	453.74	1.475	65.7%
54049	WV	Marion	rural	1,295	28.5%	308.74	4.193	61.6%
54051	WV	Marshall	urban	527	28.4%	305.43	1.726	48.0%
54053	WV	Mason	rural	589	28.6%	430.75	1.367	69.0%
54047	WV	McDowell	rural	375	28.2%	533.46	0.703	64.9%
54055	WV	Mercer	rural	1,326	28.4%	418.99	3.165	64.4%
54057	WV	Mineral	urban	652	28.4%	327.83	1.987	75.9%
54059	WV	Mingo	rural	414	28.2%	423.11	0.978	64.5%
54061	WV	Monongalia	urban	1,203	28.5%	360.06	3.340	53.8%
54063	WV	Monroe	rural	441	28.5%	472.75	0.932	71.1%
54065	WV	Morgan	rural	501	28.8%	229.07	2.186	74.9%
54067	WV	Nicholas	rural	557	28.3%	646.82	0.862	57.7%
54069	WV	Ohio	urban	933	28.3%	105.82	8.817	45.1%
54071	WV	Pendleton	rural	227	28.6%	696.05	0.327	67.2%
54073	WV	Pleasants	rural	152	28.4%	130.10	1.167	60.6%
54075	WV	Pocahontas	rural	229	28.4%	940.28	0.243	66.6%
54077	WV	Preston	urban	760	28.6%	648.80	1.172	67.1%
54079	WV	Putnam	urban	905	28.6%	345.67	2.619	54.3%
54081	WV	Raleigh	urban	1,825	28.4%	605.35	3.015	70.0%
54083	WV	Randolph	rural	754	28.8%	1,039.68	0.725	67.4%
54085	WV	Ritchie	rural	244	28.6%	451.99	0.540	64.0%
54087	WV	Roane	rural	302	28.2%	483.56	0.624	61.2%
54089	WV	Summers	rural	289	28.3%	360.46	0.801	68.8%
54091	WV	Taylor	rural	337	28.6%	172.77	1.953	67.8%
54093	WV	Tucker	rural	179	28.7%	418.92	0.428	60.4%
54095	WV	Tyler	rural	165	27.9%	256.29	0.643	57.3%
54097	WV	Upshur	rural	498	28.5%	354.64	1.403	55.5%
54099	WV	Wayne	urban	595	28.4%	505.98	1.176	59.2%
54101	WV	Webster	rural	211	28.5%	553.47	0.382	61.3%
54103	WV	Wetzel	rural	406	28.5%	358.06	1.133	51.7%
54105	WV	Wirt	urban	130	28.5%	232.51	0.558	62.0%
54107	WV	Wood	urban	2,107	28.5%	366.26	5.753	67.7%
54109	WV	Wyoming	rural	429	28.2%	499.45	0.858	65.2%
56001	WY	Albany	rural	724	28.5%	4,273.84	0.170	84.0%

56003	WY	Big Horn	rural	421	28.6%	3137.1	0.134	91.7%
56005	WY	Campbell	rural	695	28.5%	4,802.71	0.145	86.1%
56007	WY	Carbon	rural	439	28.6%	7,897.58	0.056	88.0%
56009	WY	Converse	rural	397	28.7%	4,254.88	0.093	91.2%
56011	WY	Crook	rural	265	28.8%	2,854.41	0.093	87.6%
56013	WY	Fremont	rural	1,271	28.5%	9,183.81	0.138	84.7%
56015	WY	Goshen	rural	528	28.6%	2,225.39	0.237	92.1%
56017	WY	Hot Springs	rural	226	28.4%	2004.09	0.113	94.4%
56019	WY	Johnson	rural	365	28.6%	4,154.15	0.088	90.3%
56021	WY	Laramie	urban	2,712	28.5%	2,685.91	1.010	88.6%
56023	WY	Lincoln	rural	583	28.6%	4,076.13	0.143	89.5%
56025	WY	Natrona	urban	2,045	28.5%	5,340.35	0.383	87.0%
56027	WY	Niobrara	rural	104	28.4%	2,626.04	0.040	90.1%
56029	WY	Park	rural	1,279	28.4%	6,942.08	0.184	92.3%
56031	WY	Platte	rural	403	28.5%	2,084.21	0.193	91.6%
56033	WY	Sheridan	rural	1,110	28.7%	2,523.99	0.440	85.9%
56035	WY	Sublette	rural	249	28.4%	4,886.54	0.051	89.5%
56037	WY	Sweetwater	rural	868	28.6%	10,426.65	0.083	88.0%
56039	WY	Teton	rural	611	28.7%	3,995.38	0.153	87.3%
56041	WY	Uinta	rural	391	28.5%	2,081.26	0.188	78.1%
56043	WY	Washakie	rural	298	28.3%	2,238.55	0.133	89.3%
56045	WY	Weston	rural	246	28.6%	2,398.09	0.103	85.9%
